# Addressing brain metabolic connectivity in treatment-resistant schizophrenia: a novel graph theory-driven application of ^18^F-FDG-PET with antipsychotic dose correction

**DOI:** 10.1038/s41537-024-00535-4

**Published:** 2024-12-19

**Authors:** Giuseppe De Simone, Felice Iasevoli, Annarita Barone, Valeria Gaudieri, Alberto Cuocolo, Mariateresa Ciccarelli, Sabina Pappatà, Andrea de Bartolomeis

**Affiliations:** 1 Section of Psychiatry, Laboratory of Molecular and Translational Psychiatry, Unit of Treatment-Resistant Psychiatric Disorders, Department of Neuroscience, Reproductive Sciences and Dentistry, University of Naples “Federico II”, School of Medicine, Naples Italy, Via Pansini 5, 80131 Naples, Italy; 2 Department of Advanced Biomedical Sciences, University of Naples Federico II, Via S. Pansini 5, 80131 Naples, Italy; 3 Institute of Biostructure and Bioimaging, National Research Council, Via T. De Amicis 95, 80145 Naples, Italy

**Keywords:** Schizophrenia, Neural circuits

## Abstract

Few studies using Positron Emission Tomography with ^18^F-fluorodeoxyglucose (^18^F-FDG-PET) have examined the neurobiological basis of antipsychotic resistance in schizophrenia, primarily focusing on metabolic activity, with none investigating connectivity patterns. Here, we aimed to explore differential patterns of glucose metabolism between patients and controls (CTRL) through a graph theory-based approach and network comparison tests. PET scans with ^18^F-FDG were obtained by 70 subjects, 26 with treatment-resistant schizophrenia (TRS), 28 patients responsive to antipsychotics (nTRS), and 16 CTRL. Relative brain glucose metabolism maps were processed in the automated anatomical labeling (AAL)-Merged atlas template. Inter-subject connectivity matrices were derived using Gaussian Graphical Models and group networks were compared through permutation testing. A logistic model based on machine-learning was employed to estimate the association between the metabolic signals of brain regions and treatment resistance. To account for the potential influence of antipsychotic medication, we incorporated chlorpromazine equivalents as a covariate in the network analysis during partial correlation calculations. Additionally, the machine-learning analysis employed medication dose-stratified folds. Global reduced connectivity was detected in the nTRS (*p*-value = 0.008) and TRS groups (*p*-value = 0.001) compared to CTRL, with prominent alterations localized in the frontal lobe, Default Mode Network, and dorsal dopamine pathway. Disruptions in frontotemporal and striatal-cortical connectivity were detected in TRS but not nTRS patients. After adjusting for antipsychotic doses, alterations in the anterior cingulate, frontal and temporal gyri, hippocampus, and precuneus also emerged. The machine-learning approach demonstrated an accuracy ranging from 0.72 to 0.8 in detecting the TRS condition.

## Introduction

The lack of response to antipsychotics, affecting nearly 30% of schizophrenia patients^1,2^, leads to increased symptom severity and everyday functional impairment^3^. The present study marks the first attempt to address treatment-resistant schizophrenia (TRS) by a metabolic connectivity analysis based on ^18^F-fluorodeoxyglucose (^18^F-FDG)-Positron Emission Tomography (PET). As a further step, we applied a machine-learning approach aimed at classifying treatment-resistant patients from responders to antipsychotics (nTRS). The novelty lies in tackling the connectivity issue through a functional imaging technique, utilizing a direct measure of regional brain glucose metabolism for highlighting potential alterations in brain networks and instructing a machine-learning algorithm. This approach is particularly meaningful as it is embedded in one of the most recognized milieu of schizophrenia pathology, conceptualized as a disorder of synapse and connectivity^4,5^.

Dysregulated processing and integration of neuronal responses at the synaptic level may affect the overall brain connectivity and/or the functionality of specific networks, including the Default Mode Network (DMN)^6–11^. For instance, disruptions in the function of glutamate receptors and changes in postsynaptic density elements can impair synaptic signaling, leading to reduced connectivity between neuronal networks^5,7^. Several areas have been identified as potential stratifiers between nTRS and healthy controls, including fronto-temporal and fronto-occipital regions, which exhibit differences in structural covariance networks^12^. Recent findings have highlighted a significant relationship between treatment refractoriness and more progressed stages of brain morphology patterns, as identified through a data-driven machine learning approach^13^. PET studies based on ^18^F-DOPA and ^11^C-raclopride signal, as well as functional magnetic resonance imaging (fMRI), revealed altered hubs of dopaminergic networks, particularly the dorsal striatum, and frontostriatal connectivity in schizophrenia subjects, with differences between individuals responsive and non-responsive to treatment^14–19^.

Most research on functional connectivity has predominantly utilized fMRI protocols based on the blood oxygenation level-dependent (BOLD) signal. While PET with ^18^F-FDG directly reflects neuronal metabolic activity^20^, the neurovascular coupling underlying the hemodynamic response in fMRI is influenced by a complex interplay between local cerebral blood flow, blood volume, and the cerebral metabolic rate of oxygen^20^. Simulation studies of fMRI connectivity have shown that aberrant connectivity in brain disorders reflects not only abnormal neural activity but also alterations in brain vessels and associated hemodynamic/metabolic pathophysiology^20,21^. When ^18^F-FDG-PET and fMRI data were simultaneously acquired from the same participants, PET data demonstrated a higher proportion of variance explained by fewer components compared to fMRI data^22^. This higher variance concentration suggests that PET results may be more robust and replicable across samples^22^. Overall, these findings along with previous brain network analysis based on ^18^F-FDG-PET enhance the potential utility of metabolic connectivity by highlighting its validity and replicability^20,23–28^.

In ^18^F-FDG-PET connectivity analyses, connectivity is assessed through intersubject estimation by analyzing covariation in signal intensity across participants, as only one static PET image per subject is available^20,27^. Network measures and graph metrics in the context of ^18^F-FDG-PET reflect these intersubject covariations, meaning that regions with correlated metabolic activity across subjects belong to the same functional network^20,23,26,27^. This methodology could be particularly valuable for exploring group-level connectivity patterns in disorders like schizophrenia, where metabolic alterations have been identified^29–36^.

While connectivity studies have employed ^18^F-FDG-PET to compute network measures and conduct graph theory analyses in neurological conditions, there remains a gap in its application to psychotic disorders, particularly schizophrenia^23–28^. Integrating a metabolic-constructed map with the existing information predominantly based on fMRI, which primarily reflects neurovascular coupling, could offer a novel perspective on aberrant connectivity in schizophrenia, potentially yielding a more comprehensive understanding of the disease through the use of multimodal techniques.

In this framework, we investigated metabolic connectivity in schizophrenia patients with the following objectives: i) to characterize connectivity patterns in patients and controls by providing a descriptive analysis and graph representation of brain networks; ii) to statistically compare network parameters between groups using permutation-based methods; iii) to perform a stratification of TRS and nTRS based on a pilot machine-learning approach trained with the metabolic signals of brain regions; and iv) to address the potential impact of antipsychotic dosage in detecting differences among groups.

## Methods

### Data acquisition and preprocessing

^18^F-FDG-PET scans were obtained by 70 participants, including 54 patients (28 nTRS and 26 TRS patients) and 16 age-matched controls (CTRL). Patients were diagnosed using the Structured Clinical Interview for Diagnosis (SCID-5), and categorized in nTRS/TRS based on TRRIP (Treatment Response and Resistance in Psychosis) Working Group consensus guidelines^2^. Inclusion criteria were: age 18–55 years, disease duration ≥2 years, no substantial medication changes within the past six months, and no worsening of psychotic symptoms within the last three months. Exclusion criteria encompassed the presence of macroscopic brain structural abnormalities, other psychiatric disorders, and severe medical conditions. All participants completed a screening process to ensure eligibility for the PET scan. Patients with specific contraindications were not recruited, such as pregnancy, allergies to the radiotracer, uncontrolled diabetes, recent major surgery, systemic inflammatory diseases, and severe kidney problems.

The sample size calculation was based on previous studies that demonstrated effective detection of circuit-level differences through ^18^F-FDG-PET network analysis^26^. ^18^F-FDG-PET scans were performed according to the European Association of Nuclear Medicine guidelines^37^. To prevent movement during the PET/CT acquisitions the head of the patients was fixed in a dedicated head holder and the patient was instructed to avoid any head movement. Static 15-minute brain images were acquired in 3D mode 45-min after the injection of 200–250 MBq of ^18^F-FDG using time-of-flight PET/CT system (Philips Ingenuity TF 64, Philips Medical Systems, Best, The Netherlands) with an axial field of view of 18 cm yielding 90 slices of 2 mm thickness and an axial and transaxial resolution (full width at half maximum [FWHM]) of 4.7 and 4.8 mm respectively. Static images were reconstructed with the iterative time-of-flight reconstruction algorithm (BLOB-OS-TF) and corrected for attenuation using CT scans. Reconstructed images were visually inspected to check for potential artefacts due to movements or to other technical factors. PET datasets were processed using the PMOD 3.9 version (PMOD Technologies LLC) and anatomically parcellated into 65 volumes of interest (VOIs) based on the automated anatomical labeling (AAL)-merged atlas defined in the MNI space (Supplementary T1). We selected the AAL atlas due to its use in previous brain metabolic connectivity studies and its balance between anatomical detail and a manageable number of regions, making it well-suited for high-dimensional data analysis^23,26^. Further, the use of the AAL atlas, which combines smaller regions to obtain larger volumes, likely mitigated some of the spatial resolution constraints inherent to PET imaging. Specific brain circuits, such as DMN and dorsal dopamine pathway, were chosen based on their established roles in schizophrenia and their well-characterized node composition in prior studies (Supplementary T2)^23,26,38^.

**Fig. 1:**
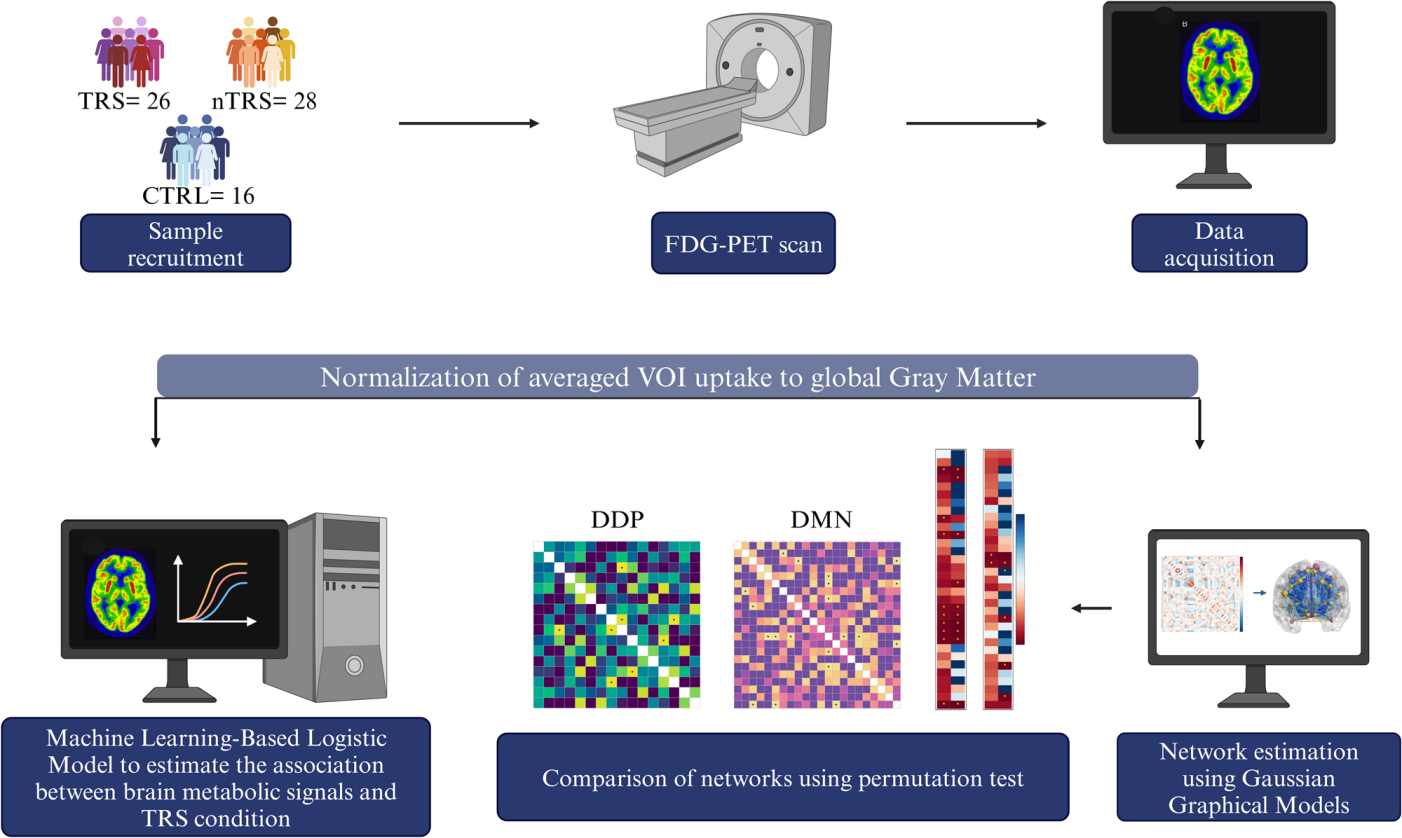
Schematic representation of the study workflow. Patients were recruited at the Department of Neuroscience, Reproductive Sciences and Dentistry, Unit of Treatment-Resistant Psychosis, University of Naples “Federico II”. PET scans were performed at the Department of Advanced Biomedical Sciences, Nuclear Medicine, University of Naples “Federico II”. VOIs were then transformed into the native ^18^F-FDG-PET space of each subject using the inverse transformation. Averaged VOI uptake values were normalized to global gray matter average values. Network estimations were based on GGMs combined with the atan penalty. Networks were compared across groups through permutation testing. Machine learning was used to stratify treatment-resistant and treatment-responsive patients. For a comprehensive acknowledgement, refer to the Method section.

This study, approved by the Ethical Committee for Clinical Studies of the University of Naples “Federico II” (protocol number: 195/19), significantly expands previously published data, incorporating approximately one-third more subjects^29^. All participants provided written informed consent prior to inclusion in the study. The study workflow is illustrated in Fig. 1.

### Graph theory application and network computation

Whole-brain connectivity matrices were constructed using partial correlation analysis. Functional connectivity research predominantly utilizes marginal correlation analysis, focusing on pairwise relationships between brain regions without considering third-party effects^27^. However, this approach may not effectively capture interactions among multiple brain regions working together^27^. To address this limitation, researchers have increasingly adopted partial correlation analysis, which assesses the association between pairs of brain regions while controlling for spurious associations by eliminating the influence of global or third-party effects on the pairwise correlation^27^. Estimating partial correlations typically involves maximum likelihood estimation (MLE) of the inverse covariance matrix. For reliable results, a significantly larger sample size (n) than the number of regions being studied (p) is necessary, a condition usually met in fMRI but not in PET studies^27,28^. When p exceeds n (high-dimensional data), the covariance matrix becomes non-invertible and ill-conditioned, resulting in substantial estimation errors^28,39^. To obtain trustworthy information, it is preferable to employ sparse inverse covariance estimation (SICE), known as Gaussian graphical models (GGMs), for a sparser matrix estimation, achieved by setting enough entries to zero^27,28^. The degree of sparsity is determined by the regularization parameter λ, which imposes a constraint on sparsity^27,28^. SICE is widely accepted as a valuable tool for determining the binary structure of an inverse covariance matrix, particularly in distinguishing between zero and non-zero entries^27,28^. Nevertheless, its reliability in estimating the actual values of non-zero entries is compromised by the shrinkage effect^27,28,39^. To tackle this issue, previous studies primarily utilized “quasi-measures” to assess the strength of functional connections^23,25,26^. Here, to obtain robust estimations of true brain networks and to mitigate the shrinkage effect, GGMs with a non-convex regularization approach, the arctangent type penalty (atan), were employed^40–42^. The atan penalty effectively shrinks smaller partial correlations towards zero while preserving larger partial correlations from the regularization process^41,42^. This characteristic of non-convex regularization is expected to yield nearly unbiased estimations, thereby enhancing predictive accuracy (Supplementary F1)^41,42^. The Atan-penalized least squares procedure has consistently demonstrated the ability to select the correct model^40^. Furthermore, when combined with a BIC-type criterion, the Atan procedure performs exceptionally well across a variety of settings^40^. Hence, we combined the Atan regularization with the BIC criterion to choose the tuning parameter, setting the hyperparameter γ to 0.01 in accordance with recommendations from prior research^40–42^.

### Descriptive analyses of networks’ properties

Several network properties were computed to qualitatively characterize the group networks. First, we measured network robustness by averaging the size of the largest connected component after sequentially removing nodes^43^. We analyzed the number of edges and edge density, computed as the ratio of the actual number of edges in the graph to the maximum possible number of edges, which indicate the level of connectivity within a network. The global clustering coefficient was used to measure the tendency of nodes to form tightly connected groups, indicating network’s integration capability^44^. The average shortest path length was calculated to assess the efficiency of information transfer within the network, with lower values indicating more efficient communication^44^. Finally, the small-worldness index was computed to assess how well the network balances local clustering with global efficiency, with lower values suggesting deviations from the ideal small-world structure often seen in healthy brain networks^45^.

To identify the subnetworks exhibiting alterations in connectivity, the average degree - defined as the average number of edges connected to each node - was calculated for each community within the brain graphs. This metric provides insight into the interconnectivity of nodes within the communities, enabling us to assess the degree to which alterations in connectivity may influence the overall network structure and functionality.

Hubs, which are high-degree nodes, were identified by selecting nodes with a participation coefficient one standard deviation above the mean, as previously described in the literature^23^. The participation coefficient quantifies a node’s engagement with various communities within the network^46^. When a node’s connections are exclusively within its own community, its participation coefficient is 0^46^. Conversely, if a node’s connections are evenly distributed across all communities, the participation coefficient reaches a maximum value approaching 1^46^. The Louvain method was employed to define modules within the network^23^. Networks computation was performed by the “GGMncv” package^41,42^ while descriptive analyses of brain graphs were made by “igraph”, “brainGraph” (https://cran.r-project.org/web/packages/brainGraph/), “clustAnalytics”, and “NetworkToolbox” packages^47–49^ in RStudio R version 4.1.2^50^. Brain networks were graphically represented by BrainNet Viewer^51^.

### Comparative analysis between group networks based on permutation testing

To conduct a statistical comparison between the networks of patients and CTRL, a permutation-based approach was utilized. Permutation tests, a subset of non-parametric statistics, proved particularly advantageous given the small sample sizes^52^. Permutation tests offer an exact method for analyzing high-dimensional data due to their distribution-free nature and flexibility^53^. In these tests, *p*-values are determined by assessing the positions of test statistics within an empirical null distribution created through random shuffling^53^. In previous studies, permutation testing proved to be an effective method for comparing biological networks^53–56^. The reliability and broad applicability of permutation testing were also demonstrated in prior network analyses of brain PET studies^54^. The “Network Comparison Test” function from the “GGMncv” package was employed for the permutation-based network comparison^41,42,57^. The absolute values of edge weights were considered for the statistical analysis. To ensure robust replicability, a high number of iterations (it = 10,000) was applied^54^. In all network comparison analyses, permuted *p*-values were adjusted for multiple testing and considered significant if below 0.05.

Comparative analyses of whole-brain networks included assessments of global strength, a measure of the overall connectivity of the network; nodes’ weighted degree, which reflects the importance of a node based on the weights of its connected edges; and paired edges, which are edges that connect the same pair of nodes. Together, these metrics provide a comprehensive understanding of network architecture, as they are cross-related and interdependent. Specifically, global strength is influenced by the centrality measures of nodes, which are determined by the presence of paired edges. This interconnectedness highlights how variations in one metric can impact the others, ultimately shaping the network’s overall structure and function.

Analyses were also conducted at the level of a priori defined networks, such as the DMN and dorsal dopamine pathway. The graphical output was generated using the “corrplot” package^58^ in RStudio R version 4.1.2^50^ and BrainNet Viewer^51^. To account for the potential effect of medication dosage on the observed correlations between cerebral regions, chlorpromazine equivalents were integrated into the network as a node. Network estimation and permutation-based differences were computed, incorporating the dosage effect into the analysis. Thus, the influence of medication dosage was considered when computing the edges between pairs of brain regions. Following the analysis, the medication dosage node and its connections were removed from the network.

While network comparison analyses were performed by comparing the three groups pairwise, the regression analyses, aimed at evaluating the potential impact of antipsychotic dosage on network parameters, were restricted to the TRS and nTRS groups.

### A machine-learning approach to explore patients’ resistance or responsiveness to treatment

Following recent guidelines for machine learning in mental health research, we carefully considered data quantity for selecting the applied algorithm and leveraged nested cross-validation to identify hyperparameter combinations that enhance model generalizability through the “nestedcv” package^59,60^. Nested cross-validation avoids the pooling of training and testing data, leading to an almost unbiased estimation of the model regardless of sample size^61,62^, as well as maximizes the utilization of the entire dataset to assess overall accuracy^59^. This method could be especially helpful when the number of variables is significantly larger than the sample size, as the application of other machine-learning techniques could lead to strongly biased performance estimations^59,61,62^.

The “nestedcv” package provides a function to partition the dataset into outer and inner loops^59^. The inner loop is responsible for tuning and validating the hyperparameter/model^59^. The best-performing model from the inner loop is subsequently tested on the unseen data from the outer fold, and this process is repeated across all outer folds^59^. We employed a Random Forest-based approach for hyperparameter tuning, specifically to optimize values such as alpha (α) and lambda (λ). Alpha controls the sparsity of the model, while lambda adjusts the overall strength of regularization. Following this, an additional cross-validation is conducted on the entire dataset to identify the final hyperparameters for the model, which are then used for predictions on external unseen data^59^. The number of inner folds, as well as the number of outer folds, was set at the default value of ten^59^.

To ensure balanced distribution of antipsychotic medication dosage across folds, we employed stratification based on chlorpromazine equivalents during nested cross-validation. In other terms, each fold represented a balanced range of chlorpromazine equivalents, preventing an overrepresentation of a specific dosage in any fold. This approach mitigated the risk of bias due to uneven distribution of antipsychotic doses across the dataset.

The variance and the 95% Confidence Intervals (95%C.I.) of the Area Under the ROC Curve (AUC) were computed by the “pROC” package^63^. The Brier Score was utilized as a measure of prediction accuracy to evaluate the performance of our prediction model.

## Results

Details on clinical and demographic features (Supplementary T3), along with correlation matrices before and after regularization (Supplementary F2–7), are represented in Supplementary Information.

### Graph theory application and assessment of network properties

**Fig. 2:**
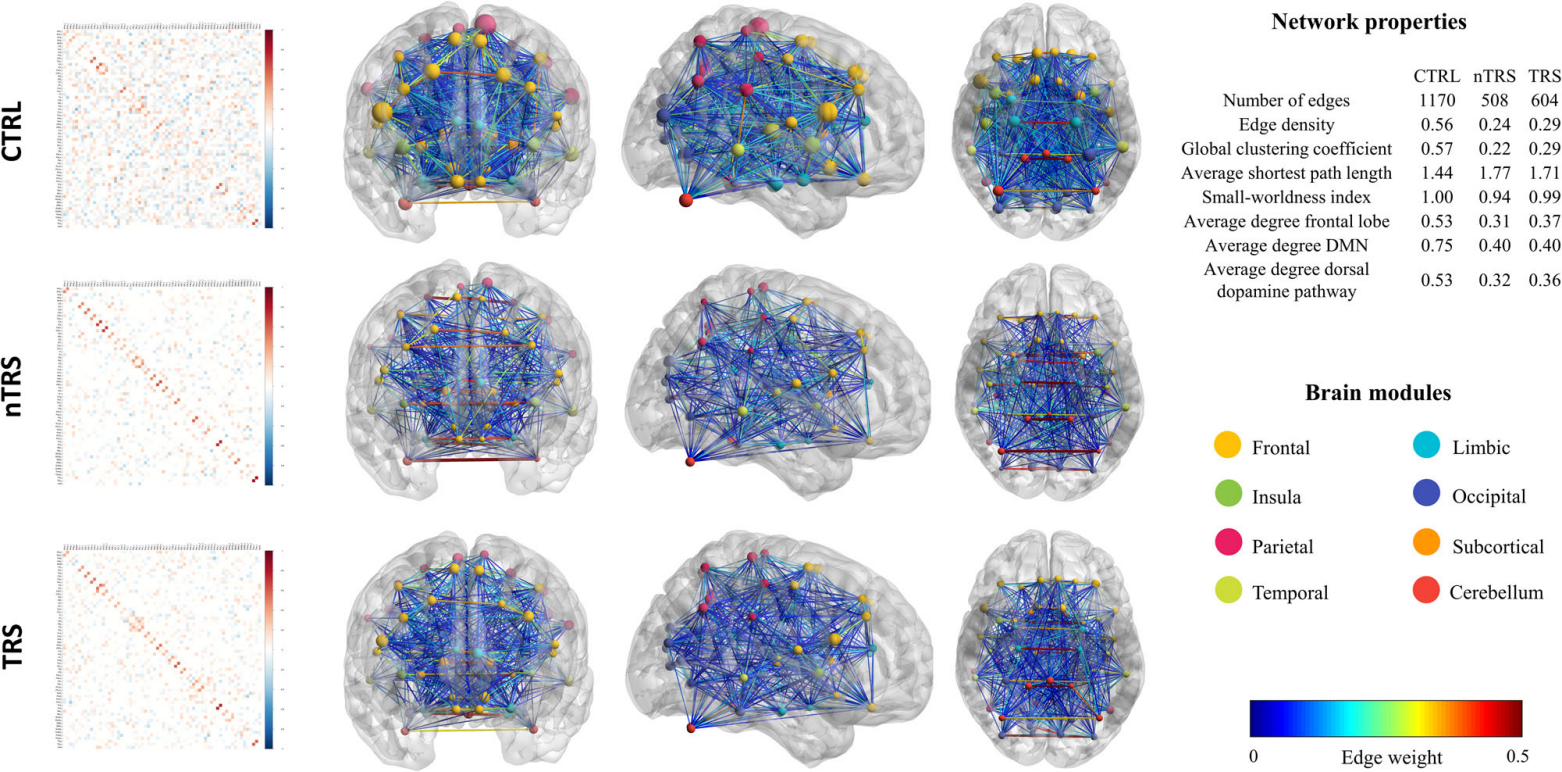
Brain graphs of CTRL, nTRS, and TRS groups are depicted alongside regularized correlation matrices. The scaling factor used in BrainNet Viewer is set to 1 for each group, and the node sizes reflect the weighted degree values without any group-specific scaling. Node colors indicate modules defined using the AAL-merged atlas. Edge size and color signify the strength of connections between pairs of brain regions. The analysis revealed that patients, when compared to CTRL, exhibit a widespread reduction in metabolic connectivity. This reduction is consistently observed in the frontal lobe, Default Mode Network, and Dorsal Dopamine pathway.

This section presents a qualitative, descriptive analysis of the metabolic networks, highlighting patterns and differences between the CTRL group and schizophrenia patients, without statistical testing, which is addressed in the following section. Robustness exhibited similarity across the groups (Supplementary T4). Both the nTRS and TRS groups showed lower global connectivity, as well as reduced integration and efficiency in communication than the CTRL group (Fig. 2). This was evidenced by network measures, such as the number of edges, edge density, global clustering coefficient, average shortest path length, and small-worldness index (Supplementary T5).

Altered connectivity primarily occurred within the frontal lobe (0.53 *vs* 0.31 and 0.37), the DMN (0.75 *vs* 0.4 and 0.4), and the dorsal dopamine pathway (0.53 *vs* 0.32 and 0.36), as shown in Fig. 2. Otherwise, cerebellar (0.35 *vs* 0.32 and 0.34), occipital (0.34 *vs* 0.37 and 0.35), limbic (0.29 *vs* 0.32 and 0.25), and temporal (0.13 *vs* 0.15 and 0.11) subnetworks displayed similar values of connectivity across groups (Supplementary T5).

**Fig. 3:**
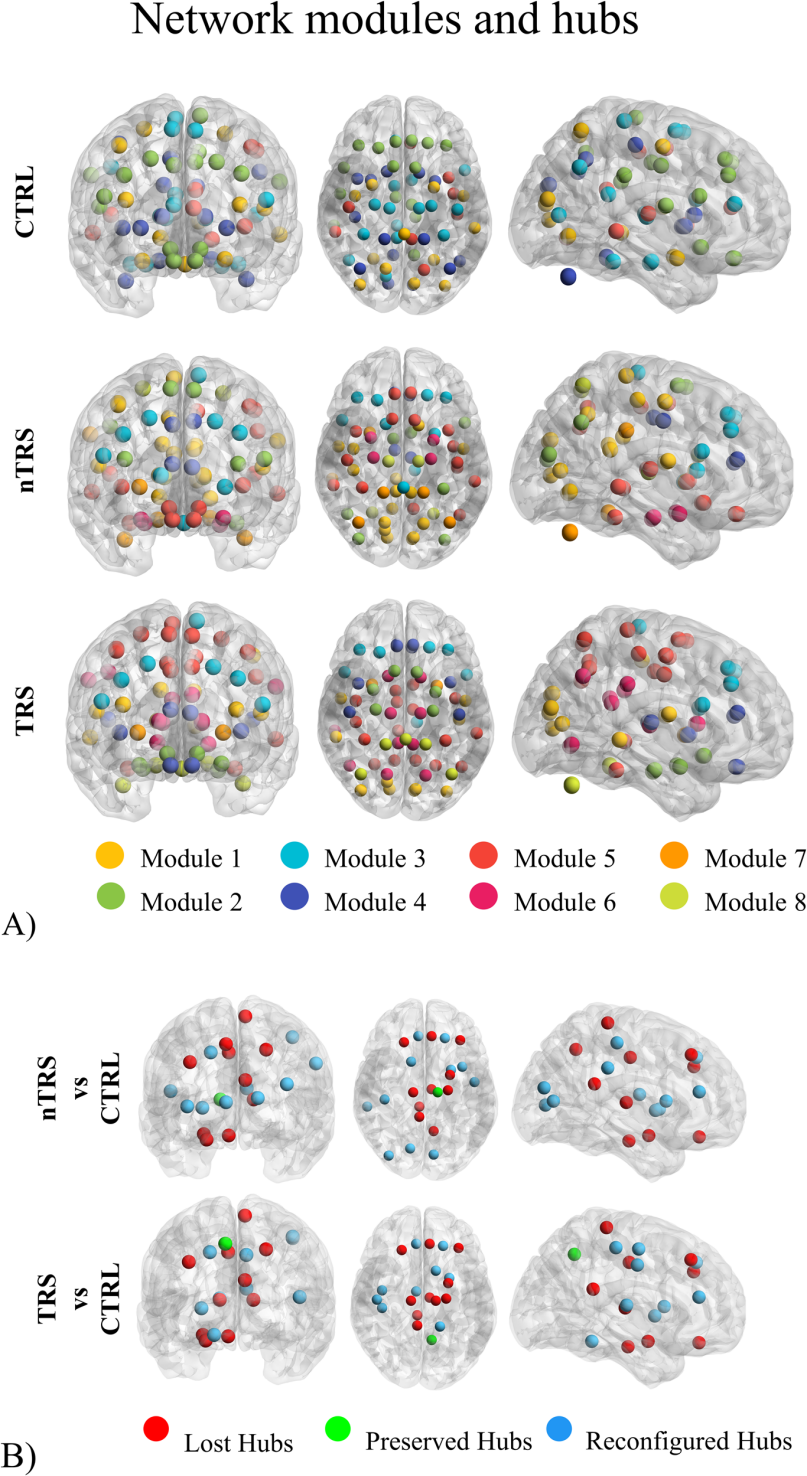
Network modules and hubs of CTRL, nTRS, and TRS groups. In **A**, nodes are represented with distinct colors indicating the modules to which they belong within each network. The Louvain method was employed to compute network communities for the CTRL, nTRS, and TRS groups. It was observed that individuals with schizophrenia exhibited a reduced level of modular organization compared to the CTRL group. More specifically, nodes within nTRS and TRS networks were distributed across eight communities, while CTRL nodes clustered into five modules. In **B**, differential hubs are present in both nTRS and TRS groups when compared to CTRL. Hubs are visualized using various colors to indicate their status: those present in the CTRL group but absent in patients (lost hubs in red), those present in both groups (preserved hubs in green), and those present in patients but not in CTRL (reconfigured hubs in blue). Eleven hubs (amygdala, mid and posterior part of the cingulate cortex, middle and superior frontal gyri, hippocampus, precuneus, rectus gyrus, thalamus, paracentral lobule) were identified in the CTRL group whereas the nTRS and TRS groups displayed twelve (anterior cingulate, calcarine fissure, caudate, superior frontal gyrus, insula, occipital lobe, postcentral gyrus, putamen, thalamus, Rolandic operculum, supramarginal gyrus) and ten hubs (anterior and mid part of the cingulate gyrus, caudate, cerebellum, superior frontal gyrus, Heschl’s gyrus, precuneus, precentral and postcentral gyri, putamen), respectively. Most of the affected hubs were comprised within the Default Mode Network and the dorsal dopamine pathway.

The CTRL group showed eleven hubs within five modules. In contrast, the nTRS and TRS groups displayed twelve and ten hubs, respectively, distributed across eight modules. The lower tendency of nodes to cluster together and the alteration in modular organization suggests a decreased efficiency and integration of schizophrenia networks. A global reconfiguration of hubs and modules in nTRS and TRS networks was identified and illustrated in Fig. 3. In the CTRL group, five out of the eleven hubs were part of the DMN and were lost in patients’ networks. Among the reconfigured hubs, such as hubs present in patients but not in CTRL networks, five out of the nine in the TRS group, as well as four out of the eleven in the nTRS group, were comprised within the dorsal dopamine pathway. Nodes’ participation coefficients, which were used for hub identification along with the Louvain method for modules’ detections, are represented in Supplementary T6.

Overall, these findings show altered connectivity in schizophrenia patients, particularly in the DMN and dorsal dopamine pathway, alongside changes in the modular organization of the metabolic networks and reconfigurations in the hub structure across the TRS and nTRS groups.

### Network comparison by statistical testing

Whole-brain differences in global strength were found between CTRL and patients’ networks (permutated *p*-value = 0.008 for CTRL *vs* nTRS and 0.001 for CTRL *vs* TRS), with a reduced connectivity in schizophrenia groups. No significant difference was found in global strength between nTRS and TRS (permutated *p*-value = 0.98).

**Fig. 4:**
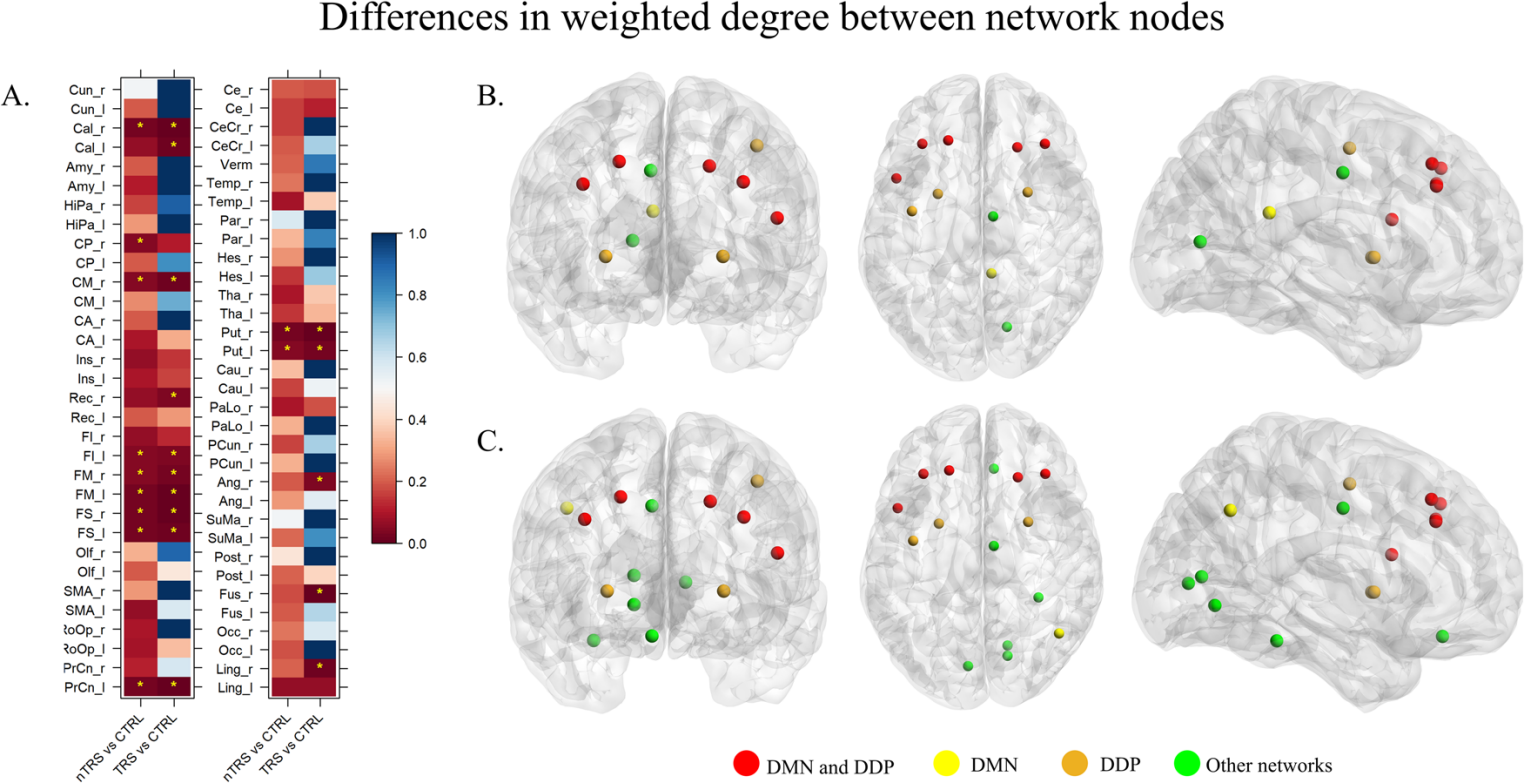
Differences in weighted degree between network nodes. Permutated *p*-values, corrected for multiple testing, are depicted on a color scale (**A**), ranging from 1 (blue) to 0 (red). Significant differences are denoted by stars (*). Nodes that significantly differed in strength between nTRS (**B**) and TRS (**C**) groups compared to CTRL are visually represented using different colors to indicate the network they belong to: yellow for nodes within the Default Mode Network (DMN), orange for nodes in the dorsal dopamine pathway (DDP), red for nodes included in both DMN and DDP, and green for nodes included in other networks. Affected nodes were the precentral gyrus, the superior, middle, and inferior frontal gyri, the putamen, the posterior and the mid part of the cingulate gyrus, the gyrus rectus, the angular, fusiform, and lingual gyrus, and the calcarine fissure.

Concerning centrality measures, both the nTRS and TRS networks exhibited significant differences from the CTRL group in terms of nodes’ weighted degree. The most noteworthy alterations were identified in the DMN and the dorsal dopamine pathway, as illustrated in Fig. 4. Specifically, permutation testing revealed eleven nodes with significantly different strength in the comparison between the nTRS and CTRL groups, with six nodes located in the DMN and eight in the dorsal dopamine pathway. In the comparison between the TRS and CTRL groups, fifteen nodes demonstrated a notable difference in weighted degree, with six nodes associated with the DMN and eight with the dorsal dopamine pathway. While values in weighted degree for each node within each group are provided in Supplementary T7, the heatmap of Fig. 4 and the Supplementary T8 provide a comprehensive overview of the statistical significance obtained through permutation testing. In the comparison between patients and CTRL networks, most affected nodes were the left precentral gyrus (*p*-value = 0.026 for nTRS; *p*-value = 0.006 for TRS), the left superior (*p*-value = 0.026 for nTRS; *p*-value = 0.013 for TRS), middle (*p*-value = 0.026 for nTRS; *p*-value = 0.006 for TRS), and inferior (*p*-value < 0.05 for nTRS; *p*-value = 0.04 for TRS) frontal gyri, the right superior (*p*-value = 0.026 for nTRS; *p*-value = 0.006 for TRS) and middle (*p*-value < 0.05 for nTRS; *p*-value = 0.026 for TRS) frontal gyri, the right (*p*-value = 0.029 for nTRS; *p*-value = 0.006 for TRS) and left (*p*-value < 0.05 for nTRS; *p*-value = 0.026 for TRS) putamen, the right mid part of the cingulate gyrus (*p*-value < 0.05 for nTRS; *p*-value = 0.02 for TRS), and the right calcarine fissure (*p*-value = 0.026 for nTRS; *p*-value = 0.006 for TRS). Compared to CTRL, other affected nodes were the right posterior part of the cingulate cortex (*p*-value < 0.05 for nTRS), the left calcarine fissure (*p*-value = 0.013 for TRS), the right gyrus rectus (*p*-value = 0.032 for TRS), angular gyrus (*p*-value = 0.039 for TRS), fusiform gyrus (*p*-value = 0.006 for TRS), and lingual gyrus (*p*-value = 0.013 for TRS).

**Fig. 5:**
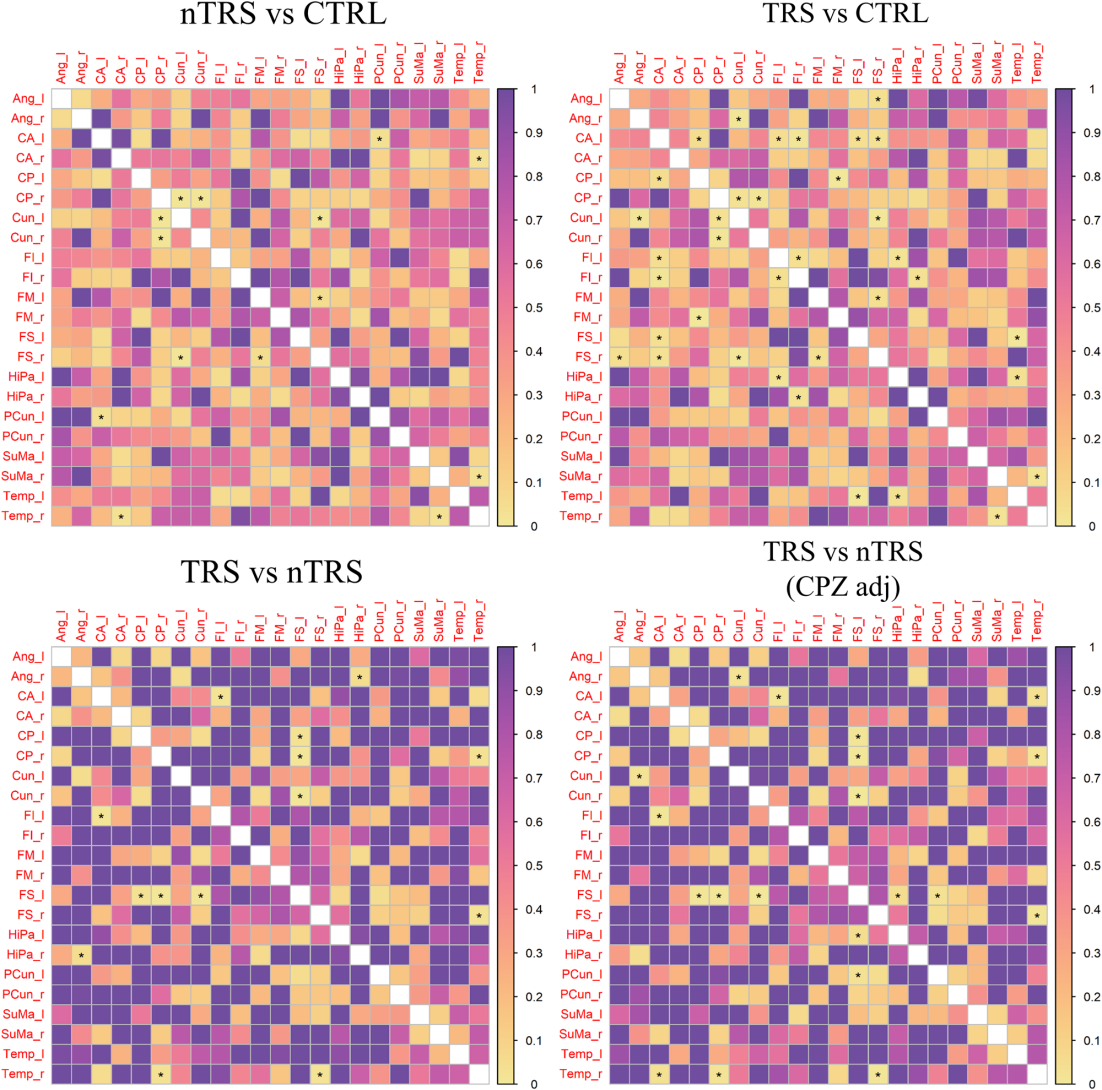
Differences in paired edges between group networks for the Default Mode Network. Statistical edge comparisons were computed for nTRS *vs* CTRL; TRS *vs* CTRL; nTRS *vs* TRS without and after adjustment for chlorpromazine equivalents. The results are presented as a matrix of *p*-values, with darker colors indicating values closer to 1 and lighter colors representing values closer to 0. Significant *p*-values are denoted by stars. Detected alterations include changes in hippocampal-frontal connectivity and correlations between the activity of the precuneus/cuneus and cingulate and frontal cortex (patients *vs* CTRL). In the comparison between nTRS and TRS, modifications in the connectivity of temporal and frontal areas, as well as cingulate regions, were identified. After correction for drug dosage, the correlation of the right hippocampus with the right angular gyrus disappeared, while differences in the connectivity of the superior frontal gyrus with the hippocampus and precuneus in the left hemisphere, as well as between the right temporal lobe and the left anterior cingulate, emerged.

**Fig. 6:**
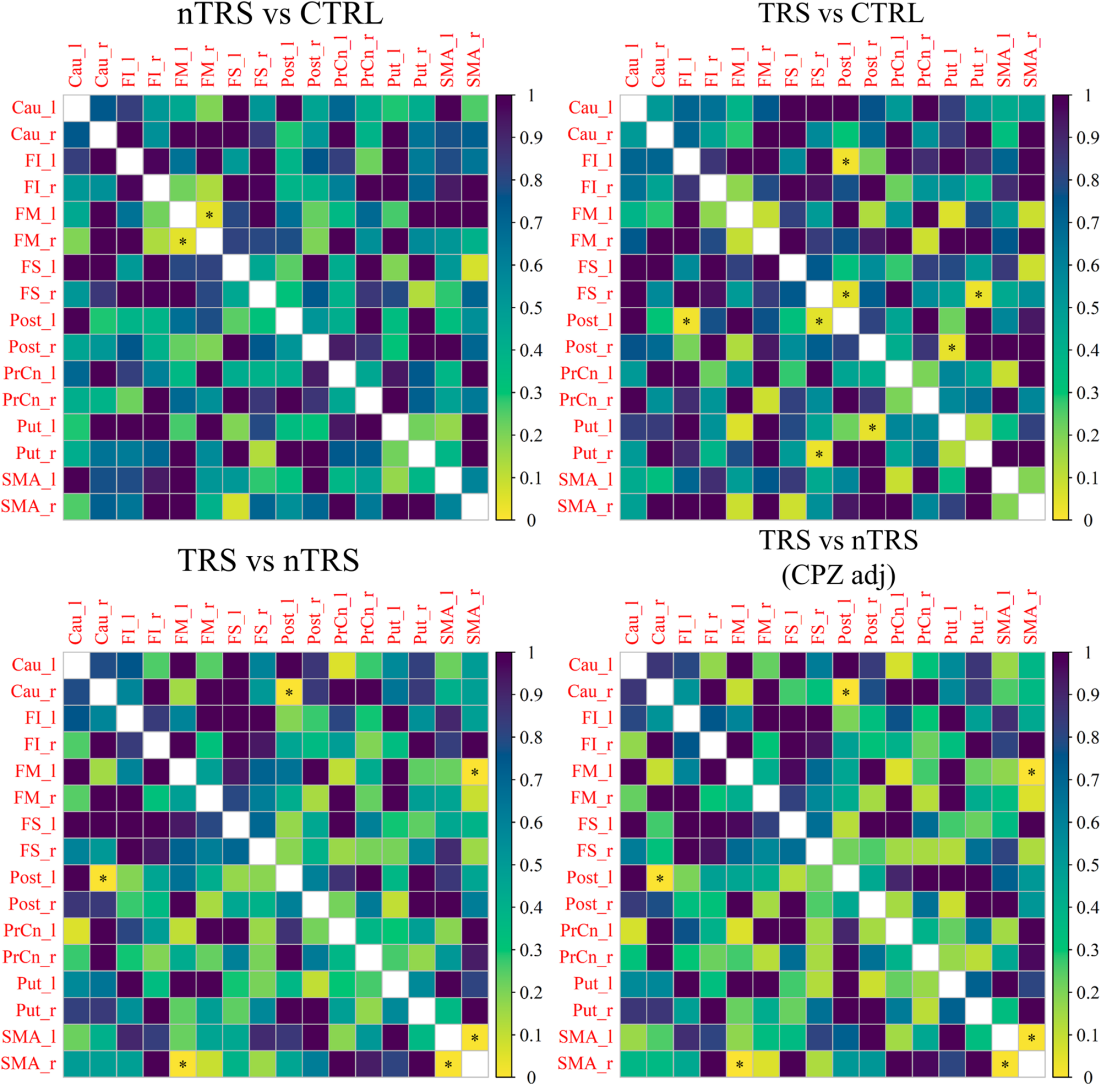
Differences in paired edges between group networks for the dorsal dopamine pathway. Statistical edge comparisons were computed for nTRS *vs* CTRL; TRS *vs* CTRL; nTRS *vs* TRS without and after adjustment for chlorpromazine equivalents. The results are presented as a matrix of *p*-values, with darker colors indicating values closer to 1 and lighter colors representing values closer to 0. Significant *p*-values are denoted by stars. Altered edges were observed in connections linking the supplementary motor area with other regions and in subcortical-cortical connectivity. No differences were observed by adjusting for chlorpromazine equivalents.

There were no notable variations in paired edges observed between the groups although the application of multiple testing correction significantly undermined the likelihood of obtaining significant results. Consequently, the comparisons were examined at the individual subnetwork level, including the DMN and dorsal dopamine pathway. Several connections significantly differed between group networks, especially within the DMN. By comparing nTRS and TRS networks with CTRL, significant alterations in the connections of the right posterior cingulate with the right (*p*-values = 0.015) and left (*p*-value = 0.034) cuneus were detected. The connectivity of the right superior frontal gyrus with the left cuneus (*p*-values = 0.012 and 0.006, respectively for nTRS and TRS *vs* CTRL) and the left middle frontal gyrus (*p*-values = 0.049 and 0.039) were notably impaired. Disrupted connections were further observed between the right temporal region and the right supramarginal gyrus (*p*-value = 0.029 and 0.024) and, for the nTRS group only, the right anterior cingulate (*p*-value = 0.028). The correlation between the left precuneus and the left anterior cingulate was significantly altered in the comparison of nTRS *vs* CTRL (*p*-value = 0.037). Significant alterations in the connection between the hippocampus and the inferior frontal gyrus within the left (*p*-value = 0.022) and right (*p*-value = 0.028) hemispheres were detected by comparing TRS and CTRL. The inferior frontal gyrus also exhibited dysconnectivity between its right and left hemispheric locations (*p*-value = 0.039). Disrupted connections were identified between the left temporal region and the left superior frontal gyrus (*p*-value = 0.005) and the left hippocampus (*p*-value = 0.036). The left anterior cingulate showed alterations in functional connectivity with several brain regions, including the left and right inferior and superior frontal gyri. Altered subcortical-cortical connectivity was identified in TRS patients compared to CTRL, especially concerning the edges linking the right and left putamen with the right superior frontal gyrus (*p*-value = 0.034) and postcentral gyrus (*p*-value = 0.04). Of interest, impairments in the connectivity of the right temporal region with the right posterior cingulate (*p*-value = 0.022) and superior frontal gyrus (*p*-value = 0.001) were detected in the comparison between nTRS and TRS groups. Other differences were identified in the correlation between cingulate and frontal regions as well as in the connectivity of the right hippocampus with the right angular gyrus (not detected after adjustment for chlorpromazine equivalents). The correction for medication dosage unveiled further differences in the connectivity of the superior frontal gyrus with the hippocampus (*p*-value = 0.049) and precuneus (*p*-value = 0.035) in the left hemisphere, as well as between the right temporal lobe and the left anterior cingulate (*p*-value = 0.035). Altered functional connectivity was observed regarding the supplementary motor area and in the connection between the right caudate with the left postcentral gyrus (*p*-value = 0.005). A comprehensive report of differential paired connections is available in the Supplementary T9–16, as well as in the matrices of Figs. 5 and 6. Brain plots relative to edge differences are provided in Supplementary F8, 9. Effects of age, sex, years of education, duration of illness, and age of onset were reported in Supplementary F10–14.

In summary, significant differences in global connectivity strength were found between the CTRL and schizophrenia groups, with no notable differences between nTRS and TRS. Alterations in weighted degree were observed, particularly affecting nodes within the DMN and dorsal dopamine pathway. Further subnetwork-level comparisons revealed additional connectivity changes, especially between cortical and subcortical regions, as well as between frontal, temporal, and cingulate areas, which distinguish TRS from nTRS.

### Association between metabolic signals and treatment resistance based on machine-learning

**Fig. 7:**
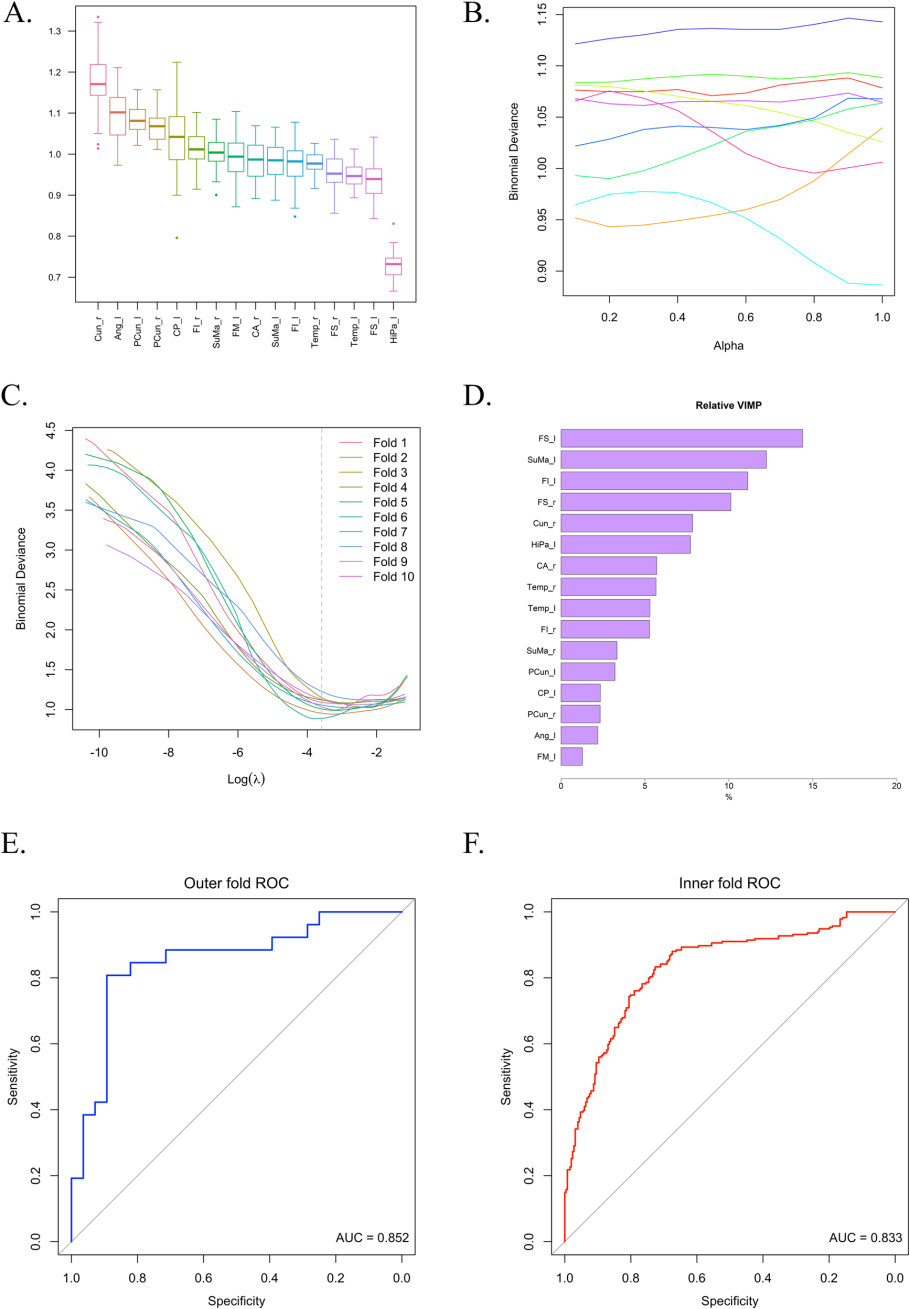
Results from nested cross-validation using data of DMN regions. Boxplots of model variables are depicted to show the distribution of glucose signals along the selected VOIs (**A**). It is shown how deviance is affected by alpha (**B**) and lambda (**C**). In **D**, a scale of variable importance was provided through coefficients estimated by the model. The coefficients were normalized to obtain the relative importance of each variable. This approach provides an overview of the variables that contribute most to the model’s performance. ROC curves from both outer (**E**) and inner (**F**) cross-validation are plotted.

To estimate the associations between the metabolic signals of VOIs and treatment responsiveness or resistance in schizophrenia patients, a logistic model was computed using a nested cross-validation approach. The assessment of the binary outcome was based on the ^18^F-FDG signal, considering the whole pool of VOIs, regions of the DMN, or areas within the dorsal dopamine pathways. The results indicated higher accuracy when utilizing DMN data (accuracy = 0.83), followed by the dorsal dopamine pathway (accuracy = 0.81), and then the entire dataset (accuracy = 0.75). The AUC was 0.85 by selecting brain regions comprised in the DMN (95%C.I. = 0.74, 0.96), 0.83 with the dorsal dopamine pathway (95%C.I. = 0.71, 0.95), and 0.81 by utilizing the whole dataset (95%C.I. = 0.69, 0.92). The Brier score was 0.15 by selecting data from DMN, 0.17 with the dorsal dopamine pathway, and 0.18 when the whole dataset was used. Models’ coefficients, ROC curves from both outer and inner folds, deviance due to alpha and lambda hyperparameters are represented in Fig. 7 and Supplementary F15, 16.

## Discussion

To the best of our knowledge, the present study marks the first attempt to unveil alterations in schizophrenia responsive and resistant patients based on ^18^F-FDG metabolic connectomics.

### Graph-theory driven analyses

By a qualitative approach, our metabolic brain connectivity analysis revealed altered global properties, nodes’ centrality, and network organization in schizophrenia. Patients’ networks exhibited a global hypoconnectivity pattern, reduced integration, and efficiency in communication, primarily in frontal regions, DMN, and dorsal dopamine pathway. The graph analysis showed a lower tendency of nodes to cluster together and a decreased level of modular organization in schizophrenia groups. These connectivity disruptions were associated with changes in centrality measures of network nodes and a reconfiguration of relevant hubs.

These findings align with previous research on schizophrenia. Specifically, a meta-analysis of resting-state functional connectivity showed that chronic schizophrenia was characterized by hypoconnectivity within large-scale brain networks, particularly the DMN^8^. Similarly, reduced global strength, along with lower clustering coefficient, higher count of smaller communities, and reconfiguration of hubs comprised in the DMN were found in chronic schizophrenia^64^. Thus, the hypoconnectivity pattern and the altered modular organization observed in the present study could represent features specific for chronic schizophrenia, which may depend on long-term antipsychotic medication^65^. However, it has been demonstrated that the antipsychotic dose does not correlate with brain metabolic activity in both treatment-responsive and treatment-resistant patients^29^, as well as in first-episode psychosis patients^66^. The latter suggests that the alterations observed may indeed represent biological correlates of the chronic course of the disease rather than being an artifact of antipsychotic medication.

### Network-level differences between patients and controls

In this study, direct statistical comparisons of metabolic connectivity networks between groups revealed significant differences in the weighted degree of nodes and paired edges across several brain regions, including the superior, middle, and inferior frontal gyri, precentral gyrus, calcarine fissure, and cingulate regions. These findings support our working hypothesis that schizophrenia is associated with widespread dysconnectivity across key brain networks.

Frontal lobe dysconnectivity has been consistently identified as a core feature of schizophrenia, both in acute and chronic forms of the disorder^67–69^. Our findings align with previous research showing altered connectivity between the superior frontal gyrus and cuneus in schizophrenia patients^70^. Additionally, global functional connectivity of the left superior frontal gyrus was found to differentiate the patients or siblings from controls, further supporting the idea of frontal lobe dysfunction as a potential neurobiological endophenotype^67^. Other studies have reported impaired connections between frontal regions^71,72^, particularly between the middle and superior frontal gyri, which we also observed^72^. Abnormalities in the inferior frontal gyrus have been linked to clinical symptoms, such as delusions/blunted affect in first-episode psychosis, and semantic processing deficits in chronic schizophrenia^68,72^.

Our analysis of the left precentral gyrus also revealed significant disruptions in schizophrenia patients, consistent with previous studies using fMRI, which identified this region as part of the altered “social brain” circuitry^73–76^. Reduced activation in the left precentral gyrus during facial emotion processing has been documented in schizophrenia patients, particularly when compared to non-psychotic siblings and healthy controls^76^.

Regarding the calcarine fissure, we identified dysconnectivity in both the right and left hemispheres. This aligns with a meta-analysis showing decreased regional homogeneity in the bilateral calcarine fissure of schizophrenia patients^75^, as well as functional decoupling between the calcarine fissure and the cuneus, both in patients and individuals at ultra-high risk for psychosis^77–79^. Notably, first-episode schizophrenia patients showed increased activity in the calcarine fissure following antipsychotic treatment, suggesting that this region may be sensitive to therapeutic interventions^80^.

Disruptions in cingulate connectivity were also evident in our analysis. These findings are consistent with previous studies reporting impaired connectivity between the posterior cingulate/precuneus and the anterior cingulate cortex^81,82^, the cuneus^82^, and frontal areas^82^, as well as between the anterior cingulate region and the right middle temporal gyrus^74^. These regions are critical components of the DMN, and alterations in their connectivity have been associated with the severity of psychotic symptoms, including hallucinations, delusions, thought disturbances, and negative symptoms^83^. Further, decreased striatal–cortical DMN connectivity has been associated with an elevated risk of developing psychotic symptoms, highlighting the potential role of DMN disruptions in the pathophysiology of schizophrenia^84^.

### Different neurobiological correlates for treatment-resistant schizophrenia

In the present study, significant differences were observed in the metabolic networks of individuals affected by TRS compared to CTRL and nTRS, particularly in the DMN, frontotemporal, and subcortical-cortical connectivity. These findings support our initial hypothesis that TRS patients exhibit a distinct pattern of dysconnectivity compared to nTRS and controls.

Permutation testing highlighted a different reorganization of DMN in non-responders, with altered connectivity involving the frontal, cingulate, and temporal regions. Further, TRS patients exhibited hippocampal functional decoupling from the angular gyrus and inferior frontal gyrus. By controlling for the effects of medication dosage, alterations in the functional connectivity of the anterior cingulate cortex, along with the frontotemporal network, emerged as potential contributors to TRS. These results are consistent with previous research, which highlights frontotemporal dysconnectivity as a neurobiological underpinning of TRS^85–88^. The observed dysfunction within a bilateral fronto-temporo-parietal network, including the anterior and posterior cingulate, reinforces the hypothesis that TRS patients may exhibit a distinct pattern of dysconnectivity, which may contribute to the persistence of symptoms unresponsive to antipsychotic treatments^85,89,90^. Moreover, our machine learning analysis confirmed the importance of metabolic signals from key regions, such as the anterior and posterior cingulate, superior and inferior frontal gyri, and hippocampus, in distinguishing TRS from nTRS patients. Of interest, neurostimulation techniques in TRS patients have revealed an association between clinical improvements and the functional connectivity among temporal areas, frontal gyri, angular gyrus, and hippocampus, suggesting these regions may be therapeutic targets for intervention^85,91–93^.

The observed disruptions in anterior cingulate connectivity are particularly notable given the well-established role of this region in TRS pathophysiology. Specifically, previous studies have shown elevated glutamate levels in the anterior cingulate cortex of TRS patients, which have been linked to cognitive deficits and reduced cortical thickness^94–102^. Of interest, ^18^F-FDG-PET imaging has been suggested as a proxy for assessing glutamatergic neurotransmission^103,104^. This proposal is grounded in the concept that glutamate is produced in neurons from glucose-derived tricarboxylic acid cycle intermediates and branched-chain amino acids^104^. Additionally, glucose-derived energy is necessary for uptake and release of glutamate^104^.

Our analysis also uncovered disruptions in connectivity involving the putamen and frontal regions when comparing TRS patients to CTRL. These findings are consistent with previous studies that have considered dysconnectivity within the striatal-cortical network, particularly involving the putamen and caudate, as a putative biological indicator of psychosis and predictors of treatment responsiveness in schizophrenia^105,106^.

We observed further alterations in the TRS group, concerning connectivity in the right angular and fusiform gyrus, and within the dorsal dopamine pathway, including the left postcentral gyrus and right caudate. The connectivity changes in the postcentral, angular, and fusiform gyrus could serve as potential discriminators between TRS and nTRS, as suggested by previous neuroimaging research^107^. Aberrant connections between the postcentral gyrus and right dorsal caudate were identified in individuals experiencing a breakthrough psychosis episode while on antipsychotic maintenance medication compared to those who were antipsychotic-free at the time of relapse, indicating that these regions may also play a role in the underlying mechanisms of treatment resistance^108^. In this context, a recent study employed a functional MRI-based sliding window analysis to calculate weighted permutation entropy, revealing reduced complexity in the caudate at baseline in schizophrenia patients compared to healthy controls. Following treatment, the complexity of the left caudate increased, indicating a potential normalization of activity patterns after antipsychotic intervention^109^.

Interestingly, after correcting for both duration of illness and age of onset, we observed a significant alteration in connectivity between the cuneus and the posterior cingulate. This finding is noteworthy, as it bears some resemblance to the cingulate island sign, which has been described in the context of dementia with Lewy bodies and may be related to cognitive alterations^110^.

### Limitations

The study has limitations that warrant consideration. Firstly, the relatively small sample size compared to the high number of analyzed brain regions may introduce bias, as well as unequal sample size between patients and controls. To address the high-dimensional data problem, AAL-merged atlas to define VOIs, GGMs with atan regularization for unbiased estimations, permutation testing, and nested cross-validation were employed. Permutation testing was also employed to better deal with unbalanced sample sizes across groups^55^. It is important to note that previous studies investigating metabolic connectivity and those utilizing machine learning analyses often employed similar sample sizes^23,111^.

Secondly, the recruited patients were under antipsychotic treatment, making it challenging to distinguish medication effects from those associated with the disorder. To address this methodological issue and balance drug doses across groups, we employed a stratification strategy during nested cross-validation and considered chlorpromazine equivalents as a covariate when calculating edges in permutation-based comparisons, minimizing the risk of medication-related biases in our results.

To mitigate Type I errors, we have employed permutation testing and adjustment of *p*-values for multiple comparisons, which help control for false discovery rates in our statistical comparisons. While the use of GGMs with atan penalty allows for flexible modeling of brain region relationships and may help reduce Type II errors, we acknowledge that larger sample sizes would enhance the reliability of our findings.

## Conclusion

This study tackles a novel approach to metabolic connectivity analysis in psychiatric disorders, particularly schizophrenia. Using a methodology that integrates graph theory, network comparison, and machine learning, we identified significant alterations in global brain network properties, nodal degrees, and interregional connectivity. These findings underscore the potential of ^18^F-FDG-PET-based metabolic connectivity to enhance understanding of schizophrenia and to distinguish treatment-responsive from treatment-resistant patients. Our results align with findings from previous studies and suggest high accuracy for metabolic signals in characterizing TRS. Future studies focusing specifically on ultra-treatment-resistant patients could provide valuable insights into more severe forms of treatment resistance.

### Supplementary Information

Supplementary Information comprise brain regions abbreviations, demographic and clinical characteristics, correlation matrices, network metrics, and permutation-based comparisons.

## Supplementary information

Supplementary Information

## Data Availability

Data are available upon request.

## References

[1] Correll, C.U. & Howes, O.D. Treatment-Resistant Schizophrenia: Definition, Predictors, and Therapy Options. *J. Clin. Psychiatry* **82**, MY20096AH1C (2021). 10.4088/JCP.MY20096AH1C 34496461

[2] Howes, O. D., McCutcheon, R., Agid, O., de Bartolomeis, A., van Beveren, N. J. & Birnbaum, M. L. et al. Treatment-Resistant Schizophrenia: Treatment Response and Resistance in Psychosis (TRRIP) Working Group Consensus Guidelines on Diagnosis and Terminology. *Am. J. Psychiatry* **174**, 216–229 (2017). 27919182 10.1176/appi.ajp.2016.16050503 PMC6231547

[3] Iasevoli, F., Giordano, S., Balletta, R., Latte, G., Formato, M. V. & Prinzivalli, E. et al. Treatment resistant schizophrenia is associated with the worst community functioning among severely-ill highly-disabling psychiatric conditions and is the most relevant predictor of poorer achievements in functional milestones. *Prog. Neuro Psychopharmacol. Biol. psychiatry* **65**, 34–48 (2016). 10.1016/j.pnpbp.2015.08.010 26320028

[4] Howes, O. D. & Onwordi, E. C. The synaptic hypothesis of schizophrenia version III: a master mechanism. *Mol. Psychiatry* **28**, 1843–1856 (2023). 37041418 10.1038/s41380-023-02043-w PMC10575788

[5] Friston, K., Brown, H. R., Siemerkus, J. & Stephan, K. E. The dysconnection hypothesis (2016). *Schizophrenia Res.* **176**, 83–94 (2016). 10.1016/j.schres.2016.07.014 PMC5147460 27450778

[6] Obi-Nagata, K., Temma, Y. & Hayashi-Takagi, A. Synaptic functions and their disruption in schizophrenia: From clinical evidence to synaptic optogenetics in an animal model. *Proc. Jpn. Acad. Ser. B Phys. Biol. Sci.* **95**, 179–197 (2019). 31080187 10.2183/pjab.95.014 PMC6742729

[7] Chung, D. W., Geramita, M. A. & Lewis, D. A. Synaptic Variability and Cortical Gamma Oscillation Power in Schizophrenia. *Am. J. Psychiatry* **179**, 277––287 (2022). 35360919 10.1176/appi.ajp.2021.21080798 PMC9580070

[8] Dong, D., Wang, Y., Chang, X., Luo, C. & Yao, D. Dysfunction of Large-Scale Brain Networks in Schizophrenia: A Meta-analysis of Resting-State Functional Connectivity. *Schizophrenia Bull.* **44**, 168–181 (2018). 10.1093/schbul/sbx034 PMC5767956 28338943

[9] Seitz-Holland, J., Wojcik, J. D., Cetin-Karayumak, S., Lyall, A. E., Pasternak, O. & Rathi, Y. et al. Cognitive deficits, clinical variables, and white matter microstructure in schizophrenia: a multisite harmonization study. *Mol. Psychiatry* **27**, 3719–3730 (2022). 35982257 10.1038/s41380-022-01731-3 PMC10538303

[10] Lei, D., Li, W., Tallman, M. J., Strakowski, S. M., DelBello, M. P. & Rodrigo Patino, L. et al. Changes in the structural brain connectome over the course of a nonrandomized clinical trial for acute mania. *Neuropsychopharmacology* **47**, 1961–1968 (2022). 35585125 10.1038/s41386-022-01328-y PMC9485114

[11] Del Fabro, L., Schmidt, A., Fortea, L., Delvecchio, G., D’Agostino, A. & Radua, J. et al. Functional brain network dysfunctions in subjects at high-risk for psychosis: A meta-analysis of resting-state functional connectivity. *Neurosci. Biobehav. Rev.* **128**, 90–101 (2021). 34119524 10.1016/j.neubiorev.2021.06.020

[12] Tsugawa, S., Honda, S., Noda, Y., Wannan, C., Zalesky, A. & Tarumi, R. et al. Associations Between Structural Covariance Network and Antipsychotic Treatment Response in Schizophrenia. *Schizophrenia Bull.* **50**, 382–392 (2023). 10.1093/schbul/sbad160 PMC10919786 37978044

[13] Sone, D., Young, A., Shinagawa, S., Tsugawa, S., Iwata, Y. & Tarumi, R. et al. Disease Progression Patterns of Brain Morphology in Schizophrenia: More Progressed Stages in Treatment Resistance. *Schizophrenia Bull.* **50**, 393–402 (2023). 10.1093/schbul/sbad164 PMC10919766 38007605

[14] Kim, E., Howes, O. D., Veronese, M., Beck, K., Seo, S. & Park, J. W. et al. Presynaptic Dopamine Capacity in Patients with Treatment-Resistant Schizophrenia Taking Clozapine: An [(18)F]DOPA PET Study. *Neuropsychopharmacology* **42**, 941–950 (2017). 27857125 10.1038/npp.2016.258 PMC5312074

[15] Demjaha, A., Murray, R. M., McGuire, P. K., Kapur, S. & Howes, O. D. Dopamine synthesis capacity in patients with treatment-resistant schizophrenia. *Am. J. Psychiatry* **169**, 1203–1210 (2012). 23034655 10.1176/appi.ajp.2012.12010144

[16] Shin, S., Jung, W. H., McCutcheon, R., Veronese, M., Beck, K. & Lee, J. S. et al. The Relationship Between Frontostriatal Connectivity and Striatal Dopamine Function in Schizophrenia: An 18F-DOPA PET and Diffusion Tensor Imaging Study in Treatment Responsive and Resistant Patients. *Psychiatry Investig.* **19**, 570–579 (2022). 35903059 10.30773/pi.2022.0033 PMC9334810

[17] Bertolino, A., Breier, A., Callicott, J. H., Adler, C., Mattay, V. S. & Shapiro, M. et al. The Relationship between Dorsolateral Prefrontal Neuronal N-Acetylaspartate and Evoked Release of Striatal Dopamine in Schizophrenia. *Neuropsychopharmacology* **22**, 125–132 (2000). 10649825 10.1016/S0893-133X(99)00096-2

[18] Breier, A., Su, T. P., Saunders, R., Carson, R. E., Kolachana, B. S. & de Bartolomeis, A. et al. Schizophrenia is associated with elevated amphetamine-induced synaptic dopamine concentrations: evidence from a novel positron emission tomography method. *Proc. Natl Acad. Sci. USA* **94**, 2569–2574 (1997). 9122236 10.1073/pnas.94.6.2569 PMC20129

[19] Rubio, J.M., Lencz, T., Cao, H., Kraguljac, N., Dhamala, E., Homan, P. et al. Replication of a neuroimaging biomarker for striatal dysfunction in psychosis. *Mol. Psychiatry* **29**, 929–938 (2024). 10.1038/s41380-023-02381-9 PMC11176002 38177349

[20] Sala, A., Lizarraga, A., Caminiti, S. P., Calhoun, V. D., Eickhoff, S. B. & Habeck, C. et al. Brain connectomics: time for a molecular imaging perspective? *Trends Cogn. Sci.* **27**, 353–366 (2023). 36621368 10.1016/j.tics.2022.11.015 PMC10432882

[21] Archila-Meléndez, M. E., Sorg, C. & Preibisch, C. Modeling the impact of neurovascular coupling impairments on BOLD-based functional connectivity at rest. *NeuroImage* **218**, 116871 (2020). 32335261 10.1016/j.neuroimage.2020.116871

[22] Yakushev, I., Drzezga, A., Habeck, C. Metabolic connectivity: methods and applications. *Curr. Opin. Neurol.* **30**, 677–685 (2017). 10.1097/WCO.0000000000000494 28914733

[23] Caminiti, S. P., Tettamanti, M., Sala, A., Presotto, L., Iannaccone, S., Cappa, S. F. et al. Metabolic connectomics targeting brain pathology in dementia with Lewy bodies. *J. Cereb. Blood Flow. Metab.* **37**, 1311–1325 (2017). 10.1177/0271678X16654497 PMC5453453 27306756

[24] Carli, G., Caminiti, S. P., Sala, A., Galbiati, A., Pilotto, A. & Ferini-Strambi, L. et al. Impaired metabolic brain networks associated with neurotransmission systems in the α-synuclein spectrum. *Parkinsonism Relat. Disord.* **81**, 113–122 (2020). 33120072 10.1016/j.parkreldis.2020.10.036

[25] Malpetti, M., Carli, G., Sala, A., Cerami, C., Marcone, A. & Iannaccone, S. et al. Variant-specific vulnerability in metabolic connectivity and resting-state networks in behavioural variant of frontotemporal dementia. *Cortex* **120**, 483–497 (2019). 31493687 10.1016/j.cortex.2019.07.018

[26] Sala, A., Caminiti, S. P., Presotto, L., Premi, E., Pilotto, A. & Turrone, R. et al. Altered brain metabolic connectivity at multiscale level in early Parkinson’s disease. *Sci. Rep.* **7**, 4256 (2017). 28652595 10.1038/s41598-017-04102-z PMC5484707

[27] Huang, S., Li, J., Sun, L., Ye, J., Fleisher, A. & Wu, T. et al. Learning brain connectivity of Alzheimer’s disease by sparse inverse covariance estimation. *NeuroImage* **50**, 935–949 (2010). 20079441 10.1016/j.neuroimage.2009.12.120 PMC3068623

[28] Titov, D., Diehl-Schmid, J., Shi, K., Perneczky, R., Zou, N. & Grimmer, T. et al. Metabolic connectivity for differential diagnosis of dementing disorders. *J. Cereb. Blood Flow. Metab.* **37**, 252–262 (2017). 26721391 10.1177/0271678X15622465 PMC5363743

[29] Iasevoli, F., D’Ambrosio, L., Ciccarelli, M., Barone, A., Gaudieri, V. & Cocozza, S. et al. Altered Patterns of Brain Glucose Metabolism Involve More Extensive and Discrete Cortical Areas in Treatment-resistant Schizophrenia Patients Compared to Responder Patients and Controls: Results From a Head-to-Head 2-[18F]-FDG-PET Study. *Schizophrenia Bull.* **49**, 474–485 (2022). 10.1093/schbul/sbac147 PMC10016407 36268829

[30] Townsend, L., Pillinger, T., Selvaggi, P., Veronese, M., Turkheimer, F. & Howes, O. Brain glucose metabolism in schizophrenia: a systematic review and meta-analysis of (18)FDG-PET studies in schizophrenia. *Psychological Med.* **53**, 4880–4897 (2023). 10.1017/S003329172200174X PMC10476075 35730361

[31] de Bartolomeis, A., De Simone, G., De Prisco, M., Barone, A., Napoli, R., Beguinot, F. et al. Insulin effects on core neurotransmitter pathways involved in schizophrenia neurobiology: a meta-analysis of preclinical studies. Implications for the treatment. *Mol. Psychiatry* **28**, 2811–2825 (2023). 10.1038/s41380-023-02065-4 PMC10615753 37085712

[32] Tang, S. X., Oliver, L. D., Hänsel, K., DeRosse, P., John, M. & Khairullah, A. et al. Metabolic disturbances, hemoglobin A1c, and social cognition impairment in Schizophrenia spectrum disorders. *Transl. Psychiatry* **12**, 233 (2022). 35668078 10.1038/s41398-022-02002-z PMC9170776

[33] Desco, M., Gispert, J. D., Reig, S., Sanz, J., Pascau, J. & Sarramea, F. et al. Cerebral metabolic patterns in chronic and recent-onset schizophrenia. *Psychiatry Res.* **122**, 125–135 (2003). 12714176 10.1016/s0925-4927(02)00124-5

[34] Molina, V., Sanz, J., Sarramea, F. & Palomo, T. Marked hypofrontality in clozapine-responsive patients. *Pharmacopsychiatry* **40**, 157–162 (2007). 17694479 10.1055/s-2007-984399

[35] Lee, J., Xue, X., Au, E., McIntyre, W. B., Asgariroozbehani, R. & Panganiban, K. et al. Glucose dysregulation in antipsychotic-naive first-episode psychosis: in silico exploration of gene expression signatures. *Transl. Psychiatry* **14**, 19 (2024). 38199991 10.1038/s41398-023-02716-8 PMC10781725

[36] Mazza, E., Calesella, F., Paolini, M., di Pasquasio, C., Poletti, S. & Lorenzi, C. et al. Insulin resistance disrupts white matter microstructure and amplitude of functional spontaneous activity in bipolar disorder. *Bipolar Disord.* **25**, 32–42 (2023). 36377438 10.1111/bdi.13270

[37] Guedj, E., Varrone, A., Boellaard, R., Albert, N. L., Barthel, H. & van Berckel, B. et al. EANM procedure guidelines for brain PET imaging using [(18)F]FDG, version 3. *Eur. J. Nucl. Med. Mol. imaging* **49**, 632–651 (2022). 34882261 10.1007/s00259-021-05603-w PMC8803744

[38] Kuang, L., Jia, J., Zhao, D., Xiong, F., Han, X., Wang, Y. et al. Default Mode Network Analysis of APOE Genotype in Cognitively Unimpaired Subjects Based on Persistent Homology. *Front. Aging Neurosci.* **12**, 188 (2020). 10.3389/fnagi.2020.00188 PMC7358981 32733231

[39] Bernal, V., Soancatl-Aguilar, V., Bulthuis, J., Guryev, V., Horvatovich, P. & Grzegorczyk, M. GeneNetTools: tests for Gaussian graphical models with shrinkage. *Bioinformatics* **38**, 5049–5054 (2022). 36179082 10.1093/bioinformatics/btac657 PMC9665865

[40] Wang, Y. & Zhu, L. Variable Selection and Parameter Estimation with the Atan Regularization Method. *J. Probab. Stat.* **2016**, 6495417 (2016).

[41] Williams, D. R. GGMncv: Nonconvex Penalized Gaussian Graphical Models in R (2020). *PsyArXiv.*10.31234/osf.io/6jz5m.

[42] Williams, D. R. Beyond Lasso: A Survey of Nonconvex Regularization in Gaussian Graphical Models. *PsyArXiv* (2020). 10.31234/osf.io/ad57p.

[43] Watson. brainGraph: Graph Theory Analysis of Brain MRI Data (2024). Retrieved from CRAN. 10.32614/CRAN.package.brainGraph.

[44] Sporns, O. Structure and function of complex brain networks. *Dialogues Clin. Neurosci.* **15**, 247–262 (2013). 24174898 10.31887/DCNS.2013.15.3/osporns PMC3811098

[45] Sporns, O. & Zwi, J. D. The small world of the cerebral cortex. *Neuroinformatics* **2**, 145–162 (2004). 15319512 10.1385/NI:2:2:145

[46] Power, J. D., Schlaggar, B. L., Lessov-Schlaggar, C. N. & Petersen, S. E. Evidence for hubs in human functional brain networks. *Neuron* **79**, 798–813 (2013). 23972601 10.1016/j.neuron.2013.07.035 PMC3838673

[47] Csárdi, G., Nepusz, T., Müller, K., Horvát, S., Traag, V., Zanini, F., & Noom, D. *igraph for R: R interface of the igraph library for graph theory and network analysis (v1.5.0.1)* (Zenodo, 2023).

[48] Csardi, G. & Nepusz, T. *The Igraph Software Package for Complex Network Research* (InterJournal, 2005).

[49] Arratia, A. & Renedo Mirambell, M. Clustering assessment in weighted networks. *PeerJ Computer Sci.* **7**, e600 (2021). 10.7717/peerj-cs.600 PMC8237321 34239979

[50] RStudio: Integrated Development for R. RStudio P, Boston, MA, http://www.rstudio.com/ (2020).

[51] Xia, M., Wang, J. & He, Y. BrainNet Viewer: a network visualization tool for human brain connectomics. *PloS one* **8**, e68910 (2013). 23861951 10.1371/journal.pone.0068910 PMC3701683

[52] Christensen, W. F. & Zabriskie, B. N. When Your Permutation Test is Doomed to Fail. *Am. Statistician* **76**, 53–63 (2022).

[53] Leem, S., Huh, I. & Park, T. Enhanced Permutation Tests via Multiple Pruning. *Front. Genet.* **11**, 509 (2020). 10.3389/fgene.2020.00509 PMC7330123 32670346

[54] Veronese, M., Moro, L., Arcolin, M., Dipasquale, O., Rizzo, G. & Expert, P. et al. Covariance statistics and network analysis of brain PET imaging studies. *Sci. Rep.* **9**, 2496 (2019). 30792460 10.1038/s41598-019-39005-8 PMC6385265

[55] Nichols, T. E. & Holmes, A. P. Nonparametric permutation tests for functional neuroimaging: a primer with examples. *Hum. Brain Mapp.* **15**, 1–25 (2002). 11747097 10.1002/hbm.1058 PMC6871862

[56] Barone, A., De Simone, G., Ciccarelli, M., Buonaguro, E. F., Tomasetti, C. & Eramo, A. et al. A Postsynaptic Density Immediate Early Gene-Based Connectome Analysis of Acute NMDAR Blockade and Reversal Effect of Antipsychotic Administration. *Int J. Mol. Sci.* **24**, 4372 (2023). 36901803 10.3390/ijms24054372 PMC10002165

[57] van Borkulo, C.D., van Bork, R., Boschloo, L., Kossakowski, J.J., Tio, P., Schoevers, R.A. et al. Comparing network structures on three aspects: A permutation test. *Psychological Methods* **28**, 1273–1285 (2023). 10.1037/met0000476 35404628

[58] Wei, T., Simko, V., Levy, M., Xie, Y., Jin, Y., Zemla, J.J. R. pv. corrplot: Visualization of a correlation matrix. 230 (2013). Retrieved from CRAN. 10.32614/CRAN.package.corrplot.

[59] Lewis, M. J., Spiliopoulou, A., Goldmann, K., Pitzalis, C., McKeigue, P. & Barnes, M. R. nestedcv: an R package for fast implementation of nested cross-validation with embedded feature selection designed for transcriptomics and high-dimensional data. *Bioinforma. Adv.* **3**, vbad048 (2023). 10.1093/bioadv/vbad048 PMC10125905 37113250

[60] Radua, J. & Koutsouleris, N. Ten Simple Rules for Using Machine Learning in Mental Health Research. *Biol. Psychiatry* **96**, 511–513 (2024). 37981177 10.1016/j.biopsych.2023.11.012

[61] Vabalas, A., Gowen, E., Poliakoff, E. & Casson, A. J. Machine learning algorithm validation with a limited sample size. *PloS One* **14**, e0224365 (2019). 31697686 10.1371/journal.pone.0224365 PMC6837442

[62] Amanollahi, M. et al. Machine learning applied to the prediction of relapse, hospitalization, and suicide in bipolar disorder using neuroimaging and clinical data: A systematic review. *J. Affect. Disord.* **361**, 778–797 (2024). 38908556 10.1016/j.jad.2024.06.061

[63] Robin, X., Turck, N., Hainard, A., Tiberti, N., Lisacek, F. & Sanchez, J.-C. et al. pROC: an open-source package for R and S+ to analyze and compare ROC curves. *BMC Bioinforma.* **12**, 77 (2011). 10.1186/1471-2105-12-77 PMC3068975 21414208

[64] Yu, Q., Plis, S., Erhardt, E., Allen, E., Sui, J., Kiehl, K. et al. Modular Organization of Functional Network Connectivity in Healthy Controls and Patients with Schizophrenia during the Resting State. *Front. Syst. Neurosci.* **5**, 103 (2012). 10.3389/fnsys.2011.00103 PMC3257855 22275887

[65] Odkhuu, S., Kim, W.S., Tsogt, U., Shen, J., Cheraghi, S., Li, L. et al. Network biomarkers in recovered psychosis patients who discontinued antipsychotics. *Mol. Psychiatry* **28**, 3717–3726 (2023). 10.1038/s41380-023-02279-6 PMC10730417 37773447

[66] Choudhary, M., Kumar, A., Tripathi, M., Bhatia, T., Shivakumar, V. & Beniwal, R. P. et al. F-18 fluorodeoxyglucose positron emission tomography study of impaired emotion processing in first episode schizophrenia. *Schizophrenia Res.* **162**, 103–107 (2015). 10.1016/j.schres.2015.01.028 PMC4339502 25655909

[67] Ding, Y., Ou, Y., Su, Q., Pan, P., Shan, X., Chen, J. et al. Enhanced Global-Brain Functional Connectivity in the Left Superior Frontal Gyrus as a Possible Endophenotype for Schizophrenia. *Front. Neurosci.* **13**, 145 (2019). 10.3389/fnins.2019.00145 PMC6399149 30863277

[68] Jeong, B., Wible, C. G., Hashimoto, R. & Kubicki, M. Functional and anatomical connectivity abnormalities in left inferior frontal gyrus in schizophrenia. *Hum. Brain Mapp.* **30**, 4138–4151 (2009). 19569073 10.1002/hbm.20835 PMC2787802

[69] Passiatore, R., Antonucci, L. A., DeRamus, T. P., Fazio, L., Stolfa, G. & Sportelli, L. et al. Changes in patterns of age-related network connectivity are associated with risk for schizophrenia. *Proc. Natl Acad. Sci. USA* **120**, e2221533120 (2023). 37527347 10.1073/pnas.2221533120 PMC10410767

[70] Zhu, Q., Huang, J. & Xu, X. Non-negative discriminative brain functional connectivity for identifying schizophrenia on resting-state fMRI. *Biomed. Eng. online* **17**, 32 (2018). 29534759 10.1186/s12938-018-0464-x PMC5851331

[71] Liu, H., Kaneko, Y., Ouyang, X., Li, L., Hao, Y. & Chen, E. Y. H. et al. Schizophrenic Patients and Their Unaffected Siblings Share Increased Resting-State Connectivity in the Task-Negative Network but Not Its Anticorrelated Task-Positive Network. *Schizophrenia Bull.* **38**, 285–294 (2010). 10.1093/schbul/sbq074 PMC3283150 20595202

[72] Li, T., Wang, Q., Zhang, J., Rolls, E. T., Yang, W. & Palaniyappan, L. et al. Brain-Wide Analysis of Functional Connectivity in First-Episode and Chronic Stages of Schizophrenia. *Schizophrenia Bull.* **43**, 436–448 (2016). 10.1093/schbul/sbw099 PMC5605268 27445261

[73] Zarei, M, J. Precentral gyrus abnormal connectivity in male and female patients with schizophrenia. *Neuroimmunol. Neuroinflamm.* **5**, 13 (2018).

[74] Lui, S., Yao, L., Xiao, Y., Keedy, S. K., Reilly, J. L. & Keefe, R. S. et al. Resting-state brain function in schizophrenia and psychotic bipolar probands and their first-degree relatives. *Psychological Med.* **45**, 97–108 (2015). 10.1017/S003329171400110X PMC5836742 25066779

[75] Cai, M., Wang, R., Liu, M., Du, X., Xue, K. & Ji, Y. et al. Disrupted local functional connectivity in schizophrenia: An updated and extended meta-analysis. *Schizophrenia* **8**, 93 (2022). 36347874 10.1038/s41537-022-00311-2 PMC9643538

[76] Li, H. J., Chan, R. C., Gong, Q. Y., Liu, Y., Liu, S. M. & Shum, D. et al. Facial emotion processing in patients with schizophrenia and their non-psychotic siblings: a functional magnetic resonance imaging study. *Schizophrenia Res.* **134**, 143–150 (2012). 10.1016/j.schres.2011.10.019 22113155

[77] Li, R.- R., Lyu, H.- L., Liu, F., Lian, N., Wu, R.- R., Zhao, J.- P. et al. Altered functional connectivity strength and its correlations with cognitive function in subjects with ultra-high risk for psychosis at rest. *CNS Neurosci. Ther.* **24,** 1140–1148 (2018). 10.1111/cns.12865 PMC6489739 29691990

[78] Koshiyama, D., Miyakoshi, M., Tanaka-Koshiyama, K., Joshi, Y. B., Molina, J. L., Sprock, J. et al. Neurophysiologic Characterization of Resting State Connectivity Abnormalities in Schizophrenia Patients. Front Psychiatry. **11**, 608154 (2020). 10.3389/fpsyt.2020.608154 PMC7729083 33329160

[79] Nyatega, C. O., Qiang, L., Adamu, M. J., Younis, A. & Kawuwa, H. B. Altered Dynamic Functional Connectivity of Cuneus in Schizophrenia Patients: A Resting-State fMRI Study. *Appl Sci.* **11**, 11392 (2021).

[80] Bergé, D., Carmona, S., Salgado, P., Rovira, M., Bulbena, A. & Vilarroya, O. Limbic activity in antipsychotic naïve first-episode psychotic subjects during facial emotion discrimination. *Eur. Arch. Psychiatry Clin. Neurosci.* **264**, 271–283 (2014). 24258969 10.1007/s00406-013-0465-5

[81] Rikandi, E., Mäntylä, T., Lindgren, M., Kieseppä, T., Suvisaari, J. & Raij, T. T. Connectivity of the precuneus-posterior cingulate cortex with the anterior cingulate cortex-medial prefrontal cortex differs consistently between control subjects and first-episode psychosis patients during a movie stimulus. *Schizophrenia Res.* **199**, 235–242 (2018). 10.1016/j.schres.2018.03.018 29588124

[82] Liang, S., Deng, W., Li, X., Wang, Q., Greenshaw, A. J. & Guo, W. et al. Aberrant posterior cingulate connectivity classify first-episode schizophrenia from controls: A machine learning study. *Schizophrenia Res.* **220**, 187–193 (2020). 10.1016/j.schres.2020.03.022 32220502

[83] Wang, H., Zeng, L.-L., Chen, Y., Yin, H., Tan, Q. & Hu, D. Evidence of a dissociation pattern in default mode subnetwork functional connectivity in schizophrenia. *Sci. Rep.* **5**, 14655 (2015). 26419213 10.1038/srep14655 PMC4588504

[84] Hua, J. P. Y., Karcher, N. R., Merrill, A. M., O’Brien, K. J., Straub, K. T. & Trull, T. J. et al. Psychosis risk is associated with decreased resting-state functional connectivity between the striatum and the default mode network. *Cogn. Affect. Behav. Neurosci.* **19**, 998–1011 (2019). 30756347 10.3758/s13415-019-00698-z PMC6690819

[85] Molent, C., Olivo, D., Wolf, R. C., Balestrieri, M. & Sambataro, F. Functional neuroimaging in treatment resistant schizophrenia: A systematic review. *Neurosci. Biobehav. Rev.* **104**, 178–190 (2019). 31276716 10.1016/j.neubiorev.2019.07.001

[86] Chen, Y., Liu, S., Zhang, B., Zhao, G., Zhang, Z. & Li, S. et al. Baseline symptom-related white matter tracts predict individualized treatment response to 12-week antipsychotic monotherapies in first-episode schizophrenia. *Transl. Psychiatry* **14**, 23 (2024). 38218952 10.1038/s41398-023-02714-w PMC10787827

[87] Ganella, E. P., Bartholomeusz, C. F., Seguin, C., Whittle, S., Bousman, C. & Phassouliotis, C. et al. Functional brain networks in treatment-resistant schizophrenia. *Schizophrenia Res.* **184**, 73–81 (2017). 10.1016/j.schres.2016.12.008 28011131

[88] Vercammen, A., Knegtering, H., den Boer, J. A., Liemburg, E. J. & Aleman, A. Auditory Hallucinations in Schizophrenia Are Associated with Reduced Functional Connectivity of the Temporo-Parietal Area. *Biol. Psychiatry* **67**, 912–918 (2010). 20060103 10.1016/j.biopsych.2009.11.017

[89] Wolf, N. D., Sambataro, F., Vasic, N., Frasch, K., Schmid, M. & Schönfeldt-Lecuona, C. et al. Dysconnectivity of multiple resting-state networks in patients with schizophrenia who have persistent auditory verbal hallucinations. *J. Psychiatry Neurosci. JPN* **36**, 366–374 (2011). 21791169 10.1503/jpn.110008 PMC3201990

[90] Alonso-Solís, A., Vives-Gilabert, Y., Grasa, E., Portella, M. J., Rabella, M. & Sauras, R. B. et al. Resting-state functional connectivity alterations in the default network of schizophrenia patients with persistent auditory verbal hallucinations. *Schizophrenia Res.* **161**, 261–268 (2015). 10.1016/j.schres.2014.10.047 25468173

[91] Thomann, P. A., Wolf, R. C., Nolte, H. M., Hirjak, D., Hofer, S. & Seidl, U. et al. Neuromodulation in response to electroconvulsive therapy in schizophrenia and major depression. *Brain Stimulation* **10**, 637–644 (2017). 28162976 10.1016/j.brs.2017.01.578

[92] Mondino, M., Jardri, R., Suaud-Chagny, M.-F., Saoud, M., Poulet, E. & Brunelin, J. Effects of Fronto-Temporal Transcranial Direct Current Stimulation on Auditory Verbal Hallucinations and Resting-State Functional Connectivity of the Left Temporo-Parietal Junction in Patients With Schizophrenia. *Schizophrenia Bull.* **42**, 318–326 (2015). 10.1093/schbul/sbv114 PMC4753593 26303936

[93] Roldán, A., Portella, M. J., Sampedro, F., Alonso-Solís, A., Sarró, S. & Rabella, M. et al. Brain metabolic changes in patients with treatment resistant schizophrenia treated with deep brain stimulation: A series of cases. *J. Psychiatr. Res.* **127**, 57–61 (2020). 32485433 10.1016/j.jpsychires.2020.05.016

[94] Egerton, A., Murphy, A., Donocik, J., Anton, A., Barker, G. J. & Collier, T. et al. Dopamine and Glutamate in Antipsychotic-Responsive Compared With Antipsychotic-Nonresponsive Psychosis: A Multicenter Positron Emission Tomography and Magnetic Resonance Spectroscopy Study (STRATA). *Schizophrenia Bull.* **47**, 505–516 (2020). 10.1093/schbul/sbaa128 PMC7965076 32910150

[95] Mouchlianitis, E., Bloomfield, M. A. P., Law, V., Beck, K., Selvaraj, S. & Rasquinha, N. et al. Treatment-Resistant Schizophrenia Patients Show Elevated Anterior Cingulate Cortex Glutamate Compared to Treatment-Responsive. *Schizophrenia Bull.* **42**, 744–752 (2015). 10.1093/schbul/sbv151 PMC4838083 26683625

[96] Matrone, M., Kotzalidis, G. D., Romano, A., Bozzao, A., Cuomo, I. & Valente, F. et al. Treatment-resistant schizophrenia: Addressing white matter integrity, intracortical glutamate levels, clinical and cognitive profiles between early- and adult-onset patients. *Prog. Neuro Psychopharmacol. Biol. psychiatry* **114**, 110493 (2022). 10.1016/j.pnpbp.2021.110493 34883221

[97] Griffiths, K., Smart, S. E., Barker, G. J., Deakin, B., Lawrie, S. M. & Lewis, S. et al. Treatment resistance NMDA receptor pathway polygenic score is associated with brain glutamate in schizophrenia. *Schizophrenia Res.* **260**, 152–159 (2023). 10.1016/j.schres.2023.08.020 PMC10873209 37657282

[98] Nakahara, T., Tsugawa, S., Noda, Y., Ueno, F., Honda, S. & Kinjo, M. et al. Glutamatergic and GABAergic metabolite levels in schizophrenia-spectrum disorders: a meta-analysis of 1H-magnetic resonance spectroscopy studies. *Mol. Psychiatry* **27**, 744–757 (2022). 34584230 10.1038/s41380-021-01297-6

[99] Iwata, Y., Nakajima, S., Plitman, E., Caravaggio, F., Kim, J. & Shah, P. et al. Glutamatergic Neurometabolite Levels in Patients With Ultra-Treatment-Resistant Schizophrenia: A Cross-Sectional 3T Proton Magnetic Resonance Spectroscopy Study. *Biol. Psychiatry* **85**, 596–605 (2019). 30389132 10.1016/j.biopsych.2018.09.009

[100] Tarumi, R., Tsugawa, S., Noda, Y., Plitman, E., Honda, S. & Matsushita, K. et al. Levels of glutamatergic neurometabolites in patients with severe treatment-resistant schizophrenia: a proton magnetic resonance spectroscopy study. *Neuropsychopharmacology* **45**, 632–640 (2020). 31842203 10.1038/s41386-019-0589-z PMC7021829

[101] Abdullah, M., Chen, K. C., Huang, L.-C., Lin, S.-H., Tseng, H.-H. & Yang, Y. K. Altered glutamate level and its association with working memory among patients with treatment-resistant schizophrenia (TRS): a proton magnetic resonance spectroscopy study. *Psychological Med.* **53**, 3220–3227 (2023). 10.1017/S003329172100533X PMC10244010 35197141

[102] Ochi, R., Plitman, E., Patel, R., Tarumi, R., Iwata, Y. & Tsugawa, S. et al. Investigating structural subdivisions of the anterior cingulate cortex in schizophrenia, with implications for treatment resistance and glutamatergic levels. *J. Psychiatry Neurosci. JPN* **47**, E1–e10 (2022). 35027443 10.1503/jpn.210113 PMC8842685

[103] Li, C. -T., Chen, M. -H., Lin, W. -C., Hong, C. -J., Yang, B. -H., Liu, R. -S. et al. The effects of low-dose ketamine on the prefrontal cortex and amygdala in treatment-resistant depression: A randomized controlled study. *Hum. Brain Mapp.* **37**, 1080–1090 (2016). 10.1002/hbm.23085 PMC6867460 26821769

[104] Li, C. -T., Yang, K. -C. & Lin, W. -C. Glutamatergic Dysfunction and Glutamatergic Compounds for Major Psychiatric Disorders: Evidence From Clinical Neuroimaging Studies. *Front. Psychiatry.* **9**, 767 (2019). 10.3389/fpsyt.2018.00767 PMC6353824 30733690

[105] Nelson, E. A., Kraguljac, N. V., Maximo, J. O., Armstrong, W. & Lahti, A. C. Dorsal striatal hypoconnectivity predicts antipsychotic medication treatment response in first-episode psychosis and unmedicated patients with schizophrenia. *Brain Behav.* **12****,** e2625 (2022). 10.1002/brb3.2625 PMC9660417 36237115

[106] Blazer, A., Chengappa, K. N. R., Foran, W., Parr, A. C., Kahn, C. E. & Luna, B. et al. Changes in corticostriatal connectivity and striatal tissue iron associated with efficacy of clozapine for treatment‑resistant schizophrenia. *Psychopharmacology* **239**, 2503–2514 (2022). 35435461 10.1007/s00213-022-06138-0 PMC9013738

[107] Gao, S., Lu, S., Shi, X., Ming, Y., Xiao, C. & Sun, J. et al. Distinguishing Between Treatment-Resistant and Non-Treatment-Resistant Schizophrenia Using Regional Homogeneity. *Front. Psychiatry* **9**, 282 (2018). 30127752 10.3389/fpsyt.2018.00282 PMC6088138

[108] Jose, M. R., Todd, L., Anita, B., Franchesica, B., Gabriela, V., Nicole, G. et al. Striatal functional connectivity in psychosis relapse: A comparison between antipsychotic adherent and non-adherent patients at the time of relapse. *medRxiv*, https://www.medrxiv.org/content/10.1101/2020.07.07.20148452v1.full (2020).

[109] Liu, L., Li, Z., Kong, D., Huang, Y., Wu, D. & Zhao, H. et al. Neuroimaging markers of aberrant brain activity and treatment response in schizophrenia patients based on brain complexity. *Transl. Psychiatry* **14**, 365 (2024). 39251595 10.1038/s41398-024-03067-8 PMC11384759

[110] Iizuka, T., Iizuka, R. & Kameyama, M. Cingulate island sign temporally changes in dementia with Lewy bodies. *Sci. Rep.* **7**, 14745 (2017). 29116145 10.1038/s41598-017-15263-2 PMC5677123

[111] De Rosa, A., Fontana, A., Nuzzo, T., Garofalo, M., Di Maio, A. & Punzo, D. et al. Machine Learning algorithm unveils glutamatergic alterations in the post-mortem schizophrenia brain. *Schizophrenia* **8**, 8 (2022). 35217646 10.1038/s41537-022-00231-1 PMC8881508

