# Supplementary material for: Addressing brain metabolic connectivity in treatment-resistant schizophrenia: a novel graph theory-driven application of 18F-FDG-PET with antipsychotic dose correction
Source: Schizophrenia (Heidelb). 2024 Dec 19;10(1):116. doi: 10.1038/s41537-024-00535-4 (PMC11659424; doi:10.1038/s41537-024-00535-4)

Supplementary Table 1. Brain regions abbreviations

Precentral_l	PrCn_l
Precentral_r	PrCn_r
Rolandic_Oper_l	RoOp_l
Rolandic_Oper_r	RoOp_r
Supp_Motor_Area_l	SMA_l
Supp_Motor_Area_r	SMA_r
Olfactory_l	Olf_l
Olfactory_r	Olf_r
Frontal_Sup_l	FS_l
Frontal_Sup_r	FS_r
Frontal_Mid_l	FM_l
Frontal_Mid_r	FM_r
Frontal_Inf_l	FI_l
Frontal_Inf_r	FI_r
Rectus_l	Rec_l
Rectus_r	Rec_r
Insula_l	Ins_l
Insula_r	Ins_r
Cingulum_Ant_l	CA_l
Cingulum_Ant_r	CA_r
Cingulum_Mid_l	CM_l

Cingulum_Mid_r	CM_r
Cingulum_Post_l	CP_l
Cingulum_Post_r	CP_r
Hippo_Parahippo_l	HiPa_l
Hippo_Parahippo_r	HiPa_r
Amygdala_l	Amy_l
Amygdala_r	Amy_r
Calcarine_l	Cal_l
Calcarine_r	Cal_r
Cuneus_l	Cun_l
Cuneus_r	Cun_r
Lingual_l	Ling_l
Lingual_r	Ling_r
Occipital_l	Occ_l
Occipital_r	Occ_r
Fusiform_l	Fus_l
Fusiform_r	Fus_r
Postcentral_l	Post_l
Postcentral_r	Post_r
SupraMarginal_l	SuMa_l
SupraMarginal_r	SuMa_r

Angular_l	Ang_l
Angular_r	Ang_r
Precuneus_l	PCun_l
Precuneus_r	PCun_r
Paracentral_Lobule_l	PaLo_l
Paracentral_Lobule_r	PaLo_r
Caudate_l	Cau_l
Caudate_r	Cau_r
Putamen_l	Put_l
Putamen_r	Put_r
Thalamus_l	Tha_l
Thalamus_r	Tha_r
Heschl_l	Hes_l
Heschl_r	Hes_r
Parietal_l	Par_l
Parietal_r	Par_r
Temporal_l	Temp_l
Temporal_r	Temp_r
Vermis	Verm
Cerebellum_Crus_l	CeCr_l
Cerebellum_Crus_r	CeCr_r
Cerebellum_l	Ce_l
Cerebellum_r	Ce_r

Supplementary Table 2. Nodes included in the default mode network (DMN) and dorsal dopaminergic pathway (DDP).

DMN	DDP
FS_l	PrCn_l
FS_r	PrCn_r
FM_l	SMA_l
FM_r	SMA_r
FI_l	FS_l
FI_r	FS_r
CA_l	FM_l
CA_r	FM_r
CP_l	FI_l
CP_r	FI_r
HiPa_l	Post_l
HiPa_r	Post_r
Cun_l	Cau_l
Cun_r	Cau_r
SuMa_l	Put_l
SuMa_r	Put_r
Ang_l	
Ang_r	
PCun_l	
PCun_r	
Temp_l	
Temp_r	

Supplementary Table 3. Demographic and clinical characteristics. Demographic characteristics, presented as medians with interquartile ranges, were assessed for normality using the Shapiro-Wilk test. Non-normally distributed variables were log-transformed for statistical analyses. Comparisons of demographic characteristics between patient and CTRL groups were performed by one-way ANOVA (age) or chi-square statistic with Yates's correction (sex). Clinical characteristics (duration of disease, education, and chlorpromazine equivalents), expressed as mean values with standard deviations (mean±SD), were compared between nTRS and TRS groups using a two-sample t-test. Variables that were significantly different between the two groups (i.e., chlorpromazine equivalents) were regarded as potential confounders in the subsequent analyses.

Demographic characteristics	Control	nTRS	TRS	Statistic	p-value (<0.05)
Subjects (total number)	16	28	26	-	-
Age (years, median [IQR])	46 [38; 54.8]	38 [28.8; 44.2]	37.5 [29.2; 46.2]	F (2,67) = 2.9529	0.059
Sex (% of Female)	43.75	46.43	11.5	X^2 = 8.5545 (df=2)	0.01
Duration of disease (years) [mean±SD]	-	13.7 ± 8.1	17.9 ± 9.5	t = -1.7294	0.09
Education (years) [mean±SD]	-	12.6 ± 3.9	11.6 ± 2.8	t = 1.0612	0.29
Chlorpromazine Equivalents [mean±SD]	-	333.6 ± 211.4	604 ± 392.7	t = -3.1163	0.003
PANSS positive [mean±SD]	-	16.6 ± 5.2	22 ± 4.4	t = -3.9967	0.002
PANSS negative [mean±SD]	-	20.2 ± 6.2	24.9 ± 4.7	t = -3.2054	0.003
PANSS general [mean±SD]	-	39.2 ± 7	51.5 ± 9.9	t = -4.9425	0.001
PANSS total [mean±SD]	-	76.1 ± 12.4	98.4 ± 16.6	t = -5.2484	0.001

Supplementary Table 4. Network robustness. Here, the sizes of the largest component after node removal, sorting the vertices by degree centrality, are reported.

CTRL	nTRS	TRS
65	65	65
64	64	64
63	63	63
62	62	62
61	61	61
60	60	60
59	59	59
58	58	58
57	57	57
56	56	56
55	55	55
54	54	54
53	53	53
52	52	52
51	51	51
50	50	50
49	49	49
48	48	48
47	47	47
46	46	46
45	45	45
44	44	44
43	43	43
42	42	42
41	41	41
40	40	40
39	39	39
38	38	38
37	37	37
36	36	36
35	35	35
34	34	34
33	33	33
32	32	32
31	31	31
30	30	30
29	29	29
28	28	28
27	27	27
26	26	26
25	25	25
24	24	24
23	23	23
22	22	22
21	21	21
20	20	20
19	19	19
18	18	18
17	17	17
16	16	16
15	14	14
14	7	13
13	7	12
12	7	11
11	6	10
10	4	9
9	4	8
8	4	7
7	4	6
5	3	6
3	3	4
2	3	4
2	2	3
1	1	2
1	1	1
0	0	0

Supplementary Table 5. Network global properties

	CTRL	nTRS	TRS
Number of nodes	65	65	65
Number of edges	1170	508	604
Edge density	0.5625	0.244231	0.290385
Global clustering coefficient	0.570303	0.222569	0.287682
Average shortest path length	1.4375	1.773077	1.714904
Small-worldness index	1.004189	0.940296	0.99127
Robustness	32.39394	31.74242	32.30303
Average degree frontal lobe	0.534036	0.313793	0.370964
Average degree insula	0.089315	0.202033	0.137099
Average degree limbic system	0.292699	0.318319	0.251314
Average degree occipital lobe	0.340159	0.369374	0.352306
Average degree parietal lobe	0.364505	0.205029	0.230142
Average degree subcortical area	0.38668	0.328207	0.324551
Average degree temporal lobe	0.134414	0.147995	0.111986
Average degree cerebellum	0.352526	0.321564	0.341262
Average degree DMN	0.748262	0.399274	0.397378
Average degree dorsal dopamine pathway	0.532086	0.315069	0.357742

Supplementary Table 6. Participation coefficient of network nodes. Modules' detection was performed by the Louvain method.

	CTRL	nTRS	TRS
PrCn_l	0.213563	0.297461	0.302328
PrCn_r	0.240631	0.289775	0.422484
RoOp_l	0.217845	0.516672	0.290902
RoOp_r	0.271418	0.270721	0.202611
SMA_l	0.239703	0.315277	0.267212
SMA_r	0.265137	0.332563	0.299701
Olf_l	0.265899	0.222448	0.184372
Olf_r	0.252816	0.308253	0.263477
FS_l	0.271723	0.500087	0.485752
FS_r	0.355761	0.244544	0.328652
FM_l	0.298364	0.299457	0.238407
FM_r	0.244309	0.362339	0.273511
FI_l	0.284854	0.228302	0.178747
FI_r	0.23958	0.217836	0.214428
Rec_l	0.303129	0.231654	0.178706
Rec_r	0.220122	0.324552	0.177026
Ins_l	0.228974	0.574169	0.29555
Ins_r	0.218795	0.439524	0.180359
CA_l	0.208668	0.241655	0.188243
CA_r	0.204386	0.503222	0.402278
CM_l	0.31318	0.255087	0.249635
CM_r	0.262287	0.264707	0.39796
CP_l	0.229296	0.363468	0.357189
CP_r	0.353289	0.269853	0.295445
HiPa_l	0.301553	0.277584	0.261183
HiPa_r	0.285311	0.256626	0.308486
Amy_l	0.299353	0.232269	0.280253
Amy_r	0.252744	0.445583	0.259385
Cal_l	0.247021	0.675333	0.298208
Cal_r	0.228379	0.510604	0.254038
Cun_l	0.286346	0.256797	0.250168
Cun_r	0.252355	0.423359	0.298666
Ling_l	0.229454	0.417397	0.290151
Ling_r	0.21743	0.382845	0.269264
Occ_l	0.231817	0.340275	0.344404
Occ_r	0.223478	0.525337	0.245648
Fus_l	0.28029	0.336691	0.28357
Fus_r	0.236925	0.27232	0.298243
Post_l	0.206078	0.339852	0.216509
Post_r	0.226974	0.525889	0.498178
SuMa_l	0.255463	0.222324	0.269454
SuMa_r	0.291226	0.469672	0.326023
Ang_l	0.266184	0.229015	0.308746
Ang_r	0.218239	0.328447	0.231349
PCun_l	0.31549	0.201912	0.650189
PCun_r	0.280728	0.39929	0.282013
PaLo_l	0.272533	0.202351	0.210579
PaLo_r	0.30962	0.302597	0.27293
Cau_l	0.272862	0.409222	0.410977
Cau_r	0.219258	0.466547	0.234585
Put_l	0.266667	0.458756	0.386539
Put_r	0.227488	0.390766	0.259744
Tha_l	0.358359	0.45864	0.296031
Tha_r	0.302289	0.363121	0.356495
Hes_l	0.22857	0.401808	0.28117
Hes_r	0.20933	0.382629	0.404101
Par_l	0.241611	0.280939	0.337015
Par_r	0.246168	0.28554	0.352237
Temp_l	0.255155	0.306761	0.238188
Temp_r	0.207747	0.296091	0.202124
Verm	0.235106	0.283943	0.168739
CeCr_l	0.273002	0.360737	0.260537
CeCr_r	0.231462	0.298181	0.193713
Ce_l	0.271929	0.348325	0.576161
Ce_r	0.289873	0.254005	0.262417

Supplementary Table 7. Weighted degree (strength) in absolute values of network nodes.

	CTRL	nTRS	TRS
PrCn_l	4.022296	1.512104	2.006805
PrCn_r	3.382257	1.576799	1.750919
RoOp_l	4.052618	1.320147	1.548226
RoOp_r	3.600765	1.905221	1.793948
SMA_l	4.084328	1.242016	2.045721
SMA_r	4.22317	1.6862	2.068568
Olf_l	3.195115	1.568313	2.129382
Olf_r	4.52518	1.688458	1.347778
FS_l	4.38306	1.43919	1.551988
FS_r	4.684132	1.778732	1.868152
FM_l	3.519154	1.604775	2.206968
FM_r	3.713065	1.540728	1.903249
FI_l	2.946953	1.70517	1.697357
FI_r	6.025182	1.738845	2.414519
Rec_l	3.41382	1.168938	1.809808
Rec_r	4.082142	1.456148	1.766069
Ins_l	4.164885	1.978277	1.291903
Ins_r	5.007892	1.59289	1.944756
CA_l	3.059545	1.70603	2.140593
CA_r	3.043835	1.036604	1.498316
CM_l	3.358137	1.51337	1.508903
CM_r	3.69913	1.502525	1.519893
CP_l	4.079582	1.720613	1.106952
CP_r	5.124757	1.744055	1.953502
HiPa_l	4.437335	1.535679	1.492973
HiPa_r	4.491014	1.71578	1.640197
Amy_l	2.71738	1.318021	1.923292
Amy_r	4.067929	1.289774	1.257722
Cal_l	3.832224	1.703286	1.893091
Cal_r	4.413559	1.179797	1.790328
Cun_l	4.386048	1.733505	1.325963
Cun_r	3.882752	2.084438	1.49614
Ling_l	3.738554	1.275674	1.831584
Ling_r	4.977345	1.759286	1.764268
Occ_l	3.233948	1.786078	1.786367
Occ_r	4.175785	1.10734	1.809884
Fus_l	5.313907	1.71026	1.323198
Fus_r	2.343304	1.241824	1.999809
Post_l	3.795813	1.686504	1.546531
Post_r	3.095154	1.366127	1.742149
SuMa_l	4.822262	1.537448	1.888375
SuMa_r	4.122841	1.168588	1.534782
Ang_l	2.631742	1.248787	2.073784
Ang_r	3.732076	1.636374	1.893041
PCun_l	4.10253	1.904804	1.40479
PCun_r	4.787884	1.587985	2.199783
PaLo_l	6.207467	2.081069	1.675694
PaLo_r	3.391751	1.416545	2.034459
Cau_l	4.165604	1.530918	1.677906
Cau_r	3.637123	1.872247	1.83715
Put_l	4.486987	1.422408	1.208668
Put_r	3.985248	1.400385	1.682366
Tha_l	4.782601	1.359056	1.578545
Tha_r	4.105692	1.365462	1.883808
Hes_l	2.766628	1.298471	1.40353
Hes_r	4.956817	1.641187	1.199267
Par_l	2.823458	1.463632	2.183361
Par_r	3.722493	1.264259	1.791571
Temp_l	4.73602	2.133373	1.803728
Temp_r	3.95976	2.015104	1.641244
Verm	3.128908	1.14957	1.967048
CeCr_l	3.001114	1.164383	1.698222
CeCr_r	4.09036	1.818673	1.631213
Ce_l	3.346259	1.665797	1.452153
Ce_r	3.406754	1.815297	1.791279

Supplementary Table 8. Permutated p-values resulting from the comparison between centrality measures (weighted degree) of nodes belonging to different networks after adjustment for multiple testing. Adjustment for chlorpromazine equivalents yielded unchanged results in the comparison between nTRS and TRS.

	nTRS vs CTRL	TRS vs CTRL	TRS vs nTRS
PrCn_l	0.0259974	0.0064994	1
PrCn_r	0.11123888	0.5849415	1
RoOp_l	0.08340833	0.3444656	1
RoOp_r	0.09884428	1	1
SMA_l	0.06196047	0.5784422	1
SMA_r	0.28278081	1	1
Olf_l	0.19469164	0.4419558	1
Olf_r	0.3262894	0.8969103	1
FS_l	0.0259974	0.0129987	1
FS_r	0.0259974	0.0064994	1
FM_l	0.0259974	0.0064994	1
FM_r	0.0496314	0.0259974	1
FI_l	0.0496314	0.0389961	1
FI_r	0.06196047	0.1234877	1
Rec_l	0.19181866	0.2859714	1
Rec_r	0.06196047	0.0324968	1
Ins_l	0.09630855	0.1689831	1
Ins_r	0.06996359	0.1494851	1
CA_l	0.09164084	0.3184682	1
CA_r	0.19469164	1	1
CM_l	0.26559844	0.7474253	1
CM_r	0.0496314	0.0194981	1
CP_l	0.19469164	0.8059194	1
CP_r	0.0496314	0.1039896	1
HiPa_l	0.28515898	1	1
HiPa_r	0.16187444	0.909909	1
Amy_l	0.10346965	1	1
Amy_r	0.19469164	1	1
Cal_l	0.06996359	0.0129987	1
Cal_r	0.0259974	0.0064994	1
Cun_l	0.19361222	1	1
Cun_r	0.51903403	1	1
Ling_l	0.06196047	0.0649935	1
Ling_r	0.20529469	0.0129987	1
Occ_l	0.18810976	1	1
Occ_r	0.2339766	0.5784422	1
Fus_l	0.19469164	0.6434357	1
Fus_r	0.17146815	0.0064994	1
Post_l	0.20703138	0.3964604	1
Post_r	0.43189229	1	1
SuMa_l	0.21686607	0.8059194	1
SuMa_r	0.51076638	1	1
Ang_l	0.28278081	0.5589441	1
Ang_r	0.19469164	0.0389961	1
PCun_l	0.3262894	1	1

PCun_r	0.1685892	0.6694331	1
PaLo_l	0.32245898	1	1
PaLo_r	0.09284786	0.1819818	1
Cau_l	0.16187444	0.5264474	1
Cau_r	0.34744886	1	1
Put_l	0.0496314	0.0259974	1
Put_r	0.02924708	0.0064994	1
Tha_l	0.14762809	0.3314669	1
Tha_r	0.09720767	0.3639636	1
Hes_l	0.14762809	0.6889311	1

Hes_r	0.27959468	1	1
Par_l	0.33038363	0.8319168	1
Par_r	0.57054295	1	1
Temp_l	0.0885964	0.3769623	1
Temp_r	0.23046695	1	1
Verm	0.20703138	0.8514149	1
CeCr_l	0.19181866	0.6629337	1
CeCr_r	0.15121821	1	1
Ce_l	0.15121821	0.1169883	1
Ce_r	0.19469164	0.1884812	1

Supplementary Table 9. Permutated p-values resulting from the comparison between paired edges of nodes comprised in the DMN of nTRS vs CTRL groups.

	FS_l	FS_r	FM_l	FM_r	FI_l	FI_r	CA_l	CA_r	CP_l	CP_r	HiPa_l	HiPa_r	Cun_l	Cun_r	SuMa_l	SuMa_r	Ang_l	Ang_r	PCun_l	PCun_r	Temp_l	Temp_r
FS_l	1.00	0.33	0.20	0.81	0.78	1.00	0.05	0.67	1.00	0.10	1.00	0.34	0.22	0.12	0.65	0.24	0.27	0.31	0.23	1.00	0.10	0.53
FS_r	0.33	1.00	0.05	0.54	0.26	0.53	0.07	0.45	0.74	0.13	0.57	0.56	0.01	0.48	0.92	0.36	0.12	0.22	0.23	0.20	1.00	0.47
FM_l	0.20	0.05	1.00	0.70	0.31	1.00	0.77	0.38	0.40	1.00	0.11	0.27	0.26	1.00	0.15	0.14	0.31	1.00	0.41	0.52	0.29	0.75
FM_r	0.81	0.54	0.70	1.00	0.33	0.79	0.39	0.60	0.06	0.43	0.31	0.06	0.75	0.65	0.28	0.07	0.32	0.50	0.84	0.20	0.56	0.47
FI_l	0.78	0.26	0.31	0.33	1.00	0.15	0.38	0.45	0.54	0.14	0.05	0.24	0.13	0.24	0.64	0.45	0.49	0.46	0.68	1.00	0.06	0.30
FI_r	1.00	0.53	1.00	0.79	0.15	1.00	0.10	0.09	1.00	0.80	0.85	0.06	1.00	0.21	0.52	0.58	0.55	0.13	0.40	0.18	0.07	0.95
CA_l	0.05	0.07	0.77	0.39	0.38	0.10	1.00	0.98	0.06	1.00	0.31	0.36	0.20	0.25	0.30	0.37	0.27	1.00	0.04	0.69	0.50	0.21
CA_r	0.67	0.45	0.38	0.60	0.45	0.09	0.98	1.00	0.51	0.54	1.00	1.00	0.47	0.69	0.06	0.10	0.55	0.37	0.09	0.53	0.52	0.03
CP_l	1.00	0.74	0.40	0.06	0.54	1.00	0.06	0.51	1.00	0.34	0.39	0.66	0.50	0.33	0.16	0.19	0.30	0.15	0.10	0.43	0.33	0.14
CP_r	0.10	0.13	1.00	0.43	0.14	0.80	1.00	0.54	0.34	1.00	0.12	0.08	0.01	0.02	1.00	0.23	0.36	0.49	0.27	0.41	0.43	0.69
HiPa_l	1.00	0.57	0.11	0.31	0.05	0.85	0.31	1.00	0.39	0.12	1.00	0.94	0.58	0.29	1.00	1.00	1.00	0.71	0.26	0.80	0.07	0.43
HiPa_r	0.34	0.56	0.27	0.06	0.24	0.06	0.36	1.00	0.66	0.08	0.94	1.00	0.66	1.00	0.08	0.33	0.53	0.33	1.00	0.14	0.24	0.42
Cun_l	0.22	0.01	0.26	0.75	0.13	1.00	0.20	0.47	0.50	0.01	0.58	0.66	1.00	0.43	0.67	0.57	0.11	0.09	0.08	0.35	0.67	0.70
Cun_r	0.12	0.48	1.00	0.65	0.24	0.21	0.25	0.69	0.33	0.02	0.29	1.00	0.43	1.00	0.56	0.61	0.47	1.00	0.55	0.17	0.70	0.69
SuMa_l	0.65	0.92	0.15	0.28	0.64	0.52	0.30	0.06	0.16	1.00	1.00	0.08	0.67	0.56	1.00	0.15	0.71	0.64	0.59	0.65	0.92	0.08

SuMa_r	0.24	0.36	0.14	0.07	0.45	0.58	0.37	0.10	0.19	0.23	1.00	0.33	0.57	0.61	0.15	1.00	0.76	1.00	0.75	0.50	0.26	0.03
Ang_l	0.27	0.12	0.31	0.32	0.49	0.55	0.27	0.55	0.30	0.36	1.00	0.53	0.11	0.47	0.71	0.76	1.00	0.06	1.00	0.83	0.41	0.25
Ang_r	0.31	0.22	1.00	0.50	0.46	0.13	1.00	0.37	0.15	0.49	0.71	0.33	0.09	1.00	0.64	1.00	0.06	1.00	1.00	0.39	0.30	0.64
PCun_l	0.23	0.23	0.41	0.84	0.68	0.40	0.04	0.09	0.10	0.27	0.26	1.00	0.08	0.55	0.59	0.75	1.00	1.00	1.00	0.86	0.56	0.78
PCun_r	1.00	0.20	0.52	0.20	1.00	0.18	0.69	0.53	0.43	0.41	0.80	0.14	0.35	0.17	0.65	0.50	0.83	0.39	0.86	1.00	0.31	0.44
Temp_l	0.10	1.00	0.29	0.56	0.06	0.07	0.50	0.52	0.33	0.43	0.07	0.24	0.67	0.70	0.92	0.26	0.41	0.30	0.56	0.31	1.00	0.74
Temp_r	0.53	0.47	0.75	0.47	0.30	0.95	0.21	0.03	0.14	0.69	0.43	0.42	0.70	0.69	0.08	0.03	0.25	0.64	0.78	0.44	0.74	1.00

Supplementary Table 10. Permutated p-values resulting from the comparison between paired edges of nodes comprised in the DMN of TRS vs CTRL groups.

	FS_l	FS_r	FM_l	FM_r	FI_l	FI_r	CA_l	CA_r	CP_l	CP_r	HiPa_l	HiPa_r	Cun_l	Cun_r	SuMa_l	SuMa_r	Ang_l	Ang_r	PCun_l	PCun_r	Temp_l	Temp_r
FS_l	1.00	0.31	0.17	0.88	1.00	1.00	0.04	0.18	0.43	0.21	0.53	0.26	0.35	0.25	1.00	0.15	0.06	0.25	0.19	0.62	0.01	0.63
FS_r	0.31	1.00	0.04	0.67	0.11	0.95	0.03	0.35	0.60	0.11	0.54	0.44	0.01	0.16	0.44	0.29	0.04	0.14	0.05	0.28	1.00	0.69
FM_l	0.17	0.04	1.00	0.77	0.08	1.00	0.44	0.24	0.43	0.55	0.38	0.85	0.15	0.39	0.12	0.16	0.27	1.00	0.30	0.29	0.41	1.00
FM_r	0.88	0.67	0.77	1.00	0.19	0.57	0.43	0.56	0.00	0.27	0.67	0.07	0.56	0.89	0.17	0.10	0.30	0.73	0.63	0.21	0.52	0.89
FI_l	1.00	0.11	0.08	0.19	1.00	0.04	0.05	0.13	0.43	0.14	0.02	0.85	0.09	0.33	0.56	0.61	0.36	0.32	0.17	1.00	0.15	0.30
FI_r	1.00	0.95	1.00	0.57	0.04	1.00	0.01	0.12	1.00	0.54	0.59	0.03	0.78	0.25	0.86	0.82	1.00	0.10	0.40	0.25	0.17	0.42
CA_l	0.04	0.03	0.44	0.43	0.05	0.01	1.00	0.45	0.05	1.00	0.40	0.40	0.21	0.45	0.22	0.66	0.42	0.46	0.35	0.78	0.57	0.05
CA_r	0.18	0.35	0.24	0.56	0.13	0.12	0.45	1.00	0.30	0.62	0.77	1.00	0.61	0.71	0.08	0.08	0.30	0.32	0.16	0.68	1.00	0.06
CP_l	0.43	0.60	0.43	0.00	0.43	1.00	0.05	0.30	1.00	0.10	0.21	0.80	0.78	0.81	0.17	0.15	0.19	0.18	0.13	0.62	0.27	0.23
CP_r	0.21	0.11	0.55	0.27	0.14	0.54	1.00	0.62	0.10	1.00	0.11	0.05	0.03	0.02	1.00	0.11	1.00	0.61	0.17	0.33	0.67	0.47
HiPa_l	0.53	0.54	0.38	0.67	0.02	0.59	0.40	0.77	0.21	0.11	1.00	1.00	0.48	0.45	1.00	0.62	1.00	0.71	0.32	0.63	0.04	0.48
HiPa_r	0.26	0.44	0.85	0.07	0.85	0.03	0.40	1.00	0.80	0.05	1.00	1.00	0.30	1.00	0.35	0.28	0.61	0.66	1.00	0.25	0.26	0.63
Cun_l	0.35	0.01	0.15	0.56	0.09	0.78	0.21	0.61	0.78	0.03	0.48	0.30	1.00	0.52	0.85	0.76	0.15	0.01	0.10	0.35	0.51	0.70
Cun_r	0.25	0.16	0.39	0.89	0.33	0.25	0.45	0.71	0.81	0.02	0.45	1.00	0.52	1.00	0.80	0.76	0.30	1.00	0.50	0.30	1.00	0.73
SuMa_l	1.00	0.44	0.12	0.17	0.56	0.86	0.22	0.08	0.17	1.00	1.00	0.35	0.85	0.80	1.00	0.64	0.98	0.43	0.65	0.68	0.82	0.37

SuMa_r	0.15	0.29	0.16	0.10	0.61	0.82	0.66	0.08	0.15	0.11	0.62	0.28	0.76	0.76	0.64	1.00	0.59	0.52	0.86	0.51	0.23	0.02
Ang_l	0.06	0.04	0.27	0.30	0.36	1.00	0.42	0.30	0.19	1.00	1.00	0.61	0.15	0.30	0.98	0.59	1.00	0.28	1.00	0.76	0.38	0.30
Ang_r	0.25	0.14	1.00	0.73	0.32	0.10	0.46	0.32	0.18	0.61	0.71	0.66	0.01	1.00	0.43	0.52	0.28	1.00	1.00	0.40	0.28	0.71
PCun_l	0.19	0.05	0.30	0.63	0.17	0.40	0.35	0.16	0.13	0.17	0.32	1.00	0.10	0.50	0.65	0.86	1.00	1.00	1.00	0.43	0.78	1.00
PCun_r	0.62	0.28	0.29	0.21	1.00	0.25	0.78	0.68	0.62	0.33	0.63	0.25	0.35	0.30	0.68	0.51	0.76	0.40	0.43	1.00	0.29	0.53
Temp_l	0.01	1.00	0.41	0.52	0.15	0.17	0.57	1.00	0.27	0.67	0.04	0.26	0.51	1.00	0.82	0.23	0.38	0.28	0.78	0.29	1.00	0.50
Temp_r	0.63	0.69	1.00	0.89	0.30	0.42	0.05	0.06	0.23	0.47	0.48	0.63	0.70	0.73	0.37	0.02	0.30	0.71	1.00	0.53	0.50	1.00

Supplementary Table 11. Permutated p-values resulting from the comparison between paired edges of nodes comprised in the DMN of TRS vs nTRS groups without adjustment for chlorpromazine equivalents.

	FS_l	FS_r	FM_l	FM_r	FI_l	FI_r	CA_l	CA_r	CP_l	CP_r	HiPa_l	HiPa_r	Cun_l	Cun_r	SuMa_l	SuMa_r	Ang_l	Ang_r	PCun_l	PCun_r	Temp_l	Temp_r
FS_l	1.00	0.65	0.89	0.79	0.81	1.00	1.00	0.33	0.03	0.01	0.08	1.00	0.31	0.01	0.20	1.00	0.29	1.00	0.05	0.15	1.00	1.00
FS_r	0.65	1.00	0.65	0.81	1.00	0.57	0.18	0.59	1.00	1.00	0.57	1.00	0.72	0.13	0.11	1.00	1.00	1.00	0.12	0.16	1.00	0.02
FM_l	0.89	0.65	1.00	0.43	0.51	1.00	1.00	0.30	0.27	0.11	0.28	0.07	0.88	0.06	1.00	1.00	1.00	1.00	1.00	0.22	1.00	0.57
FM_r	0.79	0.81	0.43	1.00	1.00	0.27	1.00	1.00	1.00	1.00	0.30	1.00	0.34	0.85	1.00	0.37	1.00	0.41	0.18	1.00	0.94	0.16
FI_l	0.81	1.00	0.51	1.00	1.00	0.79	0.02	0.25	1.00	1.00	1.00	0.24	1.00	0.31	1.00	0.76	0.99	1.00	0.15	1.00	0.78	0.94
FI_r	1.00	0.57	1.00	0.27	0.79	1.00	1.00	1.00	1.00	1.00	0.41	0.77	0.33	1.00	0.19	0.43	0.50	1.00	1.00	1.00	1.00	0.45
CA_l	1.00	0.18	1.00	1.00	0.02	1.00	1.00	0.21	1.00	1.00	0.89	1.00	0.44	0.43	1.00	0.14	1.00	0.18	0.36	1.00	1.00	0.06
CA_r	0.33	0.59	0.30	1.00	0.25	1.00	0.21	1.00	0.08	1.00	0.41	1.00	1.00	0.63	1.00	1.00	0.08	0.39	0.26	1.00	0.25	1.00
CP_l	0.03	1.00	0.27	1.00	1.00	1.00	1.00	0.08	1.00	0.35	0.21	1.00	0.30	0.06	0.52	1.00	1.00	1.00	1.00	1.00	1.00	1.00
CP_r	0.01	1.00	0.11	1.00	1.00	1.00	1.00	1.00	0.35	1.00	1.00	0.40	1.00	1.00	1.00	0.14	0.08	1.00	0.99	0.59	0.35	0.03
HiPa_l	0.08	0.57	0.28	0.30	1.00	0.41	0.89	0.41	0.21	1.00	1.00	0.93	0.37	1.00	1.00	0.77	1.00	1.00	1.00	0.18	0.91	1.00
HiPa_r	1.00	1.00	0.07	1.00	0.24	0.77	1.00	1.00	1.00	0.40	0.93	1.00	0.39	1.00	0.29	1.00	0.28	0.05	1.00	1.00	1.00	0.69
Cun_l	0.31	0.72	0.88	0.34	1.00	0.33	0.44	1.00	0.30	1.00	0.37	0.39	1.00	0.99	1.00	0.75	1.00	0.05	1.00	0.15	0.51	0.46
Cun_r	0.01	0.13	0.06	0.85	0.31	1.00	0.43	0.63	0.06	1.00	1.00	1.00	0.99	1.00	0.28	1.00	0.17	1.00	1.00	0.13	0.74	1.00
SuMa_l	0.20	0.11	1.00	1.00	1.00	0.19	1.00	1.00	0.52	1.00	1.00	0.29	1.00	0.28	1.00	0.12	0.61	1.00	0.33	0.36	0.71	0.53
SuMa_r	1.00	1.00	1.00	0.37	0.76	0.43	0.14	1.00	1.00	0.14	0.77	1.00	0.75	1.00	0.12	1.00	1.00	0.43	1.00	0.70	0.21	0.72

Ang_l	0.29	1.00	1.00	1.00	0.99	0.50	1.00	0.08	1.00	0.08	1.00	0.28	1.00	0.17	0.61	1.00	1.00	0.22	1.00	1.00	0.97	1.00
Ang_r	1.00	1.00	1.00	0.41	1.00	1.00	0.18	0.39	1.00	1.00	1.00	0.05	0.05	1.00	1.00	0.43	0.22	1.00	1.00	0.98	0.89	1.00
PCun_l	0.05	0.12	1.00	0.18	0.15	1.00	0.36	0.26	1.00	0.99	1.00	1.00	1.00	1.00	0.33	1.00	1.00	1.00	1.00	0.16	1.00	0.39
PCun_r	0.15	0.16	0.22	1.00	1.00	1.00	1.00	1.00	1.00	0.59	0.18	1.00	0.15	0.13	0.36	0.70	1.00	0.98	0.16	1.00	0.37	1.00
Temp_l	1.00	1.00	1.00	0.94	0.78	1.00	1.00	0.25	1.00	0.35	0.91	1.00	0.51	0.74	0.71	0.21	0.97	0.89	1.00	0.37	1.00	0.67
Temp_r	1.00	0.02	0.57	0.16	0.94	0.45	0.06	1.00	1.00	0.03	1.00	0.69	0.46	1.00	0.53	0.72	1.00	1.00	0.39	1.00	0.67	1.00

Supplementary Table 12. Permutated p-values resulting from the comparison between paired edges of nodes comprised in the DMN of TRS vs nTRS groups after adjustment for chlorpromazine equivalents.

	FS_l	FS_r	FM_l	FM_r	FI_l	FI_r	CA_l	CA_r	CP_l	CP_r	HiPa_l	HiPa_r	Cun_l	Cun_r	SuMa_l	SuMa_r	Ang_l	Ang_r	PCun_l	PCun_r	Temp_l	Temp_r
FS_l	1.00	0.80	0.72	0.68	0.76	1.00	1.00	0.34	0.01	0.00	0.05	1.00	0.30	0.00	0.19	1.00	0.29	1.00	0.03	0.09	1.00	1.00
FS_r	0.80	1.00	0.79	0.88	1.00	0.54	1.00	0.54	1.00	1.00	0.53	1.00	0.72	0.12	0.12	1.00	1.00	1.00	0.05	0.14	1.00	0.01
FM_l	0.72	0.79	1.00	0.54	0.46	1.00	1.00	0.34	0.23	0.07	0.23	0.09	1.00	0.07	1.00	1.00	1.00	1.00	1.00	0.17	1.00	1.00
FM_r	0.68	0.88	0.54	1.00	1.00	0.23	1.00	1.00	1.00	1.00	0.31	1.00	0.32	0.81	1.00	0.36	1.00	0.55	0.15	1.00	0.60	0.08
FI_l	0.76	1.00	0.46	1.00	1.00	0.78	0.01	0.24	1.00	1.00	1.00	0.38	1.00	0.34	1.00	0.61	1.00	1.00	0.15	1.00	0.56	0.84
FI_r	1.00	0.54	1.00	0.23	0.78	1.00	1.00	1.00	1.00	1.00	0.59	0.73	0.34	1.00	0.07	0.51	0.54	1.00	1.00	1.00	1.00	0.62
CA_l	1.00	1.00	1.00	1.00	0.01	1.00	1.00	0.32	1.00	1.00	0.97	1.00	0.35	0.45	1.00	0.09	1.00	0.15	0.39	1.00	1.00	0.04
CA_r	0.34	0.54	0.34	1.00	0.24	1.00	0.32	1.00	0.06	1.00	0.30	1.00	1.00	0.64	1.00	1.00	0.05	1.00	0.24	1.00	0.29	1.00
CP_l	0.01	1.00	0.23	1.00	1.00	1.00	1.00	0.06	1.00	0.30	1.00	1.00	0.19	0.07	0.69	1.00	1.00	1.00	1.00	1.00	1.00	1.00
CP_r	0.00	1.00	0.07	1.00	1.00	1.00	1.00	1.00	0.30	1.00	1.00	0.31	1.00	1.00	1.00	0.11	0.14	1.00	0.94	0.66	0.28	0.02
HiPa_l	0.05	0.53	0.23	0.31	1.00	0.59	0.97	0.30	1.00	1.00	1.00	0.73	0.33	1.00	1.00	0.71	1.00	1.00	1.00	0.20	0.77	1.00
HiPa_r	1.00	1.00	0.09	1.00	0.38	0.73	1.00	1.00	1.00	0.31	0.73	1.00	0.46	1.00	0.22	1.00	0.35	0.05	1.00	1.00	1.00	0.88
Cun_l	0.30	0.72	1.00	0.32	1.00	0.34	0.35	1.00	0.19	1.00	0.33	0.46	1.00	0.98	1.00	0.76	1.00	0.03	1.00	0.10	0.48	0.51
Cun_r	0.00	0.12	0.07	0.81	0.34	1.00	0.45	0.64	0.07	1.00	1.00	1.00	0.98	1.00	0.38	1.00	0.11	1.00	1.00	0.13	0.67	1.00
SuMa_l	0.19	0.12	1.00	1.00	1.00	0.07	1.00	1.00	0.69	1.00	1.00	0.22	1.00	0.38	1.00	0.09	0.63	0.83	0.21	0.40	0.67	0.41
SuMa_r	1.00	1.00	1.00	0.36	0.61	0.51	0.09	1.00	1.00	0.11	0.71	1.00	0.76	1.00	0.09	1.00	1.00	0.39	1.00	1.00	0.13	0.77

Ang_l	0.29	1.00	1.00	1.00	1.00	0.54	1.00	0.05	1.00	0.14	1.00	0.35	1.00	0.11	0.63	1.00	1.00	0.15	1.00	1.00	0.86	1.00
Ang_r	1.00	1.00	1.00	0.55	1.00	1.00	0.15	1.00	1.00	1.00	1.00	0.05	0.03	1.00	0.83	0.39	0.15	1.00	1.00	0.85	1.00	1.00
PCun_l	0.03	0.05	1.00	0.15	0.15	1.00	0.39	0.24	1.00	0.94	1.00	1.00	1.00	1.00	0.21	1.00	1.00	1.00	1.00	0.11	1.00	0.47
PCun_r	0.09	0.14	0.17	1.00	1.00	1.00	1.00	1.00	1.00	0.66	0.20	1.00	0.10	0.13	0.40	1.00	1.00	0.85	0.11	1.00	0.35	1.00
Temp_l	1.00	1.00	1.00	0.60	0.56	1.00	1.00	0.29	1.00	0.28	0.77	1.00	0.48	0.67	0.67	0.13	0.86	1.00	1.00	0.35	1.00	0.77
Temp_r	1.00	0.01	1.00	0.08	0.84	0.62	0.04	1.00	1.00	0.02	1.00	0.88	0.51	1.00	0.41	0.77	1.00	1.00	0.47	1.00	0.77	1.00

Supplementary Table 13. Permutated p-values resulting from the comparison between paired edges of nodes comprised in the dorsal dopamine pathway of nTRS vs CTRL groups.

	PrCn_l	PrCn_r	SMA_l	SMA_r	FS_l	FS_r	FM_l	FM_r	FI_l	FI_r	Post_l	Post_r	Cau_l	Cau_r	Put_l	Put_r
PrCn_l	1.00	0.46	0.38	1.00	0.43	0.41	0.37	1.00	0.83	0.61	0.42	0.93	0.70	1.00	0.94	0.75
PrCn_r	0.46	1.00	0.48	0.63	1.00	0.85	0.69	0.55	0.22	1.00	1.00	0.86	0.42	0.39	1.00	0.72
SMA_l	0.38	0.48	1.00	0.60	0.51	0.28	1.00	0.52	0.79	0.93	0.34	1.00	1.00	0.77	0.17	0.38
SMA_r	1.00	0.63	0.60	1.00	0.07	0.71	1.00	0.40	0.65	1.00	0.55	0.70	0.26	0.72	1.00	1.00
FS_l	0.43	1.00	0.51	0.07	1.00	0.45	0.80	0.82	0.51	1.00	0.25	1.00	1.00	1.00	0.20	1.00
FS_r	0.41	0.85	0.28	0.71	0.45	1.00	1.00	0.80	1.00	0.98	0.32	0.75	0.52	0.86	0.79	0.13
FM_l	0.37	0.69	1.00	1.00	0.80	1.00	1.00	0.05	0.66	0.21	0.67	0.23	0.44	1.00	0.26	1.00
FM_r	1.00	0.55	0.52	0.40	0.82	0.80	0.05	1.00	1.00	0.14	0.78	0.20	0.20	1.00	1.00	0.57
FI_l	0.83	0.22	0.79	0.65	0.51	1.00	0.66	1.00	1.00	0.98	0.39	0.75	0.83	1.00	1.00	0.63
FI_r	0.61	1.00	0.93	1.00	1.00	0.98	0.21	0.14	0.98	1.00	0.39	0.41	0.51	0.55	1.00	0.69
Post_l	0.42	1.00	0.34	0.55	0.25	0.32	0.67	0.78	0.39	0.39	1.00	0.53	1.00	0.29	0.35	1.00
Post_r	0.93	0.86	1.00	0.70	1.00	0.75	0.23	0.20	0.75	0.41	0.53	1.00	0.46	0.52	0.32	1.00
Cau_l	0.70	0.42	1.00	0.26	1.00	0.52	0.44	0.20	0.83	0.51	1.00	0.46	1.00	0.75	0.29	0.42
Cau_r	1.00	0.39	0.77	0.72	1.00	0.86	1.00	1.00	1.00	0.55	0.29	0.52	0.75	1.00	1.00	0.65
Put_l	0.94	1.00	0.17	1.00	0.20	0.79	0.26	1.00	1.00	1.00	0.35	0.32	0.29	1.00	1.00	0.22
Put_r	0.75	0.72	0.38	1.00	1.00	0.13	1.00	0.57	0.63	0.69	1.00	1.00	0.42	0.65	0.22	1.00

Supplementary Table 14. Permutated p-values resulting from the comparison between paired edges of nodes comprised in the dorsal dopamine pathway of TRS vs CTRL groups.

	PrCn_l	PrCn_r	SMA_l	SMA_r	FS_l	FS_r	FM_l	FM_r	FI_l	FI_r	Post_l	Post_r	Cau_l	Cau_r	Put_l	Put_r
PrCn_l	1.00	0.21	0.09	1.00	0.28	1.00	0.53	1.00	0.98	0.22	0.46	0.42	0.51	1.00	0.58	0.46
PrCn_r	0.21	1.00	1.00	0.59	0.82	0.56	1.00	0.09	0.89	0.53	1.00	0.86	1.00	0.54	0.58	0.66
SMA_l	0.09	1.00	1.00	0.19	0.84	0.44	0.50	0.75	0.71	0.88	0.59	1.00	0.43	0.34	0.36	1.00
SMA_r	1.00	0.59	0.19	1.00	0.08	0.55	0.09	1.00	1.00	1.00	0.94	1.00	0.48	1.00	0.82	1.00
FS_l	0.28	0.82	0.84	0.08	1.00	0.74	0.82	1.00	0.57	1.00	0.33	0.56	1.00	1.00	0.80	0.50
FS_r	1.00	0.56	0.44	0.55	0.74	1.00	0.51	0.84	1.00	0.96	0.05	0.73	1.00	0.57	0.47	0.03
FM_l	0.53	1.00	0.50	0.09	0.82	0.51	1.00	0.10	0.99	0.18	1.00	0.13	0.39	0.29	0.06	0.78
FM_r	1.00	0.09	0.75	1.00	1.00	0.84	0.10	1.00	1.00	0.79	0.77	0.94	0.74	1.00	1.00	1.00
FI_l	0.98	0.89	0.71	1.00	0.57	1.00	0.99	1.00	1.00	0.86	0.02	0.21	0.71	0.71	1.00	0.89
FI_r	0.22	0.53	0.88	1.00	1.00	0.96	0.18	0.79	0.86	1.00	0.78	1.00	0.66	0.47	0.54	0.39
Post_l	0.46	1.00	0.59	0.94	0.33	0.05	1.00	0.77	0.02	0.78	1.00	0.82	1.00	0.31	0.22	1.00
Post_r	0.42	0.86	1.00	1.00	0.56	0.73	0.13	0.94	0.21	1.00	0.82	1.00	0.76	0.69	0.04	1.00
Cau_l	0.51	1.00	0.43	0.48	1.00	1.00	0.39	0.74	0.71	0.66	1.00	0.76	1.00	0.51	0.83	0.51
Cau_r	1.00	0.54	0.34	1.00	1.00	0.57	0.29	1.00	0.71	0.47	0.31	0.69	0.51	1.00	0.84	1.00
Put_l	0.58	0.58	0.36	0.82	0.80	0.47	0.06	1.00	1.00	0.54	0.22	0.04	0.83	0.84	1.00	0.12
Put_r	0.46	0.66	1.00	1.00	0.50	0.03	0.78	1.00	0.89	0.39	1.00	1.00	0.51	1.00	0.12	1.00

Supplementary Table 15. Permutated p-values resulting from the comparison between paired edges of nodes comprised in the dorsal dopamine pathway of TRS vs nTRS groups without adjustment for chlorpromazine equivalents.

	PrCn_l	PrCn_r	SMA_l	SMA_r	FS_l	FS_r	FM_l	FM_r	FI_l	FI_r	Post_l	Post_r	Cau_l	Cau_r	Put_l	Put_r
PrCn_l	1.00	0.38	0.18	1.00	1.00	0.16	0.11	1.00	0.81	0.49	0.87	0.21	0.06	1.00	0.30	0.48
PrCn_r	0.38	1.00	0.52	0.93	0.61	0.21	0.43	0.23	0.29	0.20	1.00	0.63	0.28	1.00	0.26	0.17
SMA_l	0.18	0.52	1.00	0.01	0.45	0.89	0.23	0.46	0.90	0.84	0.85	1.00	0.22	0.41	1.00	0.37
SMA_r	1.00	0.93	0.01	1.00	0.39	0.16	0.01	0.09	0.48	1.00	0.46	0.62	0.50	0.49	0.81	1.00
FS_l	1.00	0.61	0.45	0.39	1.00	0.70	0.94	0.80	1.00	1.00	0.17	0.45	1.00	1.00	0.30	0.24
FS_r	0.16	0.21	0.89	0.16	0.70	1.00	0.71	0.61	1.00	0.94	0.19	0.40	0.60	0.51	0.21	0.58
FM_l	0.11	0.43	0.23	0.01	0.94	0.71	1.00	0.49	0.60	1.00	0.66	1.00	1.00	0.15	1.00	0.24
FM_r	1.00	0.23	0.46	0.09	0.80	0.61	0.49	1.00	1.00	0.32	0.42	0.14	0.25	1.00	1.00	0.49
FI_l	0.81	0.29	0.90	0.48	1.00	1.00	0.60	1.00	1.00	0.84	0.19	0.28	0.76	0.59	1.00	0.54
FI_r	0.49	0.20	0.84	1.00	1.00	0.94	1.00	0.32	0.84	1.00	0.46	0.36	0.25	1.00	0.35	1.00
Post_l	0.87	1.00	0.85	0.46	0.17	0.19	0.66	0.42	0.19	0.46	1.00	0.61	1.00	0.01	0.37	1.00
Post_r	0.21	0.63	1.00	0.62	0.45	0.40	1.00	0.14	0.28	0.36	0.61	1.00	0.86	0.85	0.11	1.00
Cau_l	0.06	0.28	0.22	0.50	1.00	0.60	1.00	0.25	0.76	0.25	1.00	0.86	1.00	0.79	0.57	0.83
Cau_r	1.00	1.00	0.41	0.49	1.00	0.51	0.15	1.00	0.59	1.00	0.01	0.85	0.79	1.00	0.65	0.84
Put_l	0.30	0.26	1.00	0.81	0.30	0.21	1.00	1.00	1.00	0.35	0.37	0.11	0.57	0.65	1.00	0.57
Put_r	0.48	0.17	0.37	1.00	0.24	0.58	0.24	0.49	0.54	1.00	1.00	1.00	0.83	0.84	0.57	1.00

Supplementary Table 16. Permutated p-values resulting from the comparison between paired edges of nodes comprised in the dorsal dopamine pathway of TRS vs nTRS groups after adjustment for chlorpromazine equivalents.

	PrCn_l	PrCn_r	SMA_l	SMA_r	FS_l	FS_r	FM_l	FM_r	FI_l	FI_r	Post_l	Post_r	Cau_l	Cau_r	Put_l	Put_r
PrCn_l	1.00	0.40	0.15	1.00	1.00	0.15	0.06	1.00	0.77	0.41	0.90	0.15	0.07	1.00	0.23	0.63
PrCn_r	0.40	1.00	0.45	0.97	0.68	0.14	0.27	0.12	0.30	0.23	1.00	0.66	0.32	1.00	0.17	0.11
SMA_l	0.15	0.45	1.00	0.01	0.36	0.81	0.18	0.35	0.88	0.81	0.72	1.00	0.16	0.26	1.00	0.35
SMA_r	1.00	0.97	0.01	1.00	1.00	0.13	0.01	0.06	0.44	1.00	0.51	0.52	0.38	0.37	0.81	1.00
FS_l	1.00	0.68	0.36	1.00	1.00	0.67	0.97	0.82	1.00	1.00	0.12	1.00	1.00	0.27	0.27	0.23
FS_r	0.15	0.14	0.81	0.13	0.67	1.00	0.68	0.58	1.00	0.95	0.22	0.26	0.62	0.33	0.13	0.67
FM_l	0.06	0.27	0.18	0.01	0.97	0.68	1.00	0.42	0.59	1.00	0.46	1.00	1.00	0.10	1.00	0.23
FM_r	1.00	0.12	0.35	0.06	0.82	0.58	0.42	1.00	1.00	0.31	0.43	0.14	0.23	1.00	1.00	0.43
FI_l	0.77	0.30	0.88	0.44	1.00	1.00	0.59	1.00	1.00	0.75	0.20	0.34	0.80	0.53	1.00	0.49
FI_r	0.41	0.23	0.81	1.00	1.00	0.95	1.00	0.31	0.75	1.00	0.48	0.32	0.17	1.00	0.34	1.00
Post_l	0.90	1.00	0.72	0.51	0.12	0.22	0.46	0.43	0.20	0.48	1.00	0.44	1.00	0.00	1.00	1.00
Post_r	0.15	0.66	1.00	0.52	1.00	0.26	1.00	0.14	0.34	0.32	0.44	1.00	0.84	0.78	0.09	1.00
Cau_l	0.07	0.32	0.16	0.38	1.00	0.62	1.00	0.23	0.80	0.17	1.00	0.84	1.00	0.86	0.57	0.86
Cau_r	1.00	1.00	0.26	0.37	0.27	0.33	0.10	1.00	0.53	1.00	0.00	0.78	0.86	1.00	0.58	0.86
Put_l	0.23	0.17	1.00	0.81	0.27	0.13	1.00	1.00	1.00	0.34	1.00	0.09	0.57	0.58	1.00	0.72
Put_r	0.63	0.11	0.35	1.00	0.23	0.67	0.23	0.43	0.49	1.00	1.00	1.00	0.86	0.86	0.72	1.00

Supplementary Figure 1. Comparison of the penalties between Atan and Lasso in estimating partial correlations. The Atan penalty effectively shrinks smaller partial correlations towards zero while preserving larger ones, unlike Lasso, which tends to distort the values of partial coefficients. The large partial correlations are heavily penalized with Lasso, whereas this was not so for the atan penalty. This property of non-convex regularization should provide nearly unbiased estimates, which can improve predictive accuracy.

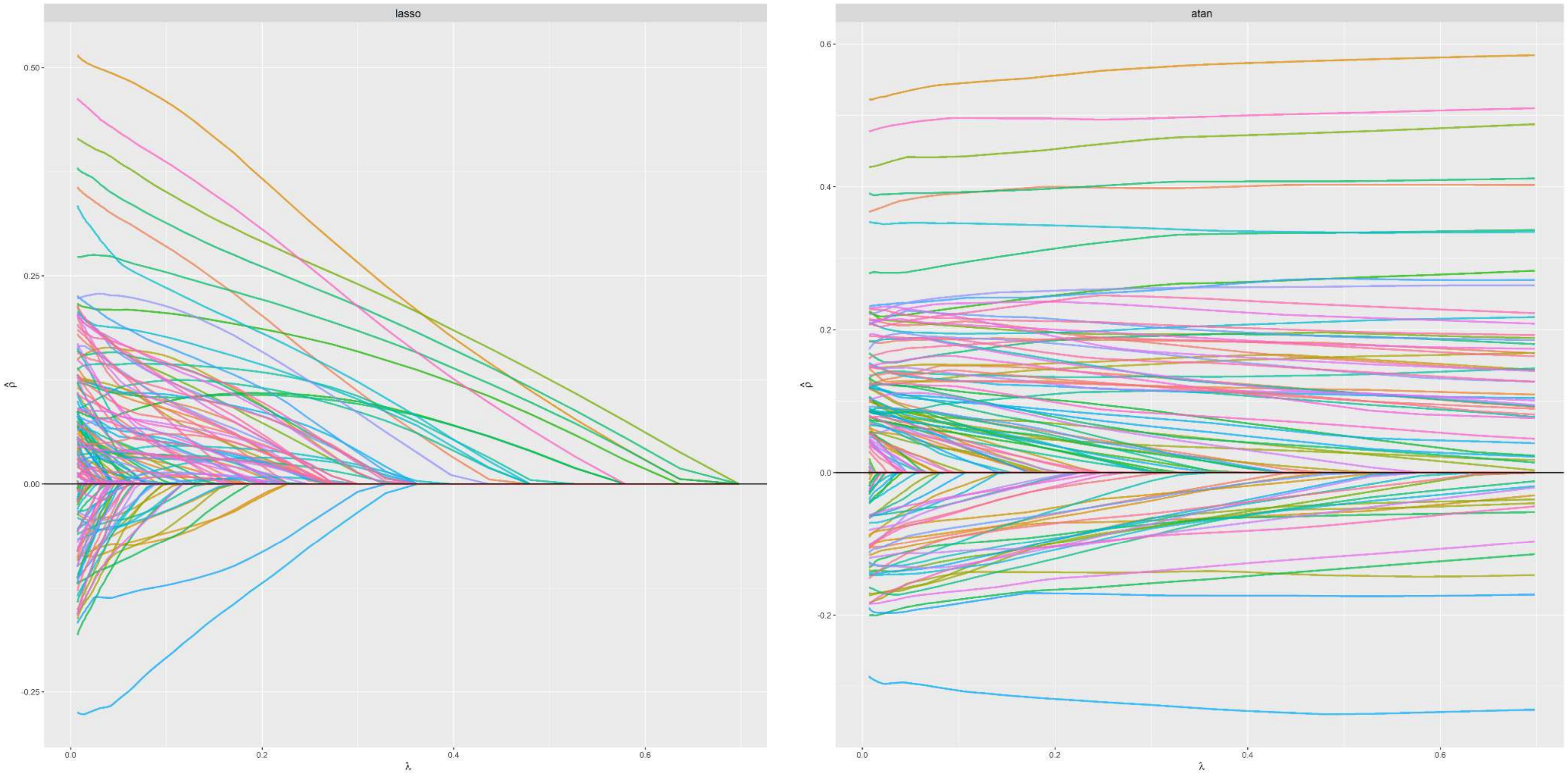

Supplementary Figure 2. Kendall's correlation matrix of the control group. Negative correlations are colored in blue while positive in red. Significant levels are expressed by * (<0.05), ** (<0.01), ***(<0.001).

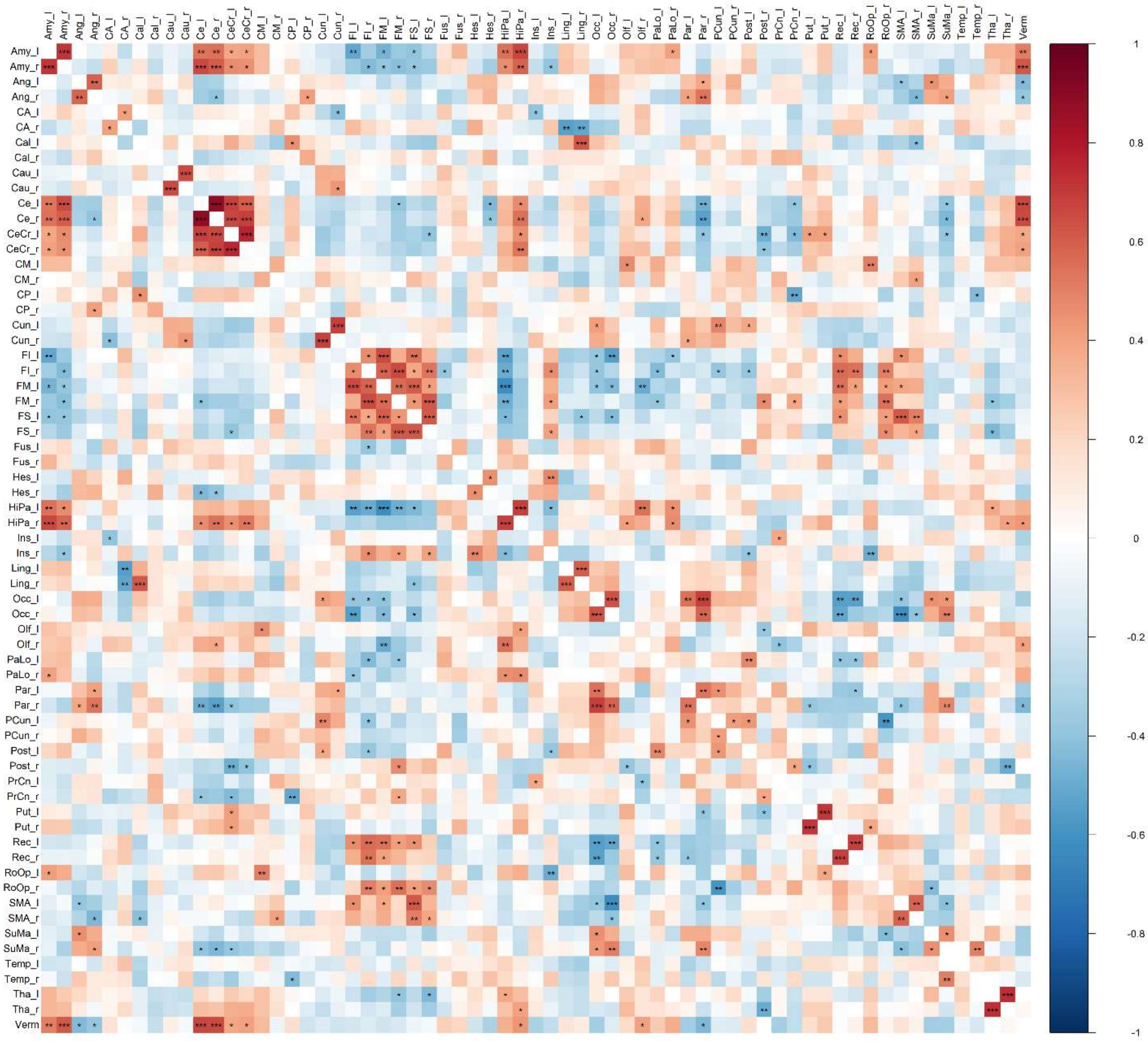

Supplementary Figure 3. Kendall's correlation matrix of the nTRS group. Negative correlations are colored in blue while positive in red. Significant levels are expressed by * (<0.05), ** (<0.01), ***(<0.001).

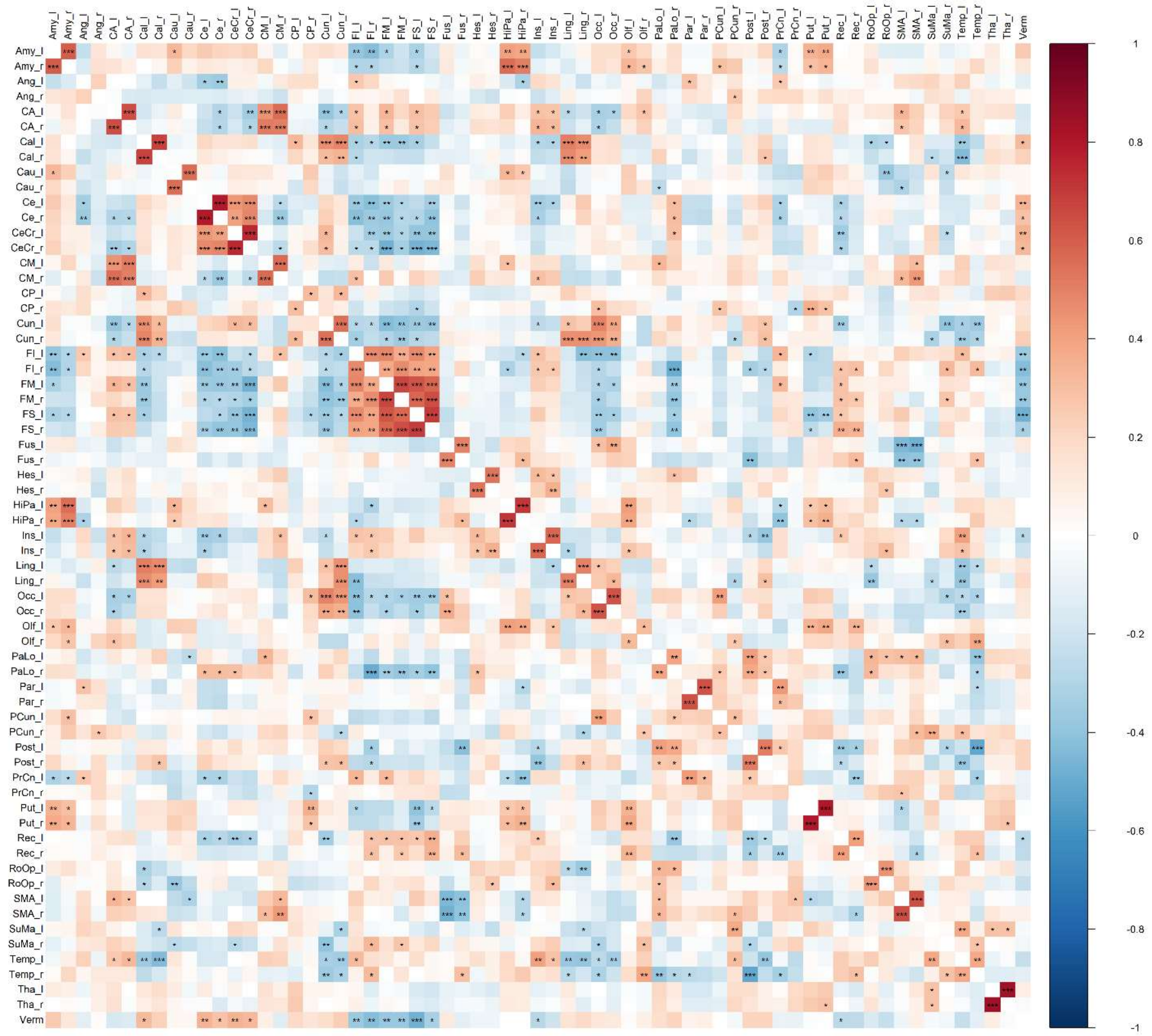

Supplementary Figure 4. Kendall's correlation matrix of the TRS group. Negative correlations are colored in blue while positive in red. Significant levels are expressed by * (<0.05), ** (<0.01), ***(<0.001).

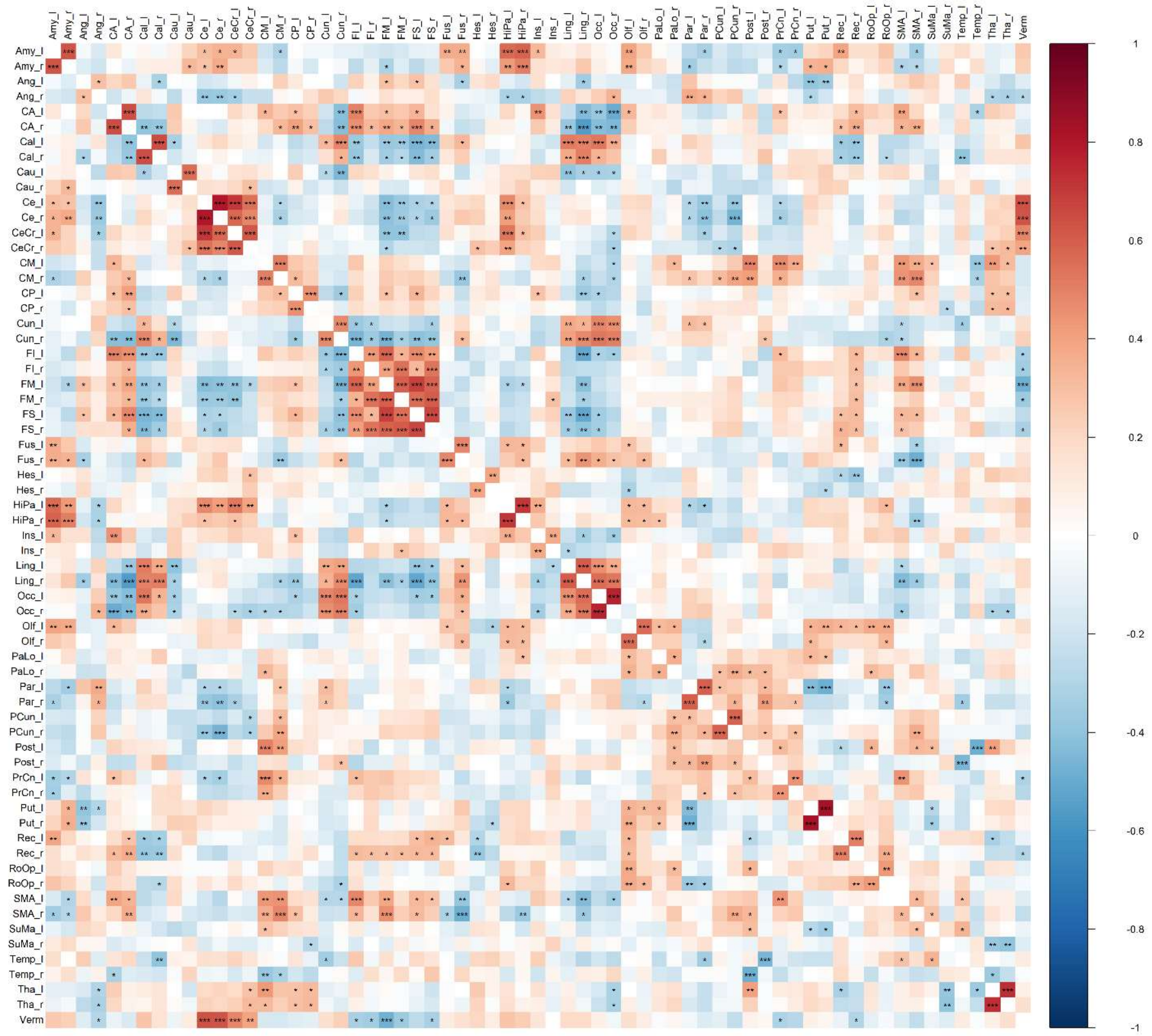

Supplementary Figure 5. Correlation matrix of the control group after regularization. Negative correlations are colored in blue while positive in red. Significant levels are expressed by * (<0.05), ** (<0.01), ***(<0.001).

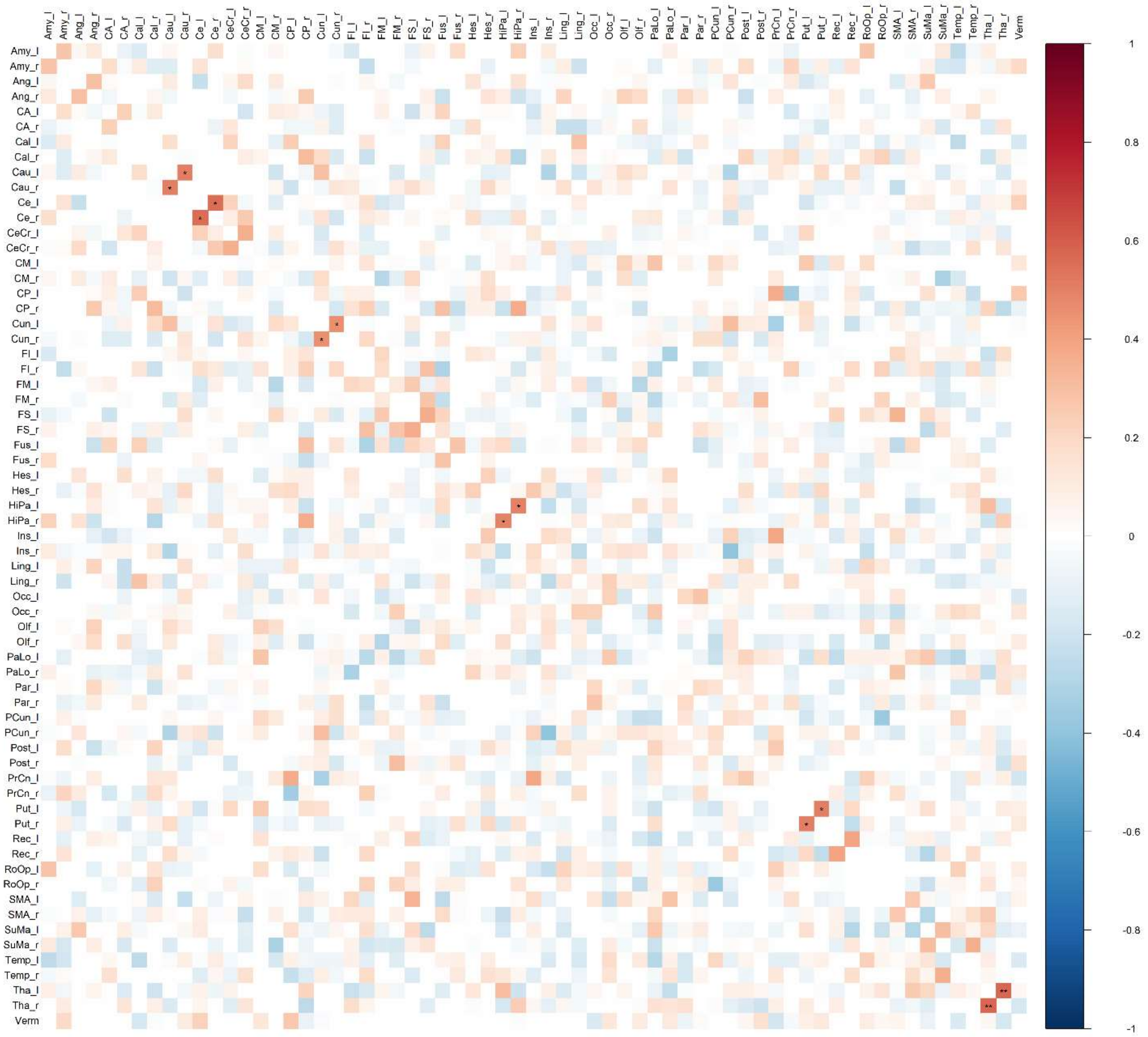

Supplementary Figure 6. Correlation matrix of the nTRS group after regularization. Negative correlations are colored in blue while positive in red. Significant levels are expressed by * (<0.05), ** (<0.01), ***(<0.001).

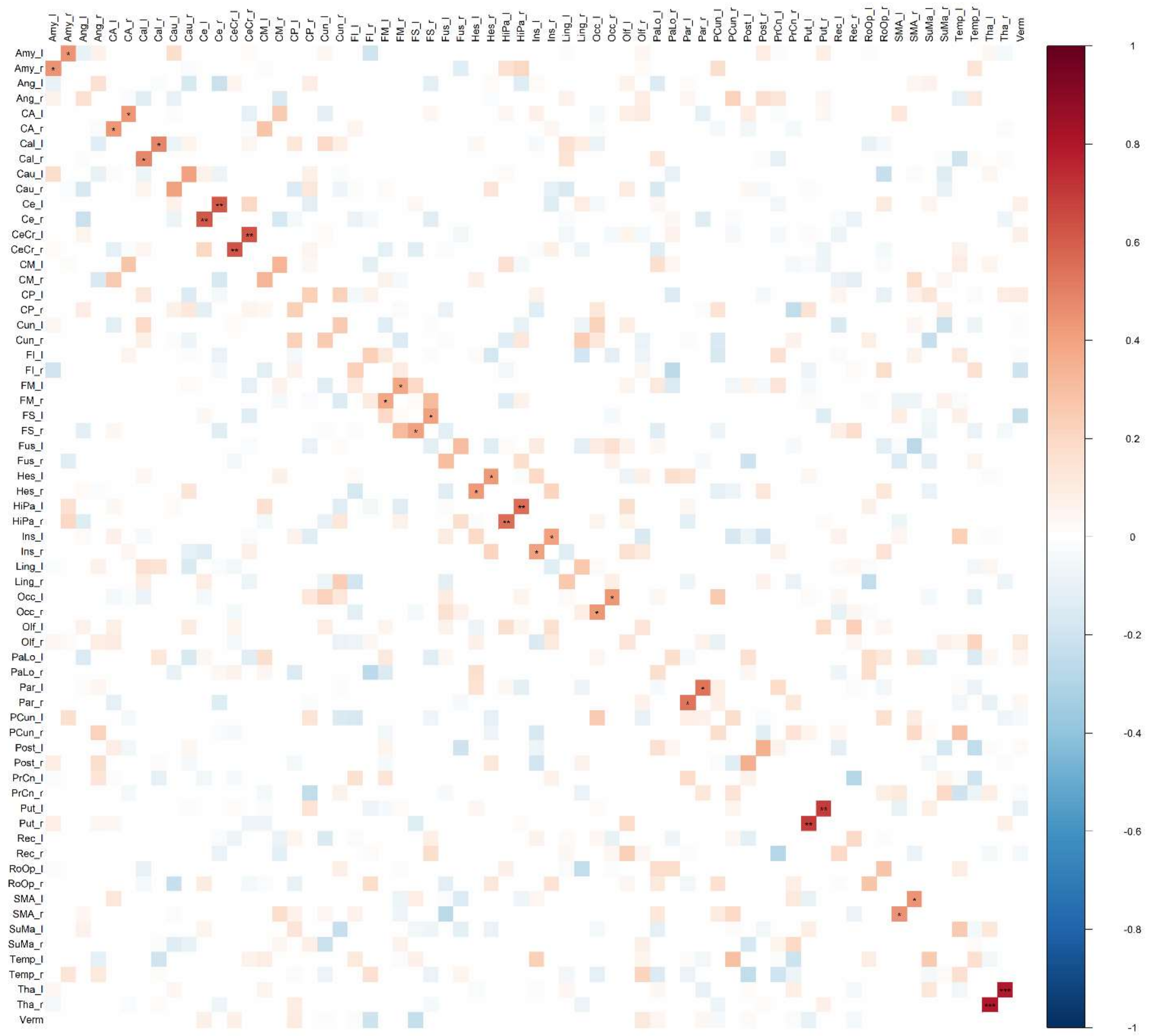

Supplementary Figure 7. Correlation matrix of the TRS group after regularization. Negative correlations are colored in blue while positive in red. Significant levels are expressed by * (<0.05), ** (<0.01), ***(<0.001).

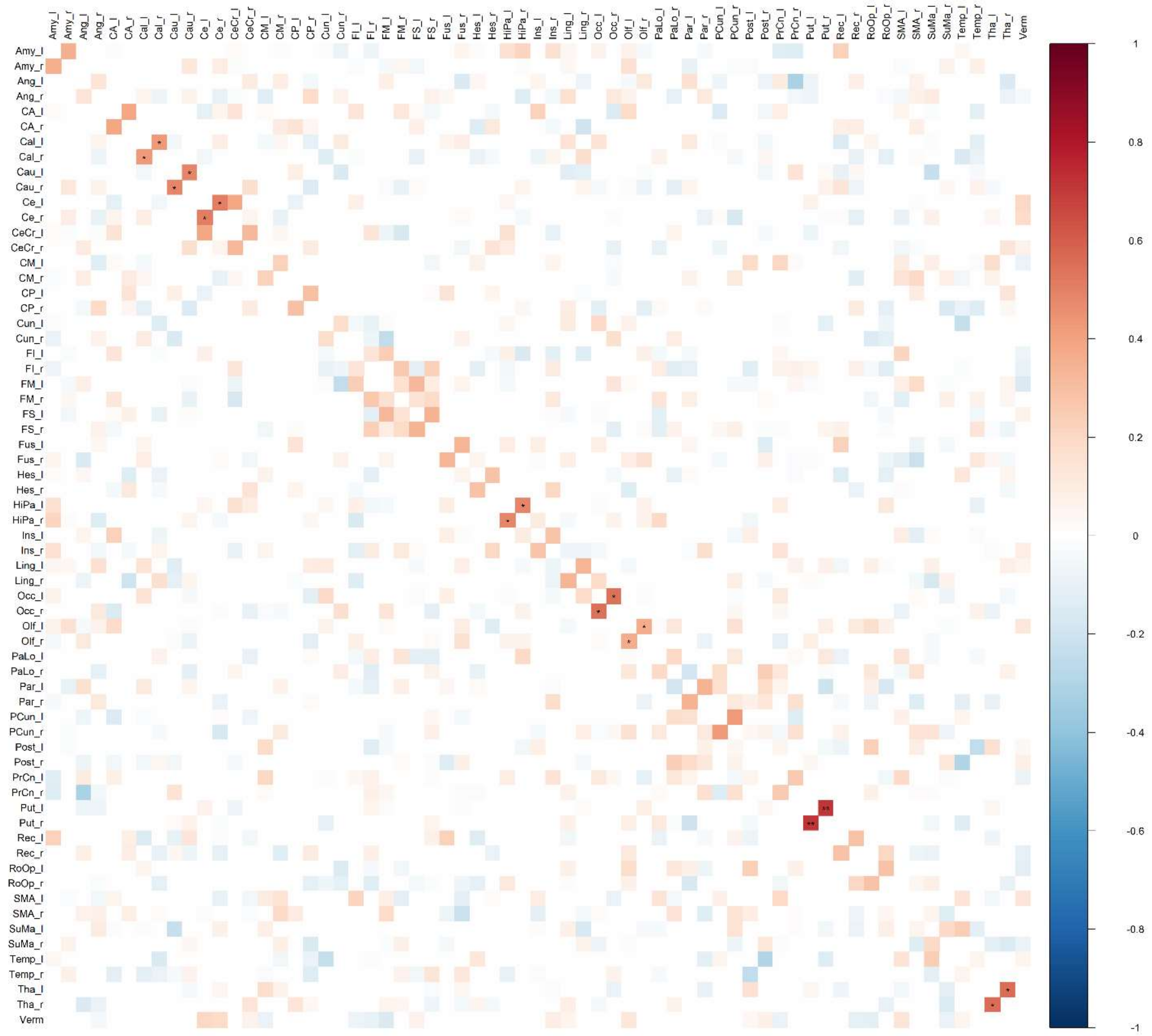

Supplementary Figure 8. Brain plots highlight differences in paired edges across group comparisons. Only significant edges are plotted (purple) across nodes (yellow) comprised in the DMN.

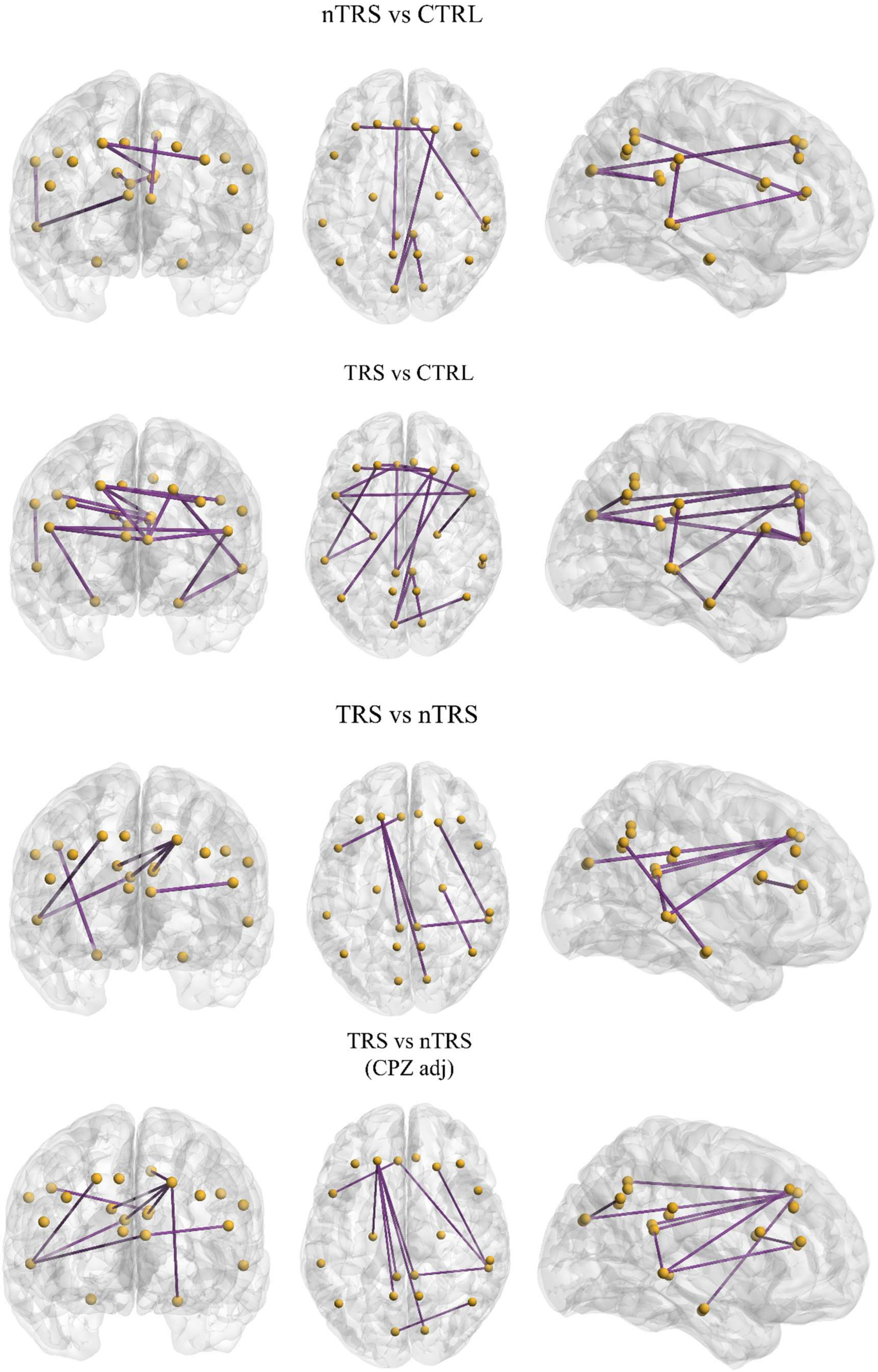

Supplementary Figure 9. Brain plots highlight differences in paired edges across group comparisons. Only significant edges are plotted (light blue) across nodes (yellow) comprised in the dorsal dopamine pathway.

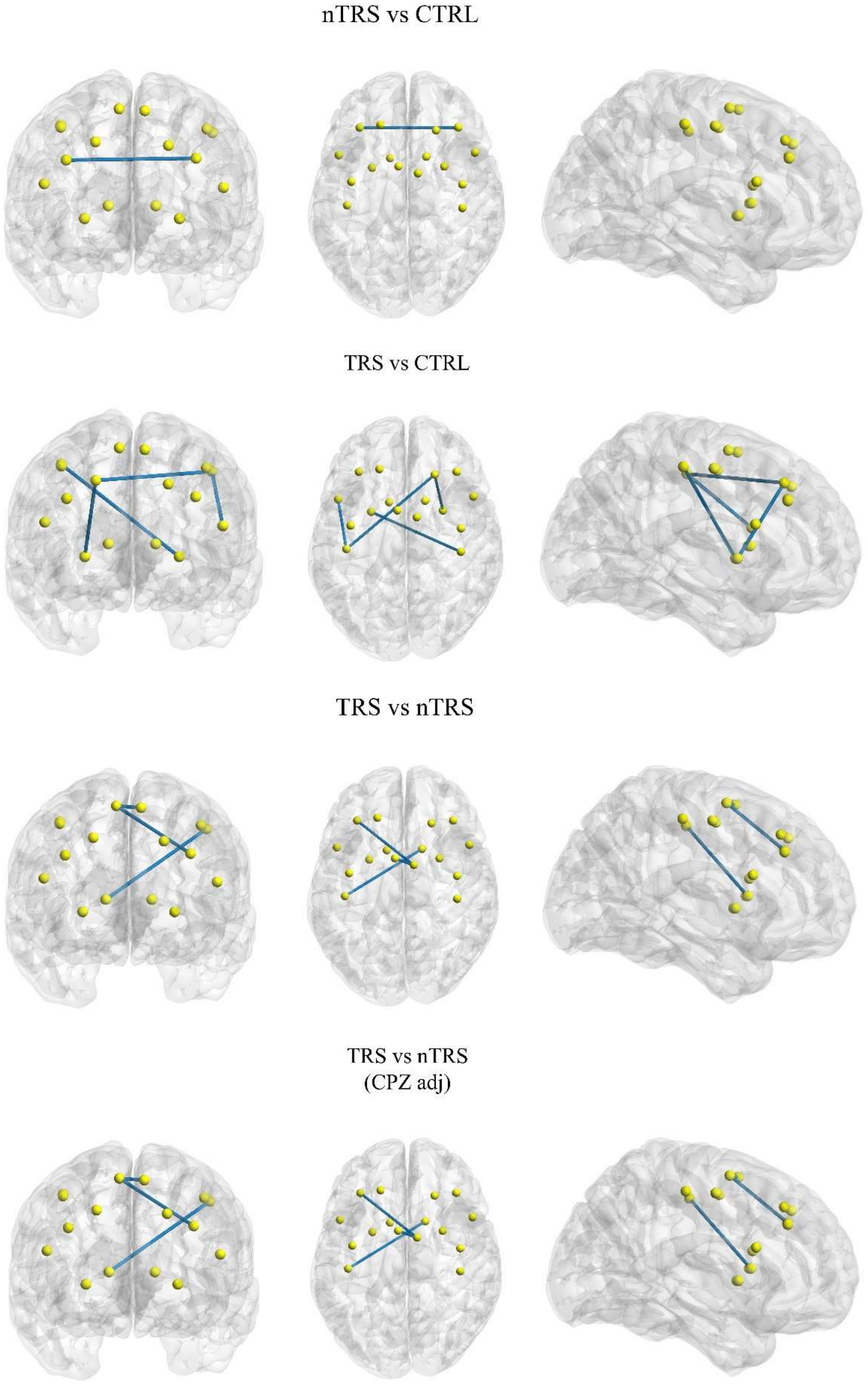

Supplementary Figure 10. Paired edge comparisons between patients and controls, adjusted for age. In the comparison between nTRS and CTRL, significant alterations were observed between the inferior frontal gyrus and the hippocampal area in both hemispheres after controlling for age (permutation p-value = 0.04). In the TRS vs CTRL comparison, significant alterations were found between the left inferior and middle frontal gyri (permutation p-value = 0.03), as well as between the right hippocampus and posterior cingulate (permutation p-value = 0.04). Additionally, a major reorganization involving the DDP was detected. Specifically, alterations in the connections linking the middle frontal gyrus with the putamen (permutation p-value = 0.01) and postcentral gyrus (permutation p-value = 0.02) were observed in the TRS/nTRS vs CTRL comparison. Further, alteration between the precentral gyrus and the supplementary motor area was identified in the TRS vs CTRL comparison (permutation p-value = 0.03). In TRS vs CTRL, alterations involving the superior frontal gyrus were lost within the DDP after controlling for age.

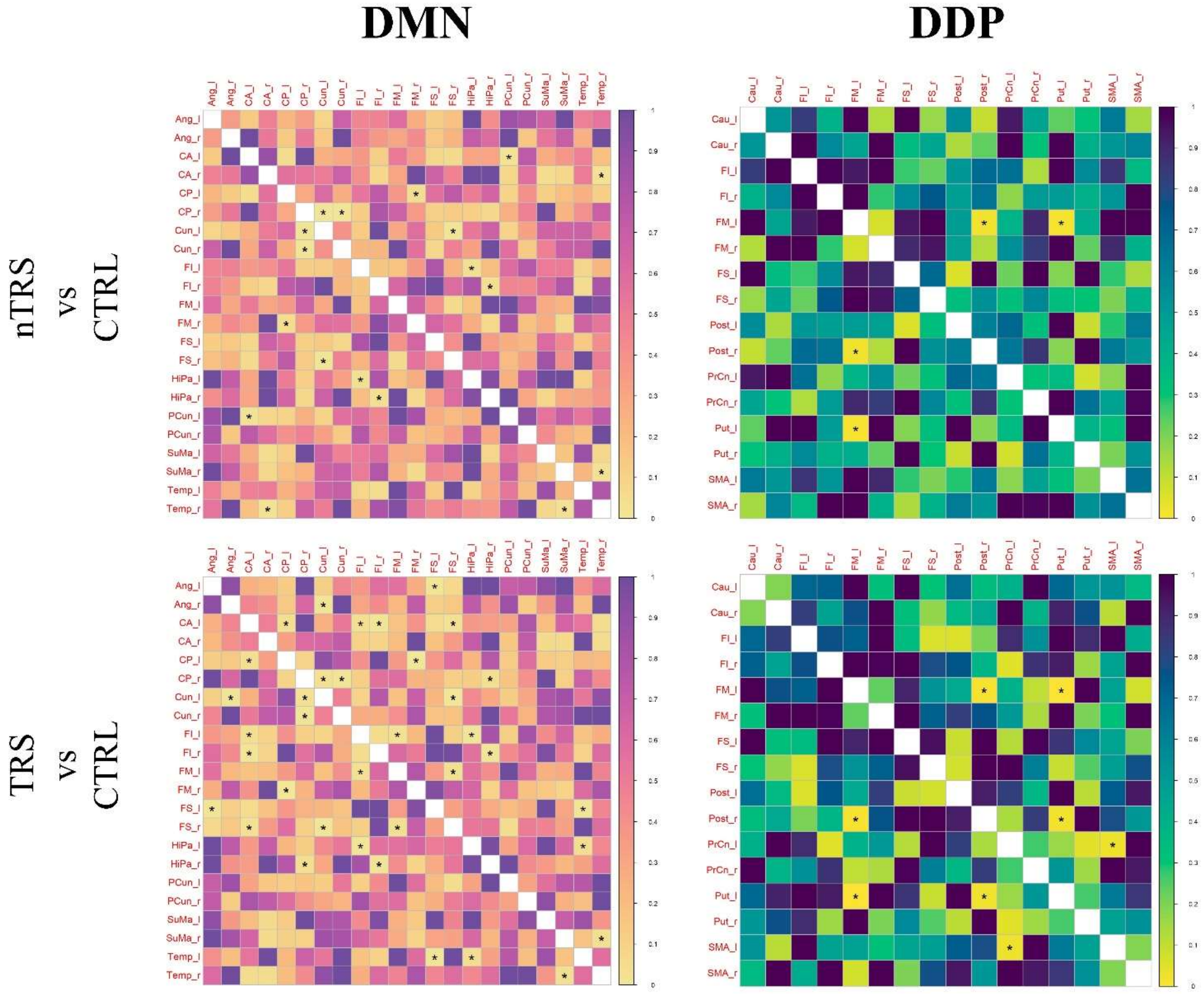

Supplementary Figure 11. Paired edge comparisons between TRS *vs* controls/nTRS, adjusted for sex. Concerning the comparison between TRS and nTRS, results were almost comparable with the ones presented in the main text for both the DMN and DDP. In the comparison between TRS and controls, alterations involving the anterior cingulate and the temporal gyrus in the DMN, and the superior frontal gyrus in the DDP were lost after controlling for sex. Alterations in the connections linking the middle frontal gyrus with the putamen (permutation p-value = 0.01) and postcentral gyrus (permutation p-value = 0.01), as well as between the precentral gyrus and the supplementary motor area (permutation p-value = 0.03), emerged.

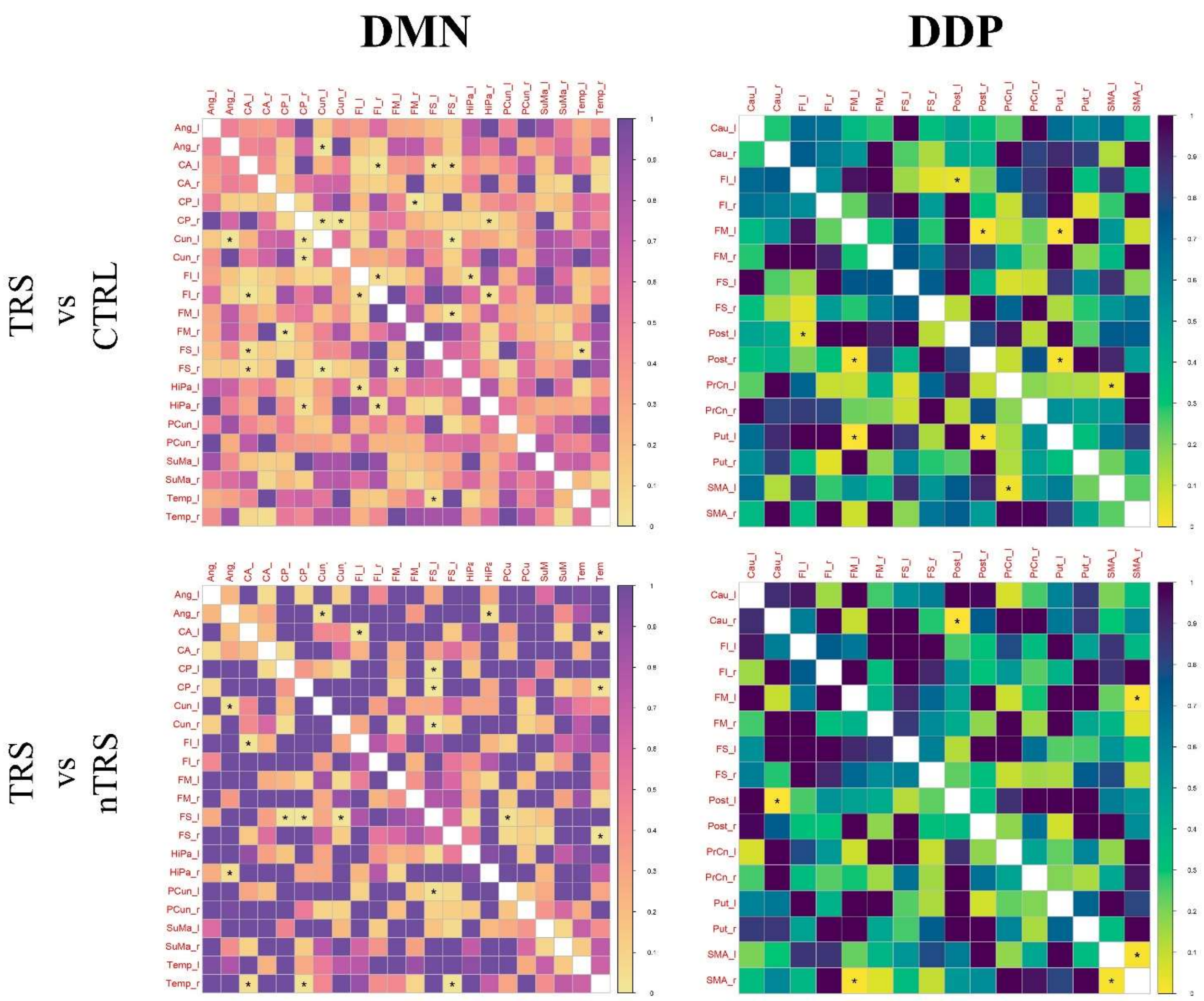

Supplementary Figure 12. Paired edge comparisons between TRS and nTRS, adjusted for years of education. Results are in line with the findings discussed in the main text.

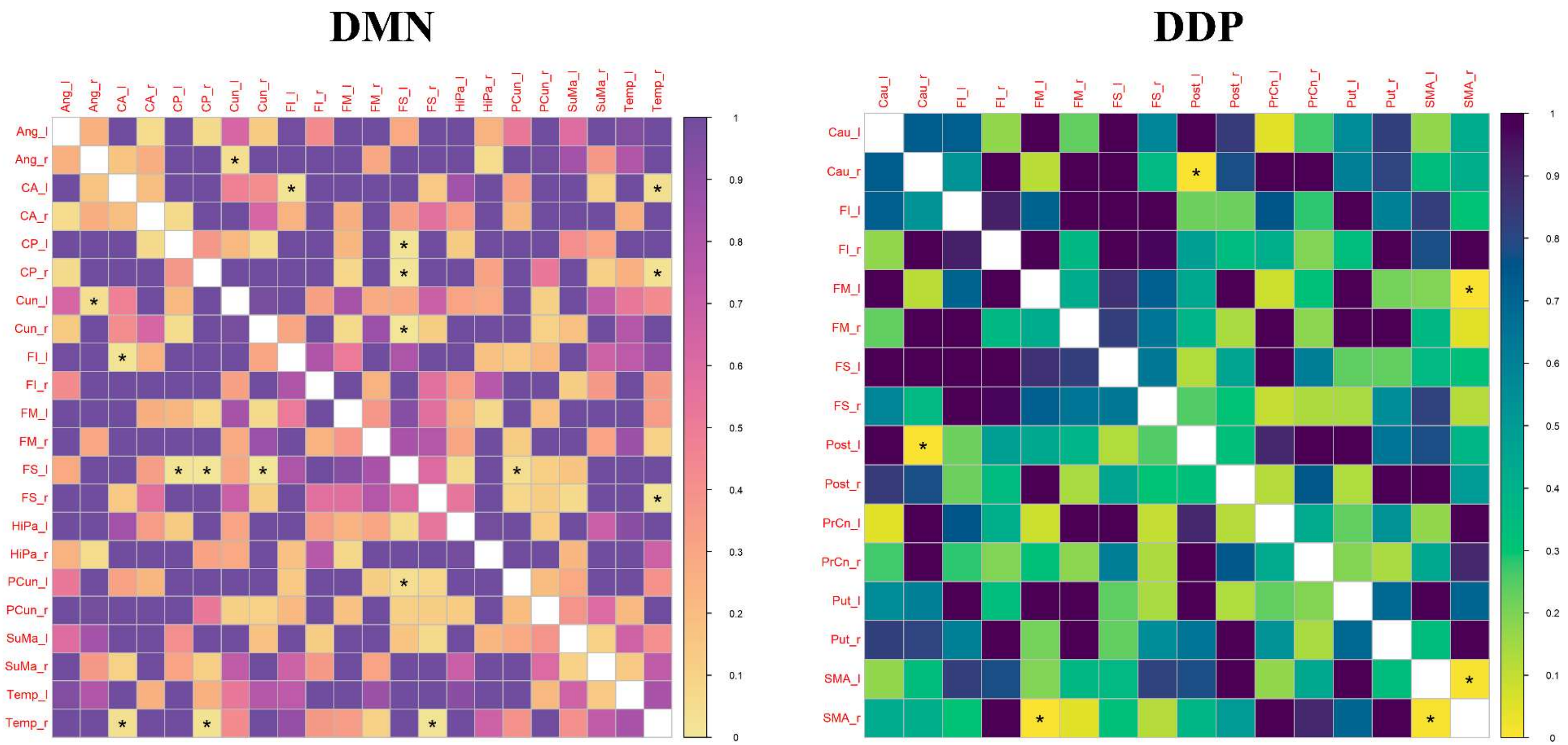

Supplementary Figure 13. Paired edge comparisons between TRS and nTRS, adjusted for illness duration. In addition to findings consistent with those discussed in the main text, a significant alteration in connectivity between the cuneus and posterior cingulate cortex was observed (permuted p-value = 0.03).

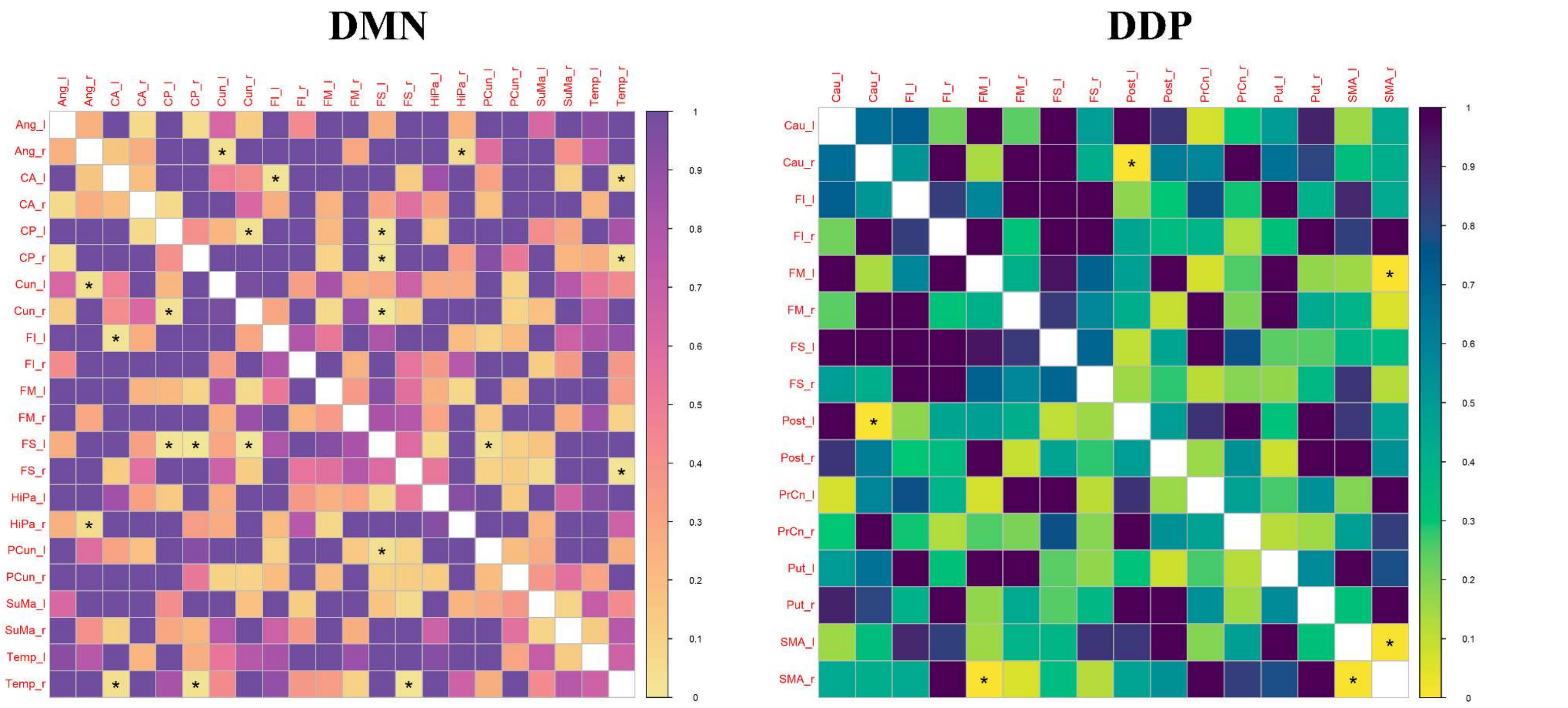

Supplementary Figure 14. Paired edge comparisons between TRS and nTRS, adjusted for age of onset. Alongside findings consistent with those discussed in the main text, a significant alteration in connectivity between the cuneus and posterior cingulate cortex was identified (permuted p-value = 0.04).

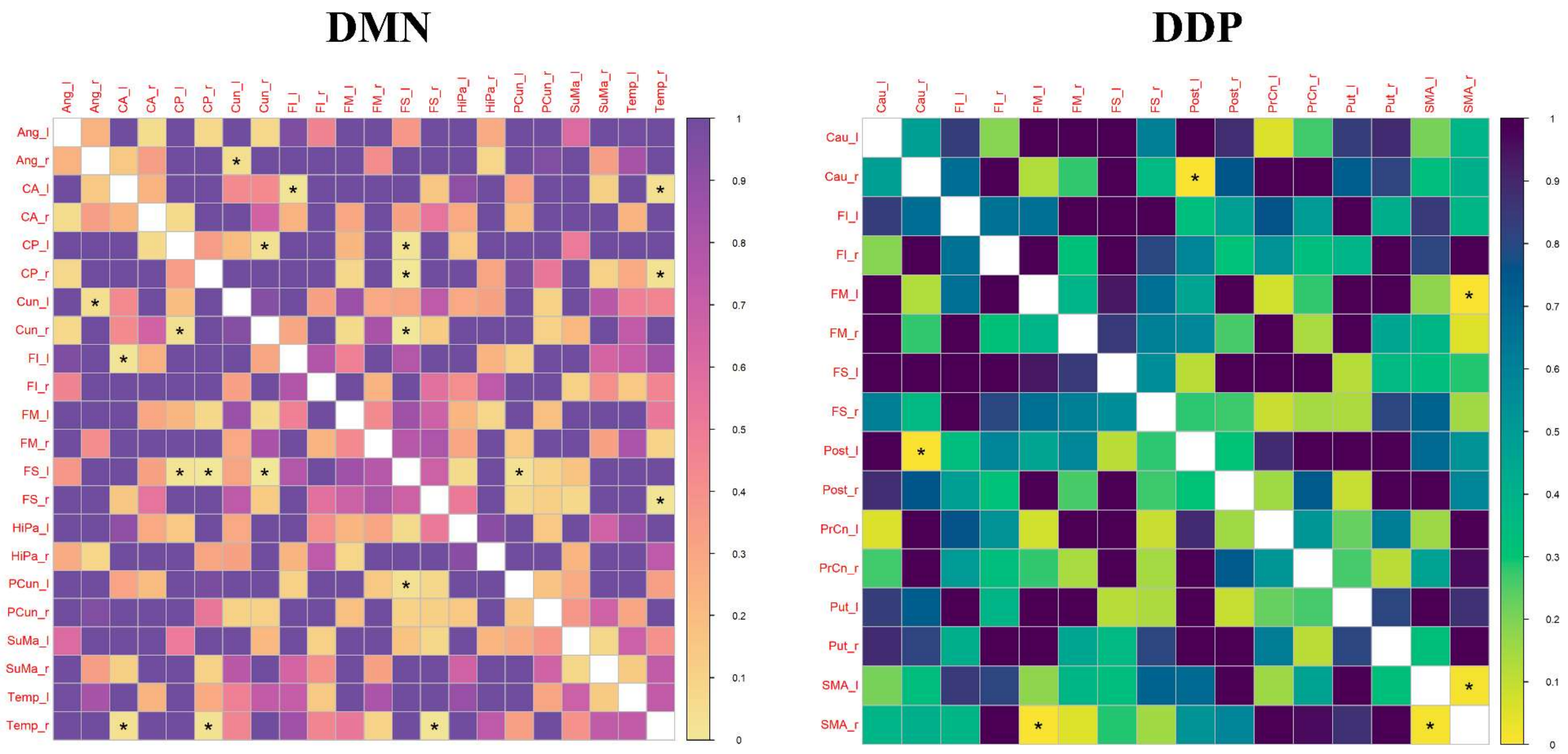

Supplementary Figure 15. Results from nested cross-validation using data of dorsal dopamine pathway regions. Boxplots of model predictors are depicted to show the distribution of glucose signals along the selected ROIs (A). It is shown how deviance is affected by alpha (B) and lambda (C). Variable importance (VIMP score) is represented in D. ROC curves from both outer (E) and inner CV (F) are plotted.

A.

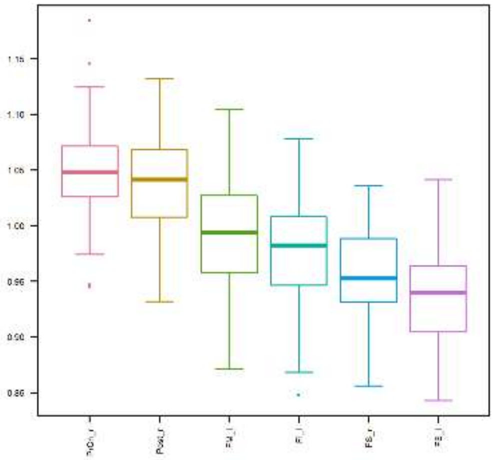

B.

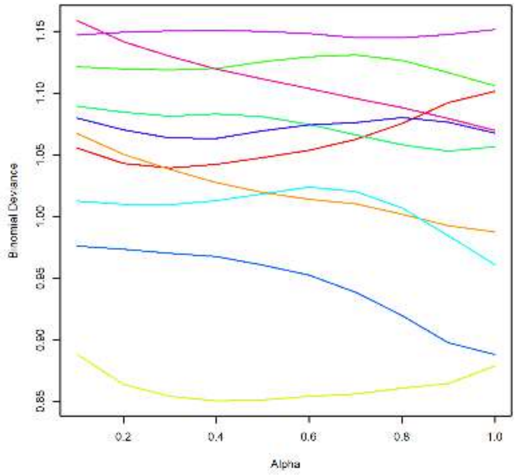

C.

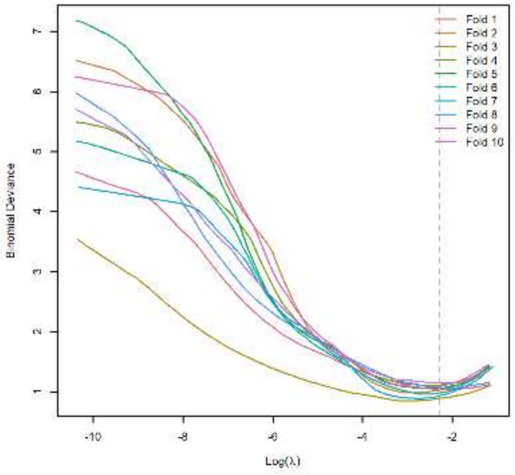

D.

(Intercept)	10.56196
FS_l	-12.7838
Post_r	8.047557
FI_l	-6.01286
FS_r	-4.79917
PrCn_r	4.517812
FM_l	-1.28918

E.

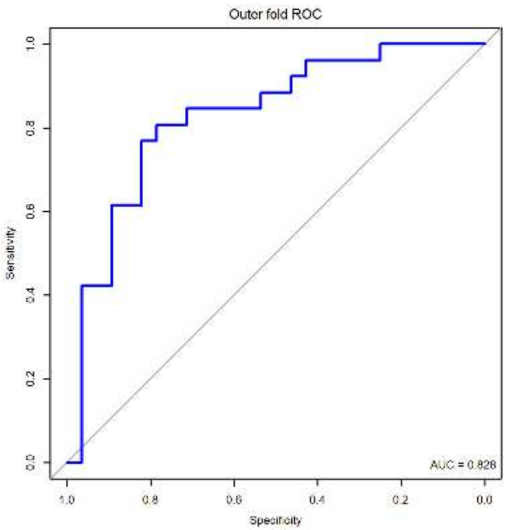

F.

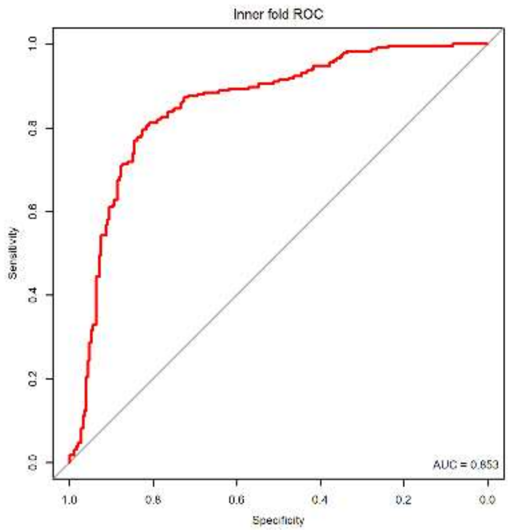

Supplementary Figure 16. Results from nested cross-validation using data of the whole dataset. Boxplots of model predictors are depicted to show the distribution of glucose signals along the selected ROIs (A). It is shown how deviance is affected by alpha (B) and lambda (C). Variable importance (VIMP score) is represented in D. ROC curves from both outer (E) and inner CV (F) are plotted.

A.

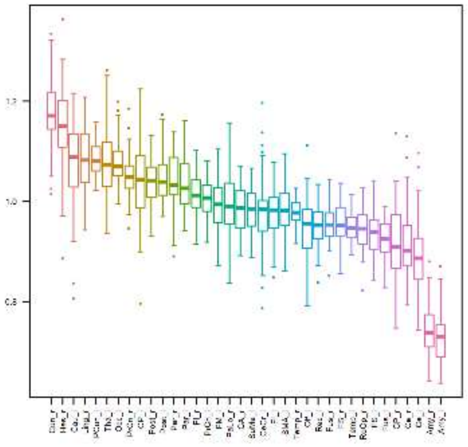

B.

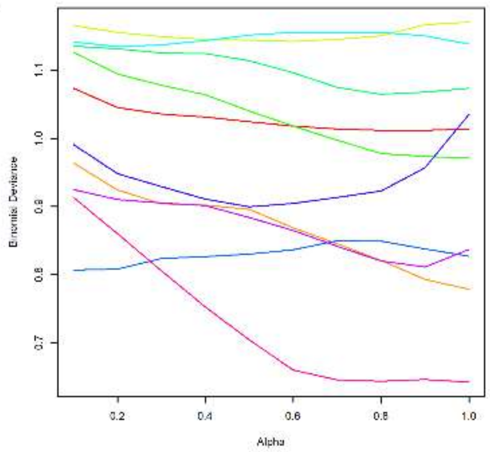

C.

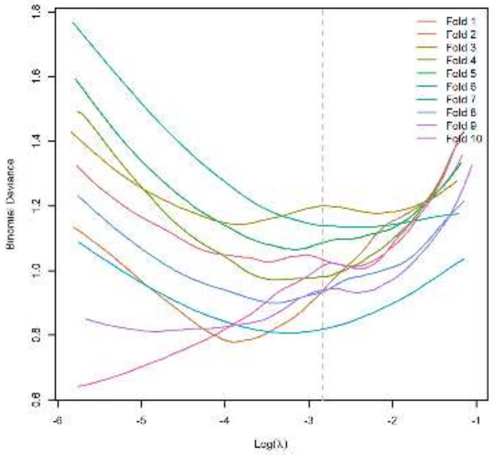

D.

	(Intercept) -14.828		
Fus_r	16.83207	PrCn_l	3.252304
Post_r	12.41273	RoOp_r	2.918078
Temp_r	11.78732	FM_l	-2.8751
FS_l	-11.5574	Ce_l	2.748086
FI_l	-10.6759	CeCr_l	2.22445
PrCn_r	8.112468	CP_l	2.045749
Cun_r	-7.40037	Olf_l	-1.87704
FS_r	-7.17461	Ling_r	1.786307
Post_l	6.675202	Par_l	-1.74277
Amy_l	-6.48644	Hes_r	-1.69082
SuMa_l	-6.34721	SMA_l	1.436717
Fus_l	5.634746	Ce_r	1.297843
CA_r	5.34141	CP_r	1.102779
FI_r	-4.75482	Cau_l	0.978428
Temp_l	4.437582	Par_r	-0.88903
Tha_l	-3.9957	PaLo_r	-0.54034
Occ_l	-3.99096	Amy_r	-0.38988
Rec_l	-3.73224	PCun_l	-0.27169

E.

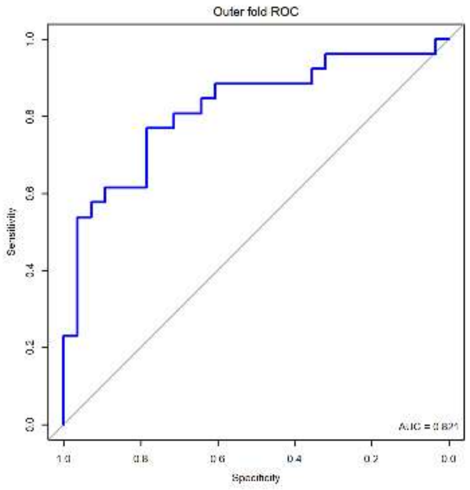

F.

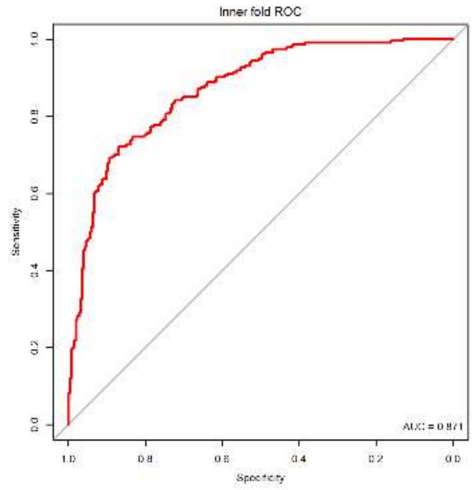

Supplement: Supplementary file 1 — Supplementary Information [file 41537_2024_535_MOESM1_ESM.pdf]
